# Mitochondrial calcium uniporter complex controls T-cell-mediated immune responses

**DOI:** 10.1038/s44319-024-00313-4

**Published:** 2024-12-02

**Authors:** Magdalena Shumanska, Dmitri Lodygin, Christine S Gibhardt, Christian Ickes, Ioana Stejerean-Todoran, Lena C M Krause, Kira Pahl, Lianne J H C Jacobs, Andrea Paluschkiwitz, Shuya Liu, Angela Boshnakovska, Niels Voigt, Tobias J Legler, Martin Haubrock, Miso Mitkovski, Gereon Poschmann, Peter Rehling, Sven Dennerlein, Jan Riemer, Alexander Flügel, Ivan Bogeski

**Affiliations:** 1 Molecular Physiology, Institute of Cardiovascular Physiology, University Medical Centre, Georg-August-University, Göttingen, Germany; 2 Institute for Neuroimmunology and Multiple Sclerosis Research, University Medical Centre, Georg-August-University, Göttingen, Germany; 3 Redox Metabolism, Institute of Biochemistry and CECAD, University of Cologne, Cologne, Germany; 4 Department of Cellular Biochemistry, University Medical Centre, Georg-August-University, Göttingen, Germany; 5 Institute of Pharmacology and Toxicology, University Medical Centre, Georg-August-University, Göttingen, Germany; 6 Department of Transfusion Medicine, University Medical Centre, Göttingen, Germany; 7 Department of Medical Bioinformatics, University Medical Centre, Georg-August-University, Göttingen, Germany; 8 City Campus Light Microscopy Facility, Max Planck Institute for Multidisciplinary Sciences, Göttingen, Germany; 9 Institute for Molecular Medicine, Proteome Research, University Hospital and Medical Faculty, Heinrich-Heine-University, Düsseldorf, Germany

**Keywords:** Autoimmunity, Calcium, MCU, Mitochondria, T-cell, Immunology, Membranes & Trafficking, Metabolism

## Abstract

T-cell receptor (TCR)-induced Ca^2+^ signals are essential for T-cell activation and function. In this context, mitochondria play an important role and take up Ca^2+^ to support elevated bioenergetic demands. However, the functional relevance of the mitochondrial-Ca^2+^-uniporter (MCU) complex in T-cells was not fully understood. Here, we demonstrate that TCR activation causes rapid mitochondrial Ca^2+^ (_m_Ca^2+^) uptake in primary naive and effector human CD4^+^ T-cells. Compared to naive T-cells, effector T-cells display elevated _m_Ca^2+^ and increased bioenergetic and metabolic output. Transcriptome and proteome analyses reveal molecular determinants involved in the TCR-induced functional reprogramming and identify signalling pathways and cellular functions regulated by MCU. Knockdown of MCUa (MCUa_KD_), diminishes _m_Ca^2+^ uptake, mitochondrial respiration and ATP production, as well as T-cell migration and cytokine secretion. Moreover, MCUa_KD_ in rat CD4^+^ T-cells suppresses autoimmune responses in an experimental autoimmune encephalomyelitis (EAE) multiple sclerosis model. In summary, we demonstrate that _m_Ca^2+^ uptake through MCU is essential for proper T-cell function and has a crucial role in autoimmunity. T-cell specific MCU inhibition is thus a potential tool for targeting autoimmune disorders.

## Synopsis

The therapeutic potential of mitochondrial calcium (Ca^2+^) homeostasis remains largely unexplored. This study demonstrates that the mitochondrial Ca^2+^ uniporter (MCU) complex is critical for T-cell function, emphasizing the potential of targeting _m_Ca^2+^ homeostasis as a therapeutic approach for managing autoimmune diseases.

- CD4^+^ T-cell activation causes elevated _m_Ca^2+^ uptake and enhanced mitochondrial bioenergetic output.
- MCU plays a key role in controlling mitochondrial function in both human and rat CD4^+^ T-cells.
- Suppression of the MCUa subunit diminishes T-cell respiration, cytokine secretion, migration and invasion.
- Effector T-cells lacking MCUa display decreased autoreactive immune responses in vivo.

## Abstract (web summary)

The therapeutic potential of mitochondrial calcium (Ca^2+^) homeostasis remains largely unexplored. This study demonstrates that the mitochondrial Ca^2+^ uniporter (MCU) complex is critical for T-cell function, emphasizing the potential of targeting _m_Ca^2+^ homeostasis as a therapeutic approach for managing autoimmune diseases.

## Graphical abstract

**Figure:**
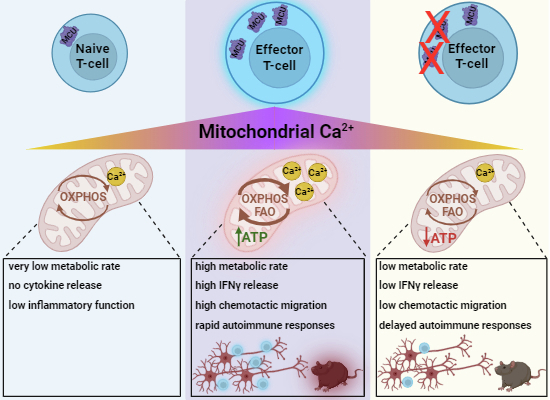

**Figure:**
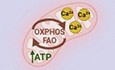

## Introduction

T-helper cells (CD4^+^ T-cells) control and maintain adaptive immune responses against a variety of pathogens. They are also involved in autoimmunity, asthma and allergic responses, and support anti-tumour immunity. These functions are mediated by effector T-helper cell subsets such as Th1, Th2, Th9, Th17, Th22 and Treg cells, among others, which differentiate from naive T-cells depending on the cytokine milieu and other metabolic, environmental and genetic factors (Zhu and Paul, 2008). Naive CD4^+^ T-cell activation occurs as a coordinated interaction between a T-cell receptor (TCR) and an antigen-major histocompatibility (MHC) class II complex, presented by an antigen-presenting cell. Several downstream signalling cascades are initiated upon TCR stimulation, including store-operated calcium entry (SOCE), mediated by STIM-gated ORAI channels (Prakriya and Lewis, 2015). In T-cells, SOCE regulates activation of transcription factors and is thus responsible for controlling functions such as clonal expansion, cytokine production and migration (Diercks et al, 2018; Hogan et al, 2010; Srikanth et al, 2017). Mitochondria, which are known to take up Ca^2+^ during SOCE, also play an essential role in T-cell biology (Buck et al, 2016; Desdín-Micó et al, 2020; Ron-Harel et al, 2016; Sena et al, 2013). The contribution of mitochondria-controlled metabolic pathways, such as one-carbon metabolism, amino acid and lipid metabolism, reactive oxygen species (ROS) signalling, as well as oxidative phosphorylation, is of central importance (Berod et al, 2014; Ron-Harel et al, 2016; Simeoni and Bogeski, 2015; Steinert et al, 2021).

Ca^2+^ entry across the plasma membrane and/or Ca^2+^ release from intracellular stores initiate mitochondrial Ca^2+^ uptake (Mammucari et al, 2018; Pathak and Trebak, 2018; Voeltz et al, 2024). The voltage-dependent anion channels (VDAC) transport Ca^2+^ across the outer mitochondrial membrane (OMM), while the mitochondrial Ca^2+^ uniporter (MCU) complex is responsible for Ca^2+^ transport across the inner mitochondrial membrane (IMM).

The MCU complex is a macro-complex consisting of a pore-forming unit, a gate-keeping unit, and several channel regulatory proteins (Baughman et al, 2011; De Stefani et al, 2011; Perocchi et al, 2010). The pore of the channel comprises MCUa and MCUb subunits which share around 50% homology. MCUb contains a crucial amino acid substitution in the loop region which highly decreases the conductivity of the channel. While MCUa facilitates Ca^2+^ transport, MCUb is a negative regulator of MCUa, leading to a decreased _m_Ca^2+^ uptake (Colussi and Stathopulos, 2023). Mitochondrial calcium uptake proteins 1, 2 and 3 (MICU1-3), located in the intermembrane space, are characterised as the main gate-keeping proteins of the MCU complex, controlling the extent of Ca^2+^ ion uptake through the pore of the channel (Petrungaro et al, 2015). MICU1 is highly conserved among species; it keeps the MCU channel closed when the cytosolic Ca^2+^ (_c_Ca^2+^) levels are below a certain threshold and opens the channel when those levels increase. MICU2 is a paralog of MICU1 (Plovanich et al, 2013). The N-terminus of MICU2 is drastically different when compared with MICU1, affecting the three-dimensional structure of the protein and its functional role in regulating _m_Ca^2+^ uptake (Patron et al, 2014), specifically negatively regulating the function of MICU1 and preventing Ca^2+^ overload. MICU3 is a tissue-specific paralog of MICU1 with a similar function (Patron et al, 2019). The Essential MCU Regulator (EMRE) protein is a regulatory component modulating the interaction between MCUa and MICU1 and allowing for proper channel function. The mitochondrial Ca^2+^ uniporter regulator 1 (MCUR1) is an integral protein that is likely involved in controlling proper MCU complex assembly (Sancak et al, 2013).

Within the mitochondrial matrix, Ca^2+^ ions regulate various enzymes involved in the electron transport chain and modulate both mitochondrial respiration and bioenergetic output (Denton, 2009; Santo-Domingo and Demaurex, 2010). Hence, mitochondria act as central regulatory hubs that integrate mitochondrial Ca^2+^ dynamics with cellular metabolism and T-cell function.

Despite its functional importance, the role of MCU in T-cell physiology and relevant pathologies is not fully understood. Here, we use a panel of experimental and bioinformatic tools to decipher the role of MCU in CD4^+^ T-cells. We utilise primary human CD4^+^ T-cells for in vitro analyses, as well as primary rat CD4^+^ T-cells for exploring the role of MCU in vivo. Our results indicate that MCU is an essential regulator of T-cell function and T-cell-induced autoimmune responses.

## Results

### T-cell activation increases mitochondrial Ca^2+^ uptake via MCU

Functional evaluation of the mitochondrial Ca^2+^ uniporter (MCU) complex in T-cells requires a reliable experimental model that would ideally comprise human as well as murine T-cells for in vivo evaluation. To establish such an experimental system, we utilised naive CD4^+^ T-cells, isolated from anonymous healthy human donors (purity of isolated cells higher than 97%, Fig. EV1A and Appendix Fig. S1). The naive T-cells were activated with anti-CD3/CD28-coated magnetic beads or anti-CD3/CD28 antibody cocktail solution. The efficiency of the two stimulation approaches was evaluated by measuring store-operated Ca^2+^ entry (SOCE) and cell proliferation. As displayed in Fig. EV1B,C, the magnetic beads induced higher SOCE as well as higher cell proliferation when compared with antibody solution stimulation. Based on these observations and given that beads more closely resemble physiological cell stimulation forming an immunological synapse, we selected this approach for generating our effector T-cell model.

**Figure EV1:**

Cytosolic and mitochondrial Ca^2+^ levels are increased upon T-cell activation. (**A**) Pure CD4^+^ naive T-cell population isolated from blood of healthy human donors. Naive T-cells mainly exhibited positive CD4 (PE-A) staining, and not CD8. 99% and 96.9% of cells of from donor 1 (left) and donor 2 (right) were CD4^+^, respectively. The histograms were generated from 10,000 events after gating. The gating strategy for flow cytometry is shown in Appendix Fig. S1. Legend: Grey=unstained control; Green=CD8 staining; Blue = CD4 staining. (**B**) Fura-2 measurements of _c_Ca^2+^ in naive CD4^+^ T-cells treated with anti-CD3/CD28-coated magnetic beads (1:5 bead:cell ratio) or antibody solution (1:1000 working solution). Quantification shown as mean ± SD of 1479 bead-activated and 1,656 antibody solution-activated T-cells/biological replicates from 5 healthy donors. ****p < 0.0001 (*p* = 1.34872E−74), assessed by two-tailed unpaired Student’s t-test. (**C**) Naive T-cell proliferation following beads or antibody solution stimulation. Quantification shown as mean ± SEM of 8 healthy donors/biological replicates. ***p < 0.001 (*p* = 0.0006); **p < 0.01 (*p* = 0.0039), assessed by two-tailed paired Student’s t-test. RFU = relative fluorescence units. (**D**) Fura-2 measurements of _c_Ca^2+^ in naive and effector T-cells. The SERCA-blocker thapsigargin (Tg; 1 μM) was used to deplete ER Ca^2+^ stores and activate SOCE. (**E**) Quantification of data given in (**D**). Data show mean ± SD of 768 naive T-cells and 577 effector T-cells/biological replicates from 3 healthy donors, normalised to their respective cell volume (in µm^3^) as shown in (**G**). ****p < 0.0001 (*p* = 2.73994E−12), assessed by two-tailed unpaired Student’s t-test. (**F**) Representative 3D images of a naive (left) and an effector (right) T-cell. Using pixel classification in Imaris v10.1.0, the cell surface (green) and the mitochondria (red) were created (refer to methods for more details). Quantification of cellular (**G**) and mitochondrial (**H**) volume in naive and effector T-cells showing a mean ± SEM of 66 naive and 52 effector T-cells/biological replicates from 3 healthy donors. Cell volume was quantified by using Machine Learning-based segmentation. ****p < 0.0001 (*p* = 2.40289E−45) and (*p* = 2.3739E−37), assessed by two-tailed unpaired Student’s t-test. (**I**) Measurements of _m_Ca^2+^ levels with the 4mtTNXL biosensor in human naive and effector T-cells. Quantification plots show an average of 118 naive and 331 effector cells/biological replicates from 2 different donors. Cell stimulation was achieved by addition of 1 µM Tg at the indicated time point. The quantification/violin plots show mean ± SD of basal _m_Ca^2+^ levels and maximal _m_Ca^2+^ uptake. ****p < 0.0001 (Basal *p* = 1.6523E−33; Uptake *p* = 8.3596E−16), assessed by two-tailed unpaired Student’s t-test. (**J**) Measurements of _m_Ca^2+^ levels with the 4mtTNXL biosensor in human naive and effector T-cells. Traces show an average of 4 naive and 16 effector cells/biological replicates from 1 healthy donor. Cells were stimulated with anti-human CD3/CD28-coated beads at the indicated time point. The quantification/violin plots show mean ± SD for both the basal _m_Ca^2+^ levels and _m_Ca^2+^ uptake. ***p < 0.001 (*p* = 0.0004). (**K**) Calcium Retention Capacity (CRC) assay measuring _m_Ca^2+^ uptake in digitonin-permeabilized naive and effector T-cells using Calcium-Green-5N. Traces show mean ± SEM relative fluorescence units (RFU) of 10 naive and 10 effector T-cells/biological replicates from 3 healthy donors (assays always performed at least in duplicate). _m_Ca^2+^ uptake was induced by subsequent additions of a bolus of Ca^2+^ at the indicated time points (black arrows). The quantification plots show mean ± SEM for basal _m_Ca^2+^ uptake at the time points indicated by a red star. _m_Ca^2+^ uptake was quantified as the delta decrease in measured RFU upon Ca^2+^ addition. *p ≤ 0.05 (*p* = 0.0481-left; *p* = 0.0443-right panel), assessed by two-tailed unpaired Student’s t-test.

**Figure 1:**
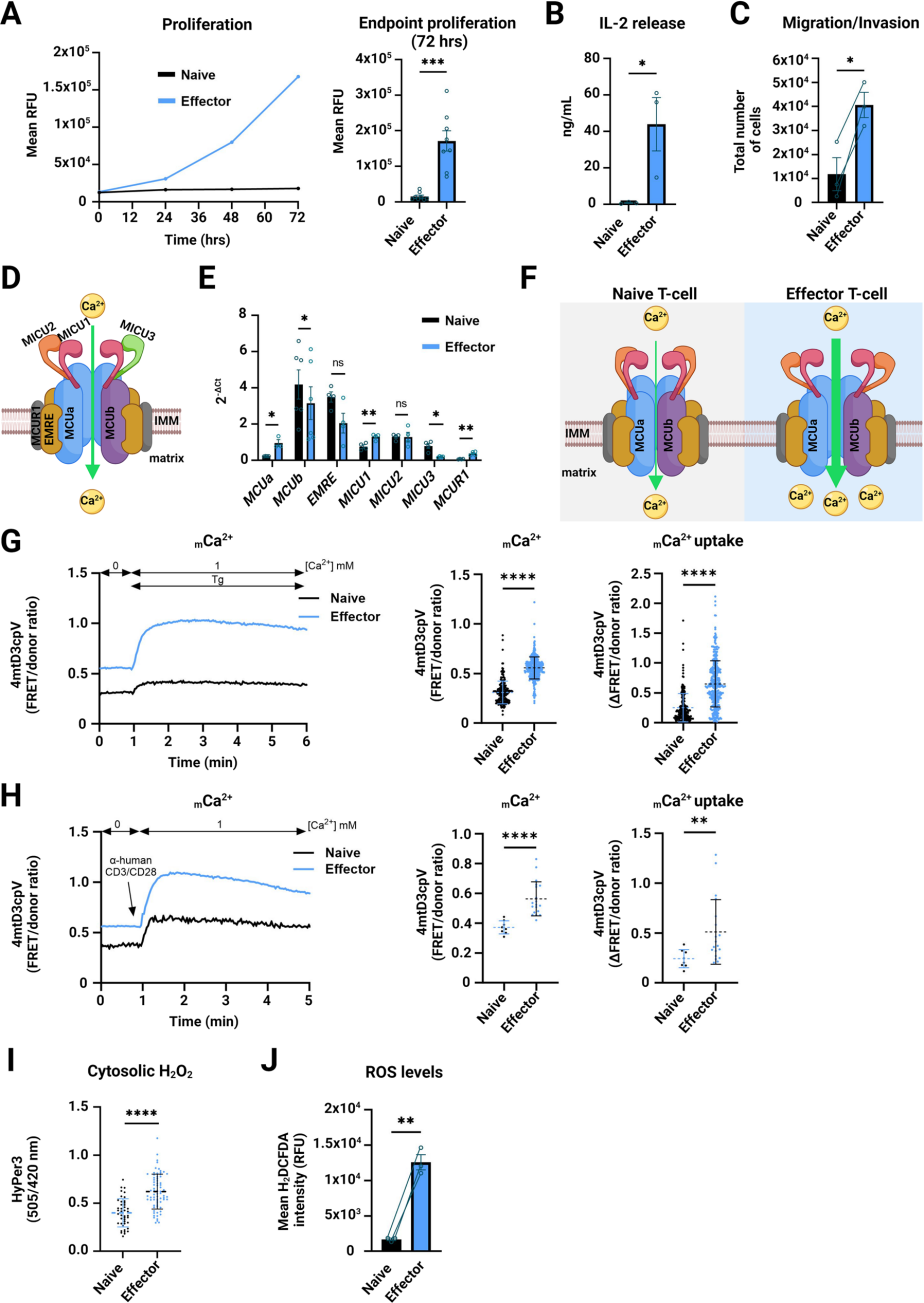
T-cell activation causes elevated _m_Ca^2+^ uptake. (**A**) CD4^+^ T-cell proliferation measured in naive and effector cells. The traces and quantification at 72 hrs represent a mean ± SEM of 8 different donors/biological replicates. ***p < 0.001 (*p* = 0.0006), assessed by two-tailed paired Student’s t-test. RFU = relative fluorescence units. (**B**) IL-2 secretion upon T-cell activation (mean ± SEM of 3 donors/biological replicates shown as single points). *p ≤ 0.05 (*p* = 0.0422), assessed by two-tailed paired Student’s t-test. (**C**) Naive and effector CD4^+^ T-cell trans-well migration/invasion. The quantification shows a mean ± SEM of 3 donors/biological replicates. *p ≤ 0.05 (*p* = 0.0209), assessed by two-tailed paired Student’s t-test. (**D**) Structure of the MCU complex (Image created with BioRender). (**E**) Gene expression analyses of MCU complex components in naive (black bars) and effector CD4^+^ T-cells (blue bars). Data present mean ± SEM of 4–7 donors/biological replicates normalised to TATA-binding protein (*TBP*). **p < 0.01, *p ≤ 0.05, ns - not significant (*MCUa p* = 0.0200; *MCUb p* = 0.0494; *MICU1 p* = 0.0052; *MICU3 p* = 0.0177; *MCUR1 p* = 0.0074); assessed by two-tailed paired Student’s t-test. (**F**) TCR activation leads to higher _m_Ca^2+^ uptake (Image created with BioRender). (**G**) Quantification of _m_Ca^2+^ levels in human naive and effector T-cells by the 4mtD3cpV biosensor. Traces and quantification plots show an average of 224 naive and 371 effector cells/biological replicates from 2 healthy donors. Cells were stimulated by addition of 1 µM thapsigargin at the indicated time point. The quantification/violin plots show mean ± SD basal _m_Ca^2+^ levels and mitochondrial Ca^2+^ uptake. Single cells are given as single points. ****p < 0.0001, assessed by two-tailed unpaired Student’s t-test (Basal *p* = 1.5409E−100; Uptake *p* = 7.67291E−39). (**H**) Quantification of _m_Ca^2+^ levels in human naive and effector T-cells by 4mtD3cpV. Traces and quantification plots show an average of 8 naive and 19 effector cells/biological replicates from 1 donor. Cells were stimulated by addition of anti-human CD3/CD28-coated beads at the indicated time point. The quantification/violin plots show the mean ± SD basal _m_Ca^2+^ levels and mitochondrial Ca^2+^ uptake. ****p < 0.0001 (*p* = 0.000205), **p < 0.01 (*p* = 0.0054), assessed by Mann–Whitney U test. (**I**) Cytosolic H_2_O_2_ levels measured with the HyPer-3 biosensor in naive and effector CD4^+^ T-cells. Quantifications show mean ± SD of 32 naive and 164 effector T-cells/biological replicates from 3 healthy donors. ****p < 0.0001 (*p* = 6.2986E−10), assessed by two-tailed unpaired Student’s t-test. (**J**) ROS levels in effector T-cells determined by H_2_DCFDA. Quantification shows mean ± SEM of 3 different donors/biological replicates. **p < 0.01 (*p* = 0.0092), assessed by two-tailed paired Student’s t-test. RFU = relative fluorescence units. Source data are available online for this figure.

Given that the cytosolic Ca^2+^ concentration (_c_Ca^2+^) is essential for T-cell function, we measured _c_Ca^2+^ in naive and effector T-cells and found that both the resting levels, as well as thapsigargin (Tg)-induced SOCE, were elevated in effector T-cells compared to naive T-cells (Fig. EV1D,E). Moreover, both cellular and mitochondrial volume were increased 72 hrs post-T-cell activation (Fig. EV1F–H). Furthermore, functional parameters such as cell proliferation, IL-2 secretion and migration were elevated in the bead-activated T-cells (Fig. 1A–C).

We next proceeded with evaluating the functional role of the MCU complex (Fig. 1D) in primary human naive and effector T-cells. An RT-qPCR-based evaluation indicated that *MCUa*, *MICU1* and *MCUR1* mRNA levels were upregulated, whereas *MCUb* and *MICU3* were downregulated in effector compared to naive T-cells (Fig. 1E). Immunoblot analyses further confirmed that both MCUa and MICU1 are elevated in effector T-cells stimulated with both beads and antibody solution (Appendix Fig. S2A) on a protein level. Having in mind that both MCUa and MICU1 are _m_Ca^2+^ uptake-promoting subunits, we hypothesised that the _m_Ca^2+^ concentration and uptake are elevated in effector T-cells (Fig. 1F). Measurements of _m_Ca^2+^ in both naive and effector T-cells using two genetically-encoded _m_Ca^2+^ sensors and the Ca^2+^-sensitive fluorescent probe Calcium-Green-5N confirmed this hypothesis. We saw that both resting _m_Ca^2+^ and stimulus-induced _m_Ca^2+^ uptake were strongly increased in effector T-cells treated with Tg (Figs. 1G and EV1I) or anti-CD3/CD28-coated beads (Figs. 1H and EV1J). Furthermore, effector T-cells exhibited increased _m_Ca^2+^ uptake following digitonin permeabilization (Fig. EV1K), further supporting the results obtained with genetically encoded sensors. Increase in _m_Ca^2+^ is linked with elevated production of ROS (Mishina et al, 2012; Sena et al, 2013); hence, we determined cytosolic H_2_O_2_ and global ROS levels in both cell types. The increase in both of these parameters in effector cells, relative to naive T-cells (Fig. 1I,J), indicated a metabolic shift in the effector T-cells and suggested a possible involvement of mitochondria i.e. the MCU complex.

In summary, we established a reliable experimental system that allowed for the evaluation of MCU in human CD4^+^ T-cell physiology. Moreover, we observed a robust increase in the resting as well as the _m_Ca^2+^ uptake in effector versus naive T-cells.

### Oxidative phosphorylation, glycolytic capacity and mitochondrial translation are elevated in effector T-cells

**Figure 2:**
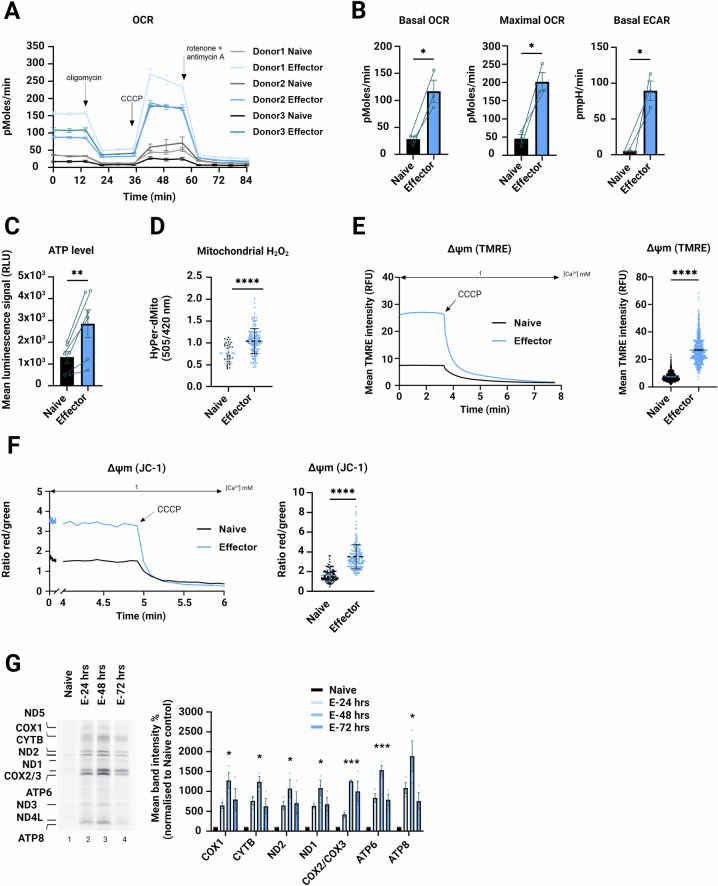
TCR stimulation induces a robust functional reprogramming of T-cell mitochondria. (**A**, **B**) Resting and maximal mitochondrial oxygen consumption rates (OCR), and extracellular acidification rates (ECAR) in effector CD4^+^ T-cells from 3 donors measured using the Seahorse assay. (**B**) Quantified mean ± SEM of 3 healthy donors/biological replicates. *p ≤ 0.05 (Basal *p* = 0.0471; Maximal *p* = 0.0275; ECAR *p* = 0.0274), assessed by two-tailed paired Student’s t-test. (**C**) ATP levels (assessed by the Cell Titer Glo^®^ Luminescence Assay) upon CD4^+^ T-cell activation. Quantification shows mean ± SEM of 6 healthy donors/biological replicates. **p < 0.01 (*p* = 0.0083), assessed by two-tailed paired Student’s t-test. (**D**) Mitochondrial H_2_O_2_ levels in naive and effector CD4^+^ T-cells. Quantifications show a mean ± SD of 43 naive and 71 effector T-cells/biological replicates from 3 healthy donors. ****p < 0.0001 (*p* = 8.9134E−07), assessed by two-tailed unpaired Student’s t-test. (**E**) Single cell measurements of ∆Ψm using TMRE in naive and effector T-cells. Traces show mean RFU of 782 naive and 678 effector T-cells/biological replicates from 4 healthy donors. The quantification on the right shows mean ± SD of the resting ∆Ψm. CCCP: 2 μM. ****p < 0.0001 (*p* = 0), assessed by two-tailed unpaired Student’s t-test. RFU = relative fluorescence units. (**F**) Single cell measurements of ∆Ψm using JC-1 staining. Traces and quantification show mean ± SD of 121 naive and 164 effector T-cells/biological replicates from 2 healthy donors. CCCP: 2 μM. ****p < 0.0001 (*p* = 1.41716E−40), assessed by two-tailed unpaired Student’s t-test. (**G**) Mitochondrial protein translation measured at 0, 24, 48, and 72 hrs after TCR activation, using metabolic ^35^S labelling. The blot is representative of one donor and the quantification is a mean ± SEM of 3 different donors/biological replicates (single points), as percentage of ‘Naive control’. ***p < 0.001; *p ≤ 0.05 (COX1 *p* = 0.0278; CYTB *p* = 0.0138; ND2 *p* = 0.0484; ND1 *p* = 0.0384; COX2/COX3 *p* = 0.0004; ATP6 *p* = 0.0065; ATP8 *p* = 0.0428), assessed by two-tailed paired Student’s t-test. E = Effector. Source data are available online for this figure.

To examine whether the alterations in _m_Ca^2+^ are associated with metabolic changes following T-cell activation, we determined mitochondrial respiration (oxygen consumption rates; OCR) and extracellular acidification rate (ECAR) as a marker of glycolytic activity in naive and effector T-cells from three human donors. Our results demonstrated that both OCR and ECAR were significantly elevated in effector, compared to naive T-cells (Fig. 2A,B). Furthermore, cellular ATP levels (Fig. 2C) as well as mitochondrial H_2_O_2_ levels (Fig. 2D) were also increased. The protonmotive force controls mitochondrial function and ATP production. Given that the mitochondrial membrane potential (ΔΨm) is the major driving force for mitochondrial Ca^2+^ uptake, we performed measurements of ΔΨm in mitochondria of single cells using the indicators TMRE (Fig. 2E) and JC-1 (Fig. 2F). Mitochondria in both cell types readily accumulated the probes, and responded with probe release (depolarization) to the addition of the uncoupler CCCP.

A ^35^S methionine labelling assay revealed that translation of all mitochondria-encoded proteins was increased shortly after T-cell activation (Fig. 2G). As depicted, a clear time-dependence was observed, with the highest increase in translation taking place 48 hrs following T-cell stimulation. The steady-state expression of proteins expressed in different mitochondrial sub-compartments and with diverse functions, including TACO1, NDUFA9, SDHA, ATP5B, TMBIM5, and AFG3L2 was also elevated (Appendix Fig. S2B).

These findings suggest that activation of naive T-cells and their consequent differentiation is followed by a strong increase of mitochondrial biogenesis (as also observed in Fig. EV1F–H) resulting in increased Ca^2+^ storage/transport capacity and ATP synthesizing ability, and in a large stimulation of the glycolytic flux. This set of changes would therefore match the increased demand for both ATP and metabolites that is required for DNA and protein synthesis in these rapidly proliferating effector T-cells.

### Transcriptome and proteome analyses reveal key cellular processes affected by T-cell activation

**Figure 3:**
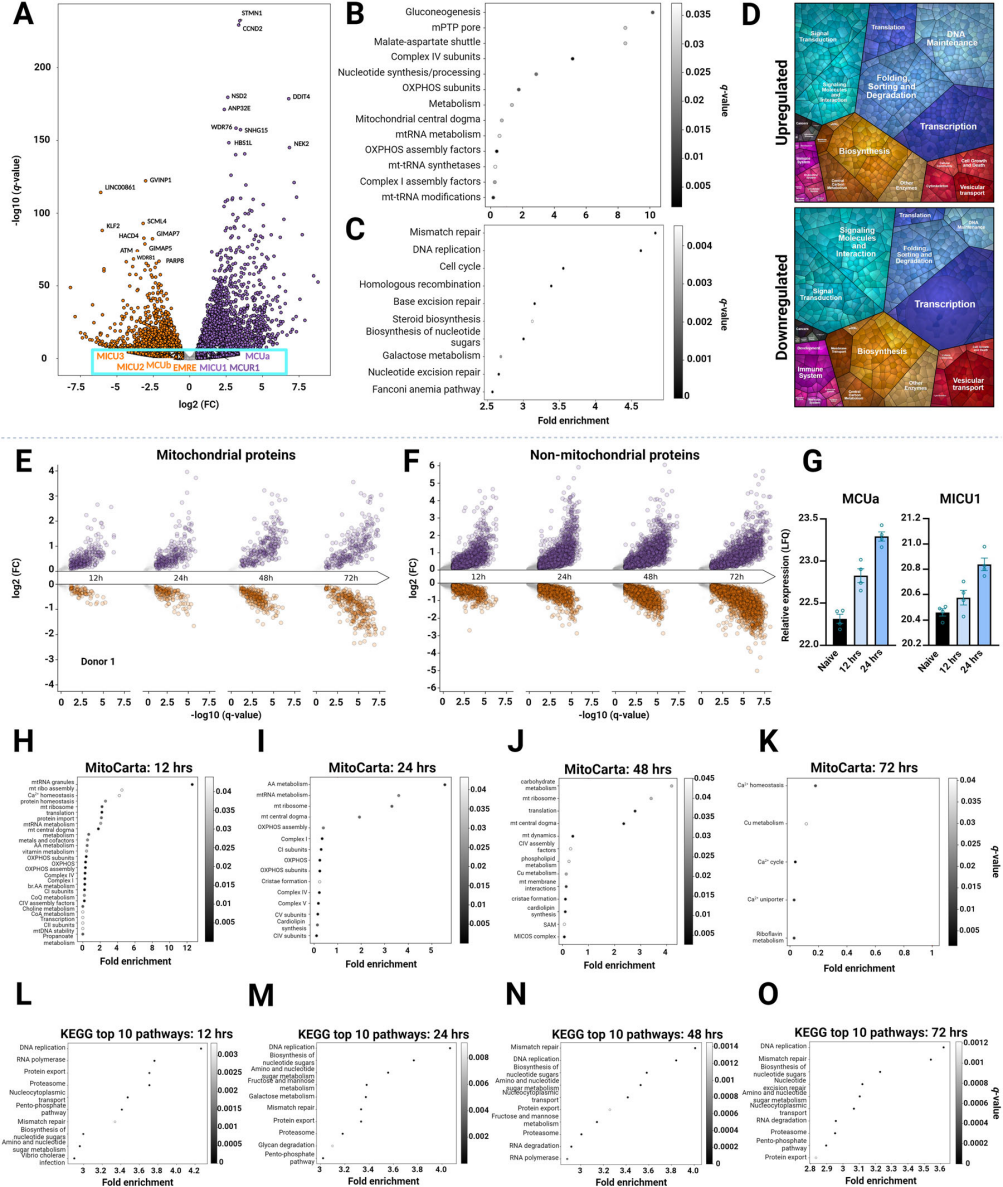
T-cell activation causes time-dependent transcriptome and proteome rewiring. (**A**–**D**) RNA sequencing of naive and effector CD4^+^ T-cells. (**A**) Volcano plot displays DEGs between naive and effector T-cells. DEGs have been selected and sorted as up- or down-regulated depending on the fold change (up—FC > 0 (purple); down—FC < 0 (orange)) using a *q*-value cut-off of 0.05 (DEseq2, FDR-correction). MCU complex components are marked with a blue frame. (**B**) MitoCarta 3.0-based sub-mitochondrial compartment and pathway annotation. *q*-values were calculated using Fisher’s exact test. (**C**) Top 10 pathways (according to KEGG) ranked by fold enrichment, involved in T-cell activation after 72 hrs of stimulation, excluding mitochondria-related genes. (**D**) Proteomap analyses based on data shown in (**A**), excluding mitochondria-related genes. (**E**–**O**) Proteomics and bioinformatic analyses of bead-activated T-cells for 12, 24, 48, and 72 h. The volcano plots (**E**, **F**) show significantly up- (purple) and downregulated (orange) proteins (DEPs) as compared to naive T-cells. (**E**) Mitochondria-related DEPs in effector T-cells as compared to naive T-cells. (**F**) Non-mitochondria-related DEPs in activated T-cells compared to naive T-cells. (**G**) Relative protein abundance of MCUa and MICU1 extracted from the proteomics dataset. Data is shown as mean ± SEM from 4 technical replicates. LFQ = label-free quantitation. (**H**–**K**) MitoCarta 3.0-based sub-mitochondrial compartment and pathway annotation. *q*-values were calculated using Fisher’s exact test. (**L**–**O**) Top 10 pathways (according to KEGG) ranked by fold enrichment, involved in T-cell activation after 12, 24, 48, and 72 hrs of stimulation, excluding mitochondria-related proteins. Source data are available online for this figure.

To gain additional insights into the contribution of mitochondria in the molecular and genetic alterations induced by T-cell activation, we performed RNA sequencing (RNA-seq) of naive and effector T-cells. The bioinformatic evaluation of these data supported our findings regarding the robust T-cell functional alterations and identified differentially-expressed genes (DEGs) in effector versus naive T-cells (Fig. 3A), indicating a well-coordinated T-cell genetic reorganisation following TCR engagement. Notably, the RNA-seq data confirmed the previously observed (refer to Fig. 1E) expressional alterations of the MCU complex: upregulation of *MCUa*, *MICU1* and *MCUR1* and downregulation of *MCUb*, *MICU2 and MICU3* (blue box Fig. 3A). To evaluate the impact of T-cell activation on mitochondrial function, we applied pathway analyses only on DEGs included in the human MitoCarta3.0 (Rath et al, 2021). Pathway analyses identified genes involved in gluconeogenesis, nucleotide processing, oxidative phosphorylation (OXPHOS), and electron transfer chain (ETC) assembly, among others, highly affected by TCR stimulation (Fig. 3B). To evaluate the physiological relevance of the TCR-induced T-cell activation, we determined the affected signalling pathways for all genes, excluding the ones from MitoCarta3.0. (Fig. 3C). Along with the additional proteomap-based investigations of cellular functions affected by T-cell activation, our analyses revealed that cellular biosynthesis, bioenergetics, metabolism, signalling, transcription and protein folding, sorting and degradation are some of the highest affected cellular processes (Fig. 3D).

To assess the time-dependent activation-induced metabolic and transcriptional T-cell reprogramming, we next performed proteome analysis of naive and effector T-cells from two healthy human donors, 12, 24, 48, and 72 hrs following activation. We observed a very distinct protein abundance pattern in both mitochondrial and non-mitochondrial proteomes in the two donors (Figs. 3E,F and EV2A,B). Already 12 hrs after activation, the abundance of proteins was significantly altered compared to the naive T-cells. The number of differentially-expressed proteins (DEPs) continuously increased and reached highest expression levels 72 hrs post-activation. As depicted, many of the DEPs were mitochondrial, a finding that supported our previous results regarding the essential involvement of these organelles in the T-cell activation. Of note, a time-dependent upregulation of MCUa and MICU1 was observed in the proteomic screen (Fig. 3G). The pathway analyses for all time points attested that after 12 hrs of activation, the abundance of proteins involved in mitochondrial transcription, protein homeostasis, ribosome biology, as well as _m_Ca^2+^ signalling was altered (Fig. 3H). Following 24 and 48 hrs of activation, proteins involved in amino acid, mtRNA and carbohydrate metabolism were significantly affected (Fig. 3I,J). Interestingly, 72 hrs after activation, proteins involved in _m_Ca^2+^ were the most affected (Fig. 3K). The complete proteome evaluation, excluding the mitochondrial proteome, revealed a number of significantly affected pathways, including DNA replication, metabolism, biosynthesis, proteasome regulation, and several transport mechanisms, among others (Fig. 3L–O). These findings were further corroborated by evaluation of the DEPs using proteomaps (Fig. EV2C–F).

**Figure EV2:**

Activation of naive human T-cells causes time-dependent proteome alterations. Proteomics and bioinformatic analyses of bead-activated T-cells for 12, 24, 48, and 72 h. The volcano plots (**A**, **B**) show significantly up- (purple) and downregulated (orange) proteins as compared to naive T-cells. (**C**–**F**) Proteomap-based evaluation of signalling pathways and cellular functions regulated by significantly up- (upper panels) and downregulated (lower panels) proteins following bead-induced T-cell activation (from 12 to 72 h). Proteomaps are based on differentially-expressed proteins (DEPs) depicted in Fig. 3F.

In summary, we identified a substantial and time-dependent transcriptome and proteome rewiring following TCR stimulation. Moreover, our bioinformatic data suggest that mitochondria have a key role in this context.

### MCU controls mitochondrial Ca^2+^ dynamics and effector T-cell function

**Figure 4:**
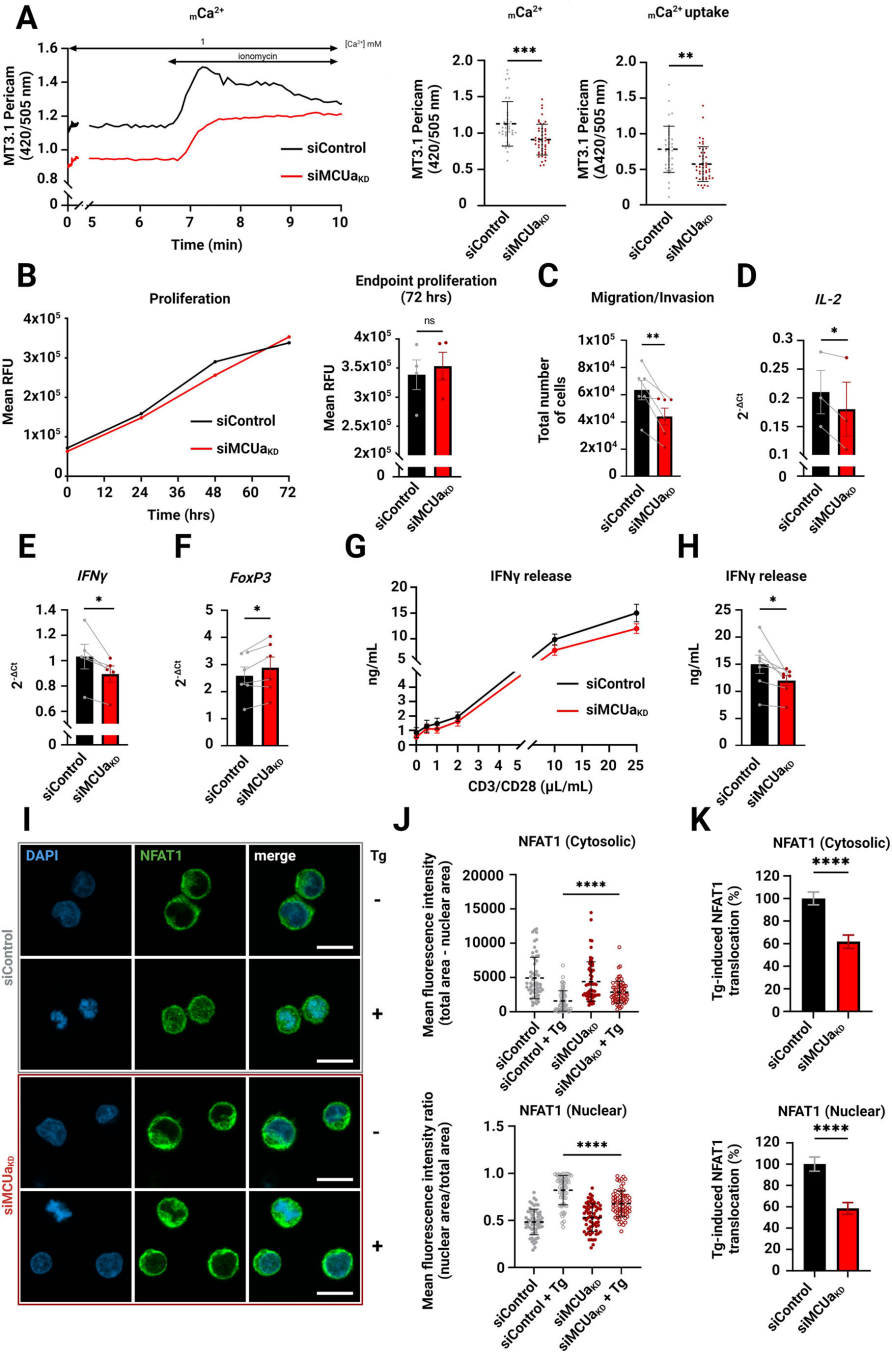
MCUa_KD_ inhibits _m_Ca^2+^ uptake and affects crucial T-cell functional parameters. (**A**) MT3.1 Pericam-based quantification of resting _m_Ca^2+^ and _m_Ca^2+^ uptake upon ionomycin stimulation (4 µM). The traces and violin plots (mean ± SD) show the average of 38 control effector T-cells and 48 siMCUa_KD_ effector T-cells/biological replicates from 4 donors. ***p < 0.001; **p ≤ 0.01 (Basal *p* = 0.0002; Uptake *p* = 0.0011), assessed by two-tailed unpaired Student’s t-test. (**B**) Viability of siMCUa_KD_ T-cells and controls determined by CellTiter-Blue® assay. Traces and graph (72 h) show mean ± SEM of 4 healthy donors/biological replicates. ns - not significant, assessed by two-tailed paired Student’s t-test. RFU = relative fluorescence units. (**C**) Migration/invasion of siMCUa_KD_ cells and controls determined by trans-well assay. Quantification shows mean ± SEM of 6 different donors/biological replicates. **p ≤ 0.01 (*p* = 0.0084), assessed by two-tailed paired Student’s t-test. (**D**) mRNA expression of *IL-2* in siMCUa_KD_ cells and controls determined by RT-qPCR. Quantification shows mean ± SEM of 3 different donors/biological replicates. *p ≤ 0.05 (*p* = 0.0455), assessed by two-tailed paired Student’s t-test. (**E**) mRNA expression of *IFNγ* in siMCUa_KD_ cells and controls determined by RT-qPCR. Quantification shows mean ± SEM of 5 healthy donors/biological replicates. *p ≤ 0.05 (*p* = 0.0352), assessed by two-tailed paired Student’s t-test. (**F**) mRNA expression of Treg transcription factor *FoxP3* in siMCUa_KD_ cells and controls determined by RT-qPCR. Quantification shows mean ± SEM of 6 heathy donors/biological replicates. *p ≤ 0.05 (*p* = 0.0312), assessed by two-tailed paired Student’s t-test. (**G**, **H**) ELISA-based measurements of IFNγ secretion in Ab-stimulated siMCUa_KD_ effector T-cells. Quantification (at 25 µL/mL Ab solution) shows mean ± SEM of 7 healthy donors/biological replicates. *p ≤ 0.05 (*p* = 0.0156), assessed by Wilcoxon’s signed-rank test. (**I**, **J**) Evaluation of endogenous NFAT1 upon T-cell activation with 1 µM Tg in siMCUa_KD_ effector T-cells and control cells. (**I**) Representative confocal images of control-transfected and siMCUa_KD_ cells, with or without Tg stimulation. Left to right: DAPI, NFAT1, merged channels; magnification: 40× (N. A 1.3) oil; scale bar: 10 μm. (**J**) Quantification (upper panel) shows NFAT1 mean ± SD intensity difference between the total cell area and the nuclear area. Quantification (lower panel) shows NFAT1 mean ± SD intensity ratio of the nuclear area of the cell to the total cell area. Both graphs represent a total of 59 control cells+DMSO, 61 control cells+Tg, 65 siMCUa_KD_ cells+DMSO, and 65 siMCUa_KD_ cells+Tg cells/biological replicates from 3 healthy donors. ****p ≤ 0.0001 (Cytosolic *p* = 8.38774E−06; Nuclear *p* = 2.12737E−07), assessed by Welch’s t-test. (**K**) Quantification of % of Tg-induced NFAT1 translocation based on data in (**J**). Both graphs show mean ± SEM of 61 control and 65 siMCUa_KD_ cells. ****p ≤ 0.0001 (*p* = 0.00001), assessed by two-tailed unpaired Student’s t-test. Source data are available online for this figure.

The substantial increase in mitochondrial metabolism and the functional rearrangement of the MCU complex in effector T-cells prompted further investigation of the precise role of MCU and _m_Ca^2+^ in CD4^+^ T-cell function. To that end, we induced transient siRNA-based MCUa knockdown (siMCUa_KD_) in effector T-cells (Fig. EV3A,B). Functional measurements of _m_Ca^2+^ indicated that MCUa_KD_ leads to partial reduction of resting _m_Ca^2+^ levels and diminished _m_Ca^2+^ uptake (Fig. 4A). Control experiments showed that this decrease in _m_Ca^2+^ was not due to unspecific regulation of other genes involved in _m_Ca^2+^ homeostasis, including *Orai*/*STIM* and *NCLX*. Also, the expression of the other MCU components, *MICU1, MCUb* and *EMRE* was not affected by *MCUa* silencing (Fig. EV3C). Interestingly, both resting _c_Ca^2+^ and SOCE were decreased in the siMCUa_KD_ T-cells (Fig. EV3D–H), indicating that MCU is involved in controlling not only mitochondrial but also, to a certain extent, cellular Ca^2+^ homeostasis.

**Figure EV3:**

MCUa_KD_ affects human effector T-cell function. (**A**) RT-qPCR-based evaluation of transient MCUa knockdown efficiency in human CD4^+^ effector T-cells. The quantified graph shows mean ± SEM of 9 healthy donors/biological replicates, as compared to control transfection. ****p < 0.0001 (*p* = 3.89251E−13), assessed by two-tailed paired Student’s t-test. (**B**) Immunoblot-based evaluation of transient MCUa knockdown efficiency in human CD4^+^ effector T-cells. Quantification shows mean ± SEM from 3 healthy donors/biological replicates as compared to control transfection, normalised to Histone H3. **p < 0.01 (*p* = 0.0033), assessed by two-tailed paired Student’s t-test. (**C**) RT-qPCR-based evaluation of Ca^2+^ signalling-related genes and MCU complex components in effector T-cells with transient knockdown of *MCUa*. Quantifications show mean ± SEM from 3 healthy donors/biological replicates for all assessed genes. ns - not significant, assessed by two-tailed paired Student’s t-test. (**D**, **E**) Measurements of _c_Ca^2+^ levels using Fura2 in control and siMCUa_KD_ effector T-cells. Quantification shows mean ± SD of 648 control and 739 siMCUa_KD_ T-cells/biological replicates from 3 healthy donors. Tg = 1 μM. ****p < 0.0001 (Basal *p* = 8.86452E−78; SOCE *p* = 3.26694E−11), assessed by two-tailed unpaired Student’s t-test. (**F**, **G**) Measurements of _c_Ca^2+^ levels using Fura2 in siMCUa_KD_ effector T-cells. Quantification shows mean ± SD of 848 control and 930 siMCUa_KD_ T-cells/biological replicates from 2 healthy donors. Tg = 1 μM. ****p < 0.0001 (*p* = 5.75699E−46), assessed by two-tailed unpaired Student’s t-test. (**H**) Measurements of _c_Ca^2+^ levels using Fura2 in control and siMCUa_KD_ effector T-cells stimulated with anti-human CD3/CD28-coated beads. Quantification shows mean ± SD of 169 control and 365 siMCUa_KD_ T-cells/biological replicates from 2 healthy donors. **p < 0.01 (*p* = 0.0033), assessed by Student’s t-test. (**I**, **J**) *IFNγ* mRNA levels in re-stimulated siMCUa_KD_ T-cells. The quantification (at 25 µL/mL Ab solution) shows mean ± SEM of 8 healthy donors/biological replicates. *p ≤ 0.05 (*p* = 0.0457), assessed by two-tailed paired Wilcoxon test. Source data are available online for this figure.

Next, we explored possible functional consequences of MCUa_KD_ in effector T-cells. We found that T-cell viability/proliferation was not significantly affected (Fig. 4B). On the other hand, chemokine-induced in vitro T-cell migration was decreased (Fig. 4C). To understand the molecular mechanisms linking MCU activity and T-cell migration, we performed cytokine expression and release screens. As shown in Fig. 4D,E, the expression of the essential proinflammatory cytokines *IL-2* and *IFNγ* was reduced in siMCUa_KD_ human effector T-cells. Moreover, the expression of the anti-inflammatory Treg transcription factor *FoxP3* was elevated (Fig. 4F), indicating a potentially disturbed proinflammatory function of siMCUa_KD_ T-cells. To evaluate the relationship between stimulus strength and cytokine secretion rate, as well as the impact of the secondary TCR engagement, we treated the effector T-cells with a range of anti-CD3/CD28 antibody concentrations and evaluated IFNγ secretion and mRNA levels. The ELISA-based measurements demonstrated that IFNγ release is dependent on the strength of the stimulus i.e. the anti-CD3/CD28 concentration (Fig. 4G). More importantly, these measurements confirmed the involvement of MCU in T-cell cytokine secretion, as the production of IFNγ after cell stimulation with 25 µL/mL anti-CD3/CD28 was diminished in siMCUa_KD_ effector T-cells (Fig. 4H). Similar results were obtained by measuring *IFNγ* mRNA levels (Fig. EV3I,J).

T-cell cytokine expression is regulated by Ca^2+^-sensitive transcription factors (TFs) such as the nuclear factor of activated T-cells (NFAT) (Hogan et al, 2003). The NFAT-Ca^2+^-mitochondria interplay was recently shown to be important for T-cell function (Vaeth et al, 2017). Accordingly, we inquired whether MCUa_KD_ affects NFAT activity. As depicted, Tg-induced NFAT1 nuclear translocation was diminished in siMCUa_KD_ effector T-cells compared with control siRNA-transfected cells (Fig. 4I–K). These effects of MCU on NFAT activity, and thereby cytokine expression, provide additional evidence about the functional importance of _m_Ca^2+^ homeostasis in T-cell function.

Taken altogether, we observed that transient acute MCUa downregulation affects essential parameters of T-cell function, including IFNγ secretion and in vitro migration and invasion.

### MCU affects vital mitochondrial T-cell parameters

**Figure 5:**
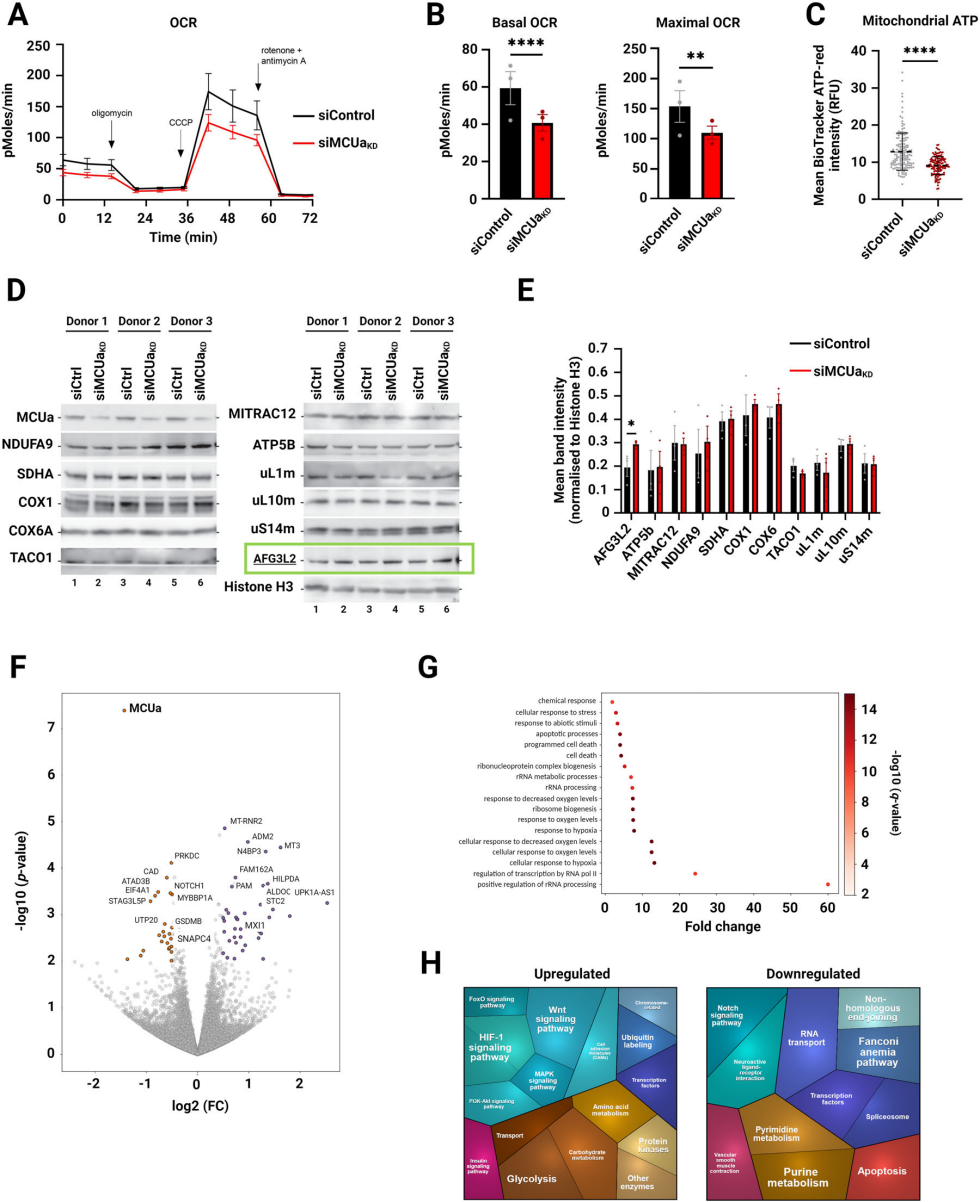
MCUa_KD_ affects vital mitochondrial parameters. (**A**) Oxygen consumption rate measurements (OCR) in control and siMCUa_KD_ effector CD4^+^ T-cells from 3 healthy donors/biological replicates given as mean ± SEM. (**B**) Quantification from the 3 healthy donors/biological replicates in (**A**) as mean ± SEM. ****p < 0.0001; **p < 0.01 (Basal *p* = 3.8776E−07; Maximal *p* = 0.0038), assessed by two-tailed unpaired Student’s t-test. (**C**) Mitochondrial ATP levels, measured using BioTracker ATP-Red dye in control and siMCUa_KD_ T-cells. Violin plots display mean ± SD of 155 control and 130 siMCUa_KD_ T-cells/biological replicates from 2 healthy donors. ****p < 0.0001 (*p* = 1.8025E−13), assessed by two-tailed unpaired Student’s t-test. RFU = relative fluorescence units. (**D**) Mitochondrial protein abundance in control and siMCUa_KD_ cells from 3 healthy donors/biological replicates (**E**) Quantification of abundance shown in (**D**) normalised to Histone H3. Data are shown as mean ± SEM. *p ≤ 0.05 (*p* = 0.0403), assessed by two-tailed paired Student’s t-test. (**F**–**H**) RNA sequencing of control and siMCUa_KD_ cells. (**F**) Volcano plot. DEGs were selected and sorted as up- or down-regulated depending on the fold change (up—FC > 0 (purple); down—FC < 0 (orange)) using a *p*-value cut-off of 0.05 (DEseq2, FDR-correction). (**G**) KEGG pathway analysis of significantly affected cellular pathways. (**H**) Proteomap-based analysis of data shown in (**F**). Source data are available online for this figure.

Given the impact of MCU on _m_Ca^2+^ uptake and function of effector T-cells, we next investigated the possible involvement of mitochondria and their metabolic output. We thus utilised siMCUa_KD_ T-cells to quantify several important mitochondrial metabolic parameters. As demonstrated in Fig. 5A–C, both resting and maximal OCR, as well as the mitochondrial ATP levels were reduced in the siMCUa_KD_ T-cells compared to control T-cells. Changes in mitochondrial protein translation (Appendix Fig. S3A,B), as well as in complex I and IV activity (Appendix Fig. S3C,D), were not detected.

Steady-state protein expression of several oxidative-phosphorylation-related proteins, ribosomes and enzymes revealed an upregulation of the mitochondrial-AAA protease, AFG3-like protein 2 (AFG3L2) (Fig. 5D, green box, and 5E), but no noticeable changes in expression of other proteins. AFG3L2 regulates MCU assembly and _m_Ca^2+^ homeostasis by degrading non-assembled EMRE proteins and ensuring balanced MCU regulation. In the absence of the AFG3L2, excess EMRE can bind to MCUa independently of MICU proteins, resulting in the accumulation of constitutively open MCU/EMRE complexes and _m_Ca^2+^ overload (König et al, 2016). Since MCUa has been downregulated in our model, the fraction of non-assembled EMRE was likely elevated; thus, a compensatory upregulation of AFG3L2 was expected. Of note, upregulation of AFG3L2 was only seen on protein level, and not on mRNA level (Appendix Fig. S3E). To investigate the role of AFG3L2 in T-cells, we generated siAFG3L2_KD_ T-cells using siRNA editing (Appendix Fig. S3F). Functional measurements of _m_Ca^2+^ showed that siAFG3L2_KD_ led to increased levels of resting _m_Ca^2+^ (Appendix Fig. S3G), as expected. However, the remaining enzymatic activity of AFG3L2 was most likely sufficient to prevent any significant changes in OCR (Appendix Fig. S3H,I).

To further unravel the role of MCU in CD4^+^ T-cells, we performed RNA-seq in control and siMCUa_KD_ human effector T-cells. Bioinformatic analyses indicated that expression of a number of genes is affected by MCUa downregulation (Fig. 5F). Pathway analyses identified multiple cellular pathways, including but not limited to rRNA metabolism and ribosome biogenesis, oxygen biology, responses to hypoxia, transcriptional regulation by RNA polymerase II, and cell death to be under control of MCUa and thereby _m_Ca^2+^ signalling in effector T-cells (Fig. 5G). In addition, the proteomap-based analysis suggested that siMCUa_KD_ leads to suppression of Notch signalling, apoptosis, and purine and pyrimidine metabolism, on one hand, and elevation of Wnt signalling, hypoxia signalling, PI3K-Akt pathway, glycolysis, and MAPK signalling on the other hand (Fig. 5H).

Altogether, using various experimental approaches, we identified metabolic processes and signalling pathways that are controlled by MCU in effector T-cells. These findings further suggest that MCU is a potential regulator of not only T-cell physiology but also T-cell-mediated pathologies.

### MCUa controls CD4^+^ effector T-cells-mediated autoimmune responses in vivo

**Figure 6:**
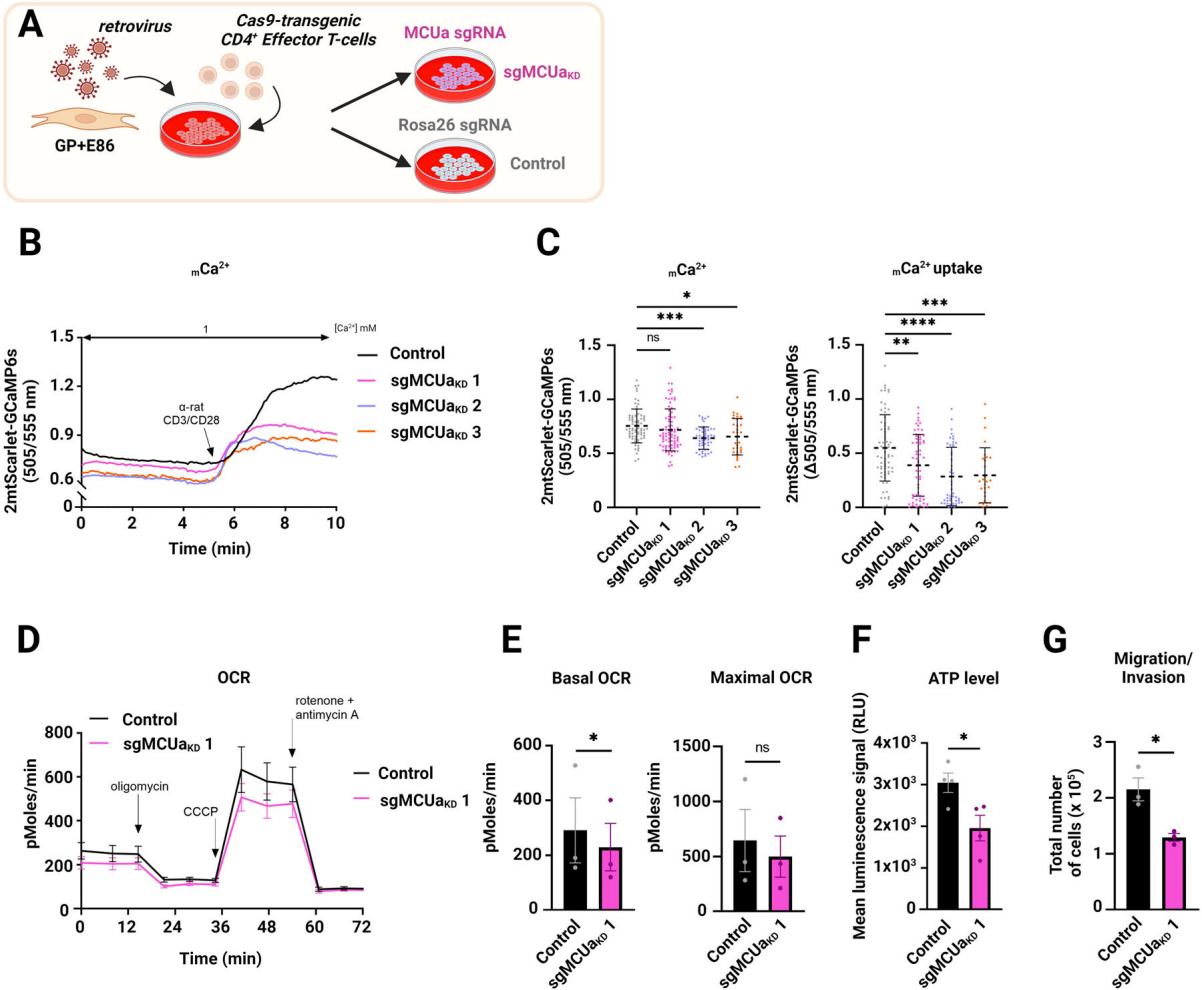
Knockdown of MCUa in rat CD4^+^ effector T-cells affects their function in vitro. (**A**) Generation of stable rat CD4^+^ effector T-cell lines by CRISPR Cas9 and retroviral transduction to target MCUa expression (Image created with BioRender). (**B**) Measurements of _m_Ca^2+^ using 2mtScarlet-GCaMP6s in sgRosa26 control and three sgMCUa_KD_ rat CD4^+^ T-cell clones. TCR stimulation was achieved by addition of anti-rat CD3/CD28 monoclonal antibodies (1 µg/mL). (**C**) Quantification of data presented in (**B**). Violin plots represent mean ± SD of 72 sgRosa26 control, 82 sgMCUa_KD_ 1, 51 sgMCUa_KD_ 2, and 32 sgMCUa_KD_ 3 cells/biological replicates. ****p < 0.0001; ***p < 0.001; **p < 0.01; *p ≤ 0.05; ns - not significant, assessed by ordinary one-way ANOVA (ANOVA summary: Basal *p* = 0.0006; Uptake *p* = 5.55055E−06). (**D**) Seahorse-based measurements of OCR in sgRosa26 control and sgMCUa_KD_ 1 rat CD4^+^ T-cells given as mean ± SD of 3 biological replicates. (**E**) Quantification of data given in (**D**). Basal and maximal OCR given as mean ± SD of 3 biological replicates. *p ≤ 0.05 (*p* = 0.0484); ns - not significant, assessed by Mann–Whitney U test. (**F**) ATP measurements in sgRosa26 control and sgMCUa_KD_ 1 rat CD4^+^ T-cells. Quantification as mean ± SEM of 4 biological replicates. *p ≤ 0.05 (*p* = 0.0307), assessed by two-tailed unpaired Student’s t-test. (**G**) Measurements of in vitro T-cell migration/invasion in sgRosa26 control and sgMCUa_KD_ 1 rat CD4^+^ T-cells. Quantification as mean ± SEM of 3 biological replicates. *p ≤ 0.05 (*p* = 0.0166), assessed by two-tailed unpaired Student’s test. Source data are available online for this figure.

**Figure EV4:**
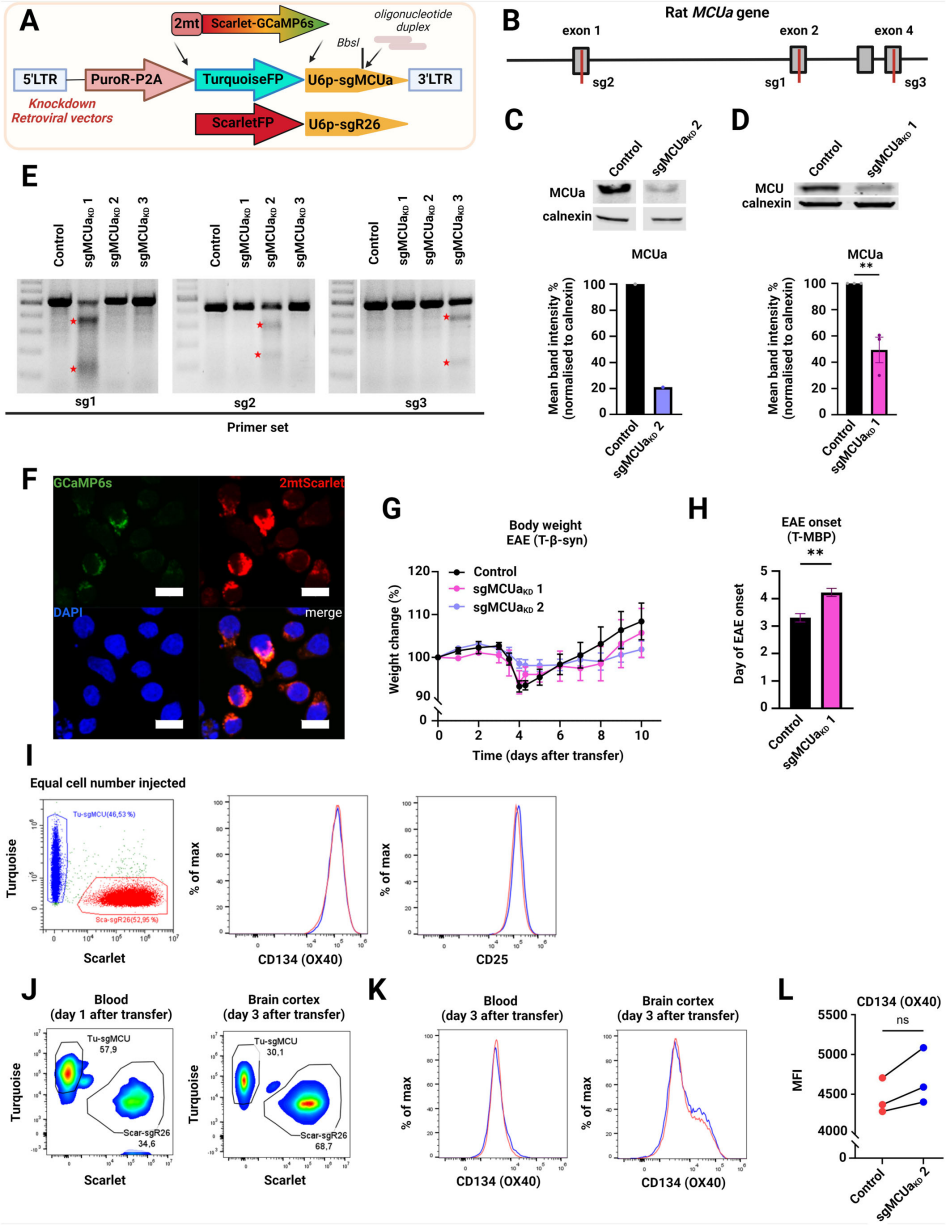
Establishment of MCUa_KD_ models in rat effector T-cells. (**A**) Schematic representation of retroviral constructs used to establish control and sgMCUa_KD_ rat effector T-cell lines. LTR – long terminal repeats; PuroR – puromycin resistance gene; U6p – U6 promoter; 2mt – tandem mitochondrial localisation signals. (**B**) Schematic representation of the rat *MCUa* gene and the position of protospacers targeted by single guide RNAs (sg; the numbering of exons starts from the first coding exon) (Images (**A**) and (**B**) created with BioRender). (**C**, **D**) Immunoblot-based evaluation of knockdown efficiency in sgMCUa_KD_ 2 (**C**) and sgMCUa_KD_ 1 (**D**) cell lines. Protein expression data are normalised to the loading control calnexin. Quantification shows mean ± SEM of 3 experiments/biological replicates. **p < 0.01 (*p* = 0.0065), assessed by two-tailed unpaired Student’s t-test, in (**D**). (**E**) PCR-based T7 endonuclease cleavage confirmed the intended editing of the respective sequence in the different sgMCUa_KD_ cell lines. T7 cleavage fragments, marked by a star, revealed sequence alterations (indels) introduced by Cas9 at a genomic level. (**F**) Confocal images of DAPI-stained sgMCUa_KD_ 1 T-cells transduced by Puro2A-2mtScarlet-GCaMP6s retrovirus show proper mitochondrial localisation of the biosensor 2mtScarlet-GCaMP6s. Scale bar: 10 µm. (**G**) Body weight change curves in animals during transfer EAE experiments (related to Fig. 6H). Data show mean ± SEM, as percentage of initial weight on day 0 (*n* = 4 animals for the control group and *n* = 3 animals for the sgMCUa_KD_ 1 and 2 groups). (**H**) Inactivation of MCUa results in a delayed EAE onset in rats injected with MBP-specific effector T-cells. Data are represented as mean ± SEM of 3 animals/biological replicates per condition. **p < 0.01 (*p* = 0.0018), assessed by Mann–Whitney U test. (**I**) (related to Fig. 7). An equal number of different fluorescently-labelled control (Scarlet fluorescent protein) and sgMCUa_KD_ (Turquoise fluorescent protein) T-cells were co-transferred in Lewis rats. Both cell lines that were injected showed a similar activation state assessed by CD134 (OX-40) and CD25 expression using flow cytometry. (**J**) Ex vivo-isolated T-cells were analysed at different time points after co-transfer using flow cytometry. At day 1 after co-transfer, sgMCUa_KD_ T-cells (57.9%) outnumbered the control cells (34.6%) in the blood. At day 3 after co-transfer, the control T-cells (68.7%) outnumbered the sgMCUa_KD_ cells (30.1%) in the brain. (**K**) sgMCUa_KD_ T-cells that have succeeded to enter the CNS show comparable activation as those cells in the blood, determined by CD134 (OX-40) expression. (**L**) OX-40 expression of control and sgMCUa_KD_ T-cells in the brain at day 3 after co-transfer. Quantification shows mean fluorescence intensity (MFI) of CD134 from 3 rats/biological replicates. ns - not significant, assessed by unpaired Student’s t-test. Source data are available online for this figure.

To evaluate the role of MCU in T-cell biology in vivo, we manipulated MCUa expression in primary rat effector T-cells by a CRISPR Cas9 gene editing approach and analysed their phenotype in an experimental autoimmune encephalomyelitis (EAE) model. To this end, we initially retrovirally engineered sgMCUa_KD_ T-cell lines targeting three different exons of the rat *MCUa* gene, and one control line targeting the rat *Rosa26* genomic locus. Along with specific guide RNAs (sg), we either introduced a fluorescent marker protein (mTurquoise or mScarlet), or the Scarlet-GCaMP6s Ca^2+^ fusion sensor targeted to the mitochondrial matrix (Figs. 6A and EV4A–F). To test functional alterations in these cells, we measured _m_Ca^2+^ using the integrated 2mtScarlet-GCaMP6s sensor in all three sgMCUa_KD_ T-cell lines and the control cell line. Data quantification demonstrated that both resting, as well as TCR-stimulated _m_Ca^2+^ uptake, were reduced in the sgMCUa_KD_ cells (Fig. 6B,C). Furthermore, mitochondrial respiration (Fig. 6D,E), ATP production (Fig. 6F) and migration assessed by a trans-well assay (Fig. 6G) were suppressed in the rat sgMCUa_KD_ T-cells, thus confirming our results obtained with human T-cells. In addition, we overexpressed MCUa in rat and in human T-cells. As expected, the MCUa upregulation induced elevated _m_Ca^2+^ in both cell types. However, the strongly elevated resting _m_Ca^2+^ led to high cell toxicity in the first 24 hrs following transfection, thus preventing further functional evaluation of these cells (Appendix Fig. S4A–G).

**Figure 7:**
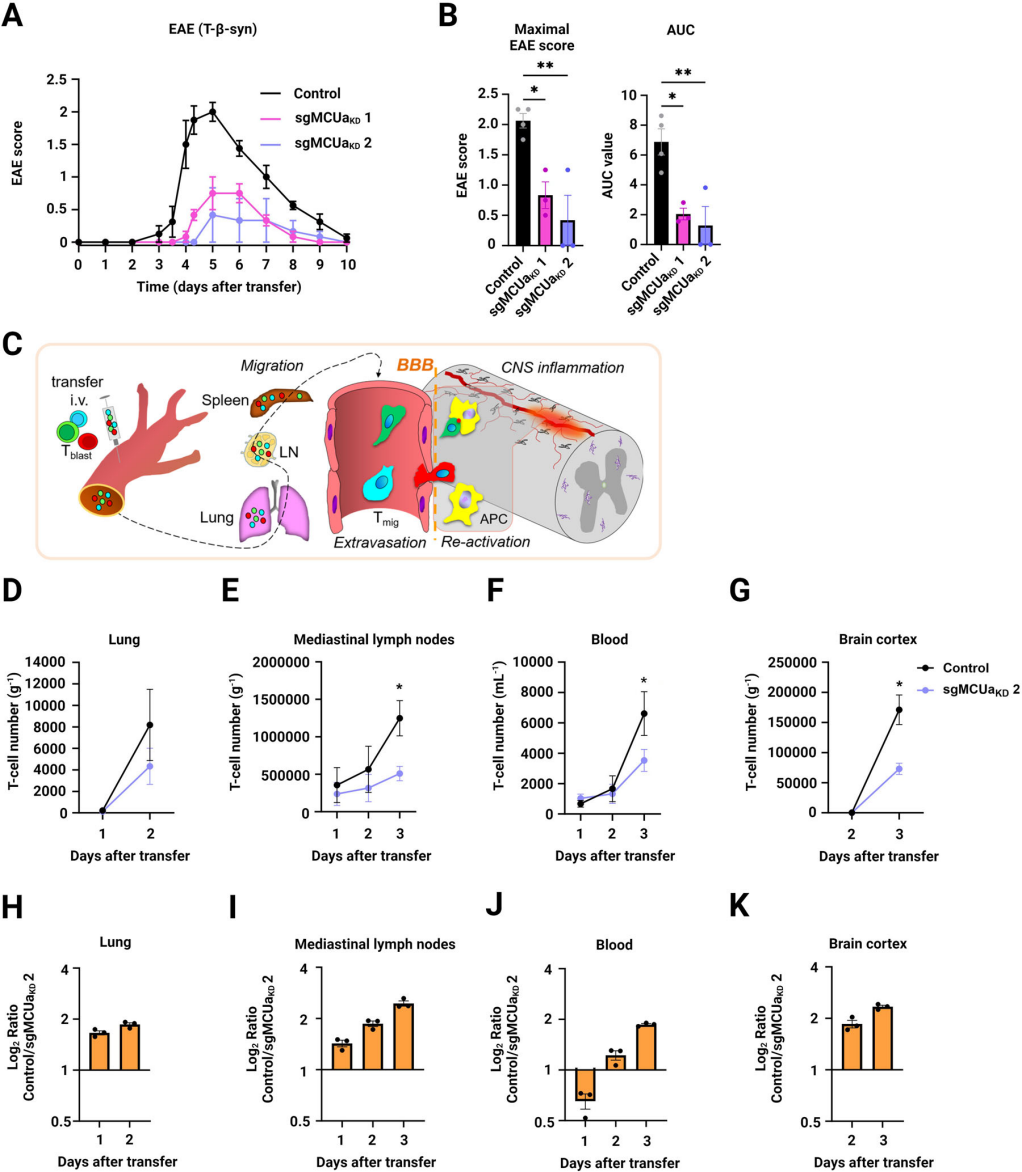
Effector T-cells lacking MCUa have a decreased autoimmune potential in vivo. (**A**) Clinical course of EAE induced by transfer of β-synuclein-specific sgRosa26 control and sgMCUa_KD_ 1 and 2 rat effector CD4^+^ T-cells. Data are shown as mean ± SEM disease scores (*n* = 4 animals for the control group and *n* = 3 animals for the sgMCUa_KD_ 1 and 2 groups). (**B**) Quantification of data shown in (**A**) (*n* = 4 animals for the control group and *n* = 3 animals for the sgMCUa_KD_ 1 and 2 groups). Maximal EAE score and disease severity (presented as area under the curve; AUC). Data are represented as mean ± SEM. **p < 0.01; *p ≤ 0.05, assessed by ordinary one-way ANOVA test (ANOVA summary: Maximal EAE score *p* = 0.0053; AUC *p* = 0.0058). (**C**) T-cell migration pattern after adoptive transfer, prior to induction of clinical EAE. (**D**–**G**) T-cell numbers quantified ex vivo after adoptive T-cell co-transfer in different compartments: (**D**) lungs, (**E**) mediastinal lymph nodes, (**F**) blood, and (**G**) brain cortex. T-cell counts were analysed on a CytoFLEX flow cytometer after staining with anti-CD134 (OX-40)-AF647 and anti-CD25-BV750. Graphs show mean ± SEM (*n* = 3 animals presented as single points) of cell numbers at a given day after transfer. *p ≤ 0.05 (*p* = 0.0351 for **E**, *p* = 0.0500 for **F**, *p* = *p* = 0.0233 for **G**), assessed by two-tailed paired Student’s test. (**H**–**K**) Log_2_ Ratio of control and sgMCUa_KD_ T-cells migrating in the different compartments, given as mean ± SEM (*n* = 3 animals presented as single points; based on data in **D**–**G**) at a certain day after co-transfer. Source data are available online for this figure.

Based on these findings, we proceeded in evaluating the role of MCU in T-cell-mediated autoimmunity. We induced EAE in Lewis rats by adoptive transfer of β synuclein-reactive T-cells (T_β-syn_ cells) (Lodygin et al, 2019) proficient or deficient of MCUa. Notably, disease severity was substantially reduced in animals injected with sgMCUa_KD_ T_β-syn_ cells compared to control T_β-syn_ cells (Fig. 7A,B), with no significant effect on animal body weight (Fig. EV4G). The ameliorating clinical effect upon injection of the two T_β-syn_ cell lines sgMCUa_KD_ 1 and sgMCUa_KD_ 2 was in accordance with the observed impact on _m_Ca^2+^ (refer to Fig. 6B,C). A similar but less pronounced delay in EAE onset was seen upon transfer of myelin-basic protein (MBP)-reactive T-cells (Fig. EV4H).

In the Lewis rat transfer EAE model, injected T-cells initially home to the lung and lung draining lymph nodes, where they acquire migratory properties allowing them to pass the blood-brain barrier and reach the central nervous system (CNS) (Fig. 7C). To assess the effect of MCUa inactivation on effector T-cell migration at a preclinical phase and at disease onset, we co-transferred an equal number of different fluorescently-labelled control (mScarlet FP) and sgMCUa_KD_ (mTurquoise FP) T-cells (Fig. EV4I). Indeed, we observed that fewer sgMCUa_KD_ T-cells, comparing to control cells, were found in different body compartments, including lungs, lymph nodes, blood and brain cortex (Fig. 7D–K). Based on these results we cannot fully exclude that a compromised survival of MCUa-deficient T-cells may partially be responsible for the diminished disease severity. However, on day 1 after the co-transfer, sgMCUa_KD_ T-cells outnumbered control T-cells circulating in the blood (Figs. 7F,J and EV4J), whereas on day 2 after the co-transfer, we found more control T-cells in the brain cortex and nearly equal numbers in the blood (Fig. 7K). This indicated that MCUa-deficient T-cells that enter the lung, penetrate the blood-brain barrier and the CNS less efficiently. Notably, sgMCUa_KD_ T-cells that have succeeded to enter the CNS, albeit being present in diminished numbers relative to control cells, show comparable activation as determined by CD134 (OX-40) expression (Fig. EV4K,L). Therefore, MCU downregulation not only negatively affects the fitness of effector T-cells but also compromises their ability to invade target organs in transfer EAE models.

**Figure EV5:**
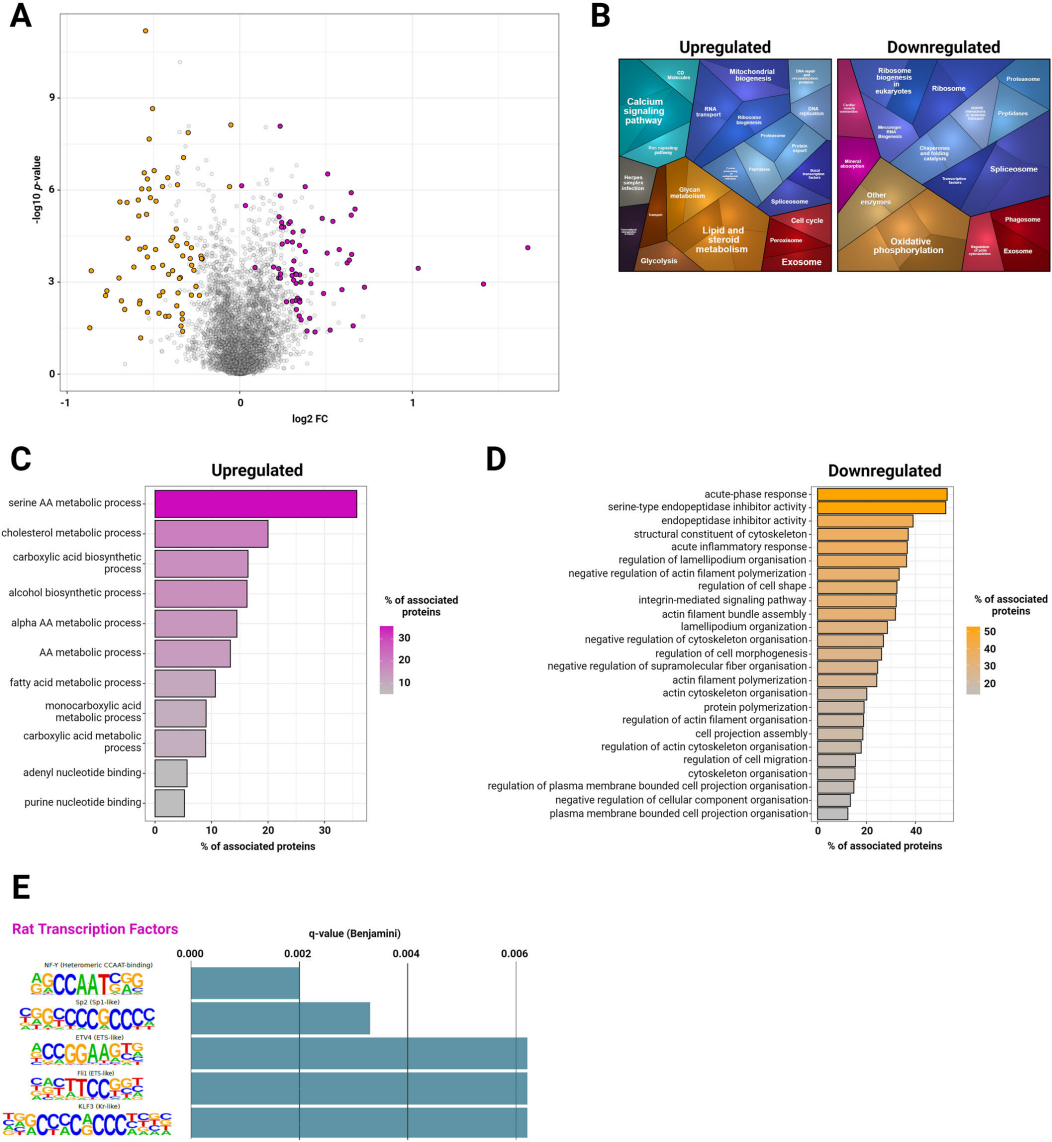
MCUa_KD_ in rat effector T-cells alters their proteome. Proteomics data generated from the two sgMCUa_KD_ cell lines and the control rat T-cell line. (**A**) The volcano plot shows multiple significant up- (magenta) and downregulated (orange) proteins in the two sgMCUa_KD_ cell lines, compared to control, analysed by ANOVA (5% FDR). (**B**) Proteomaps showing the quantitative composition of proteomes with a high probability to be significantly up- (left panel) and downregulated (right panel) upon MCUa knockdown in rat effector T-cells. Proteomaps are based on DEPs shown in (**A**). (**C**, **D**) Significant up- (**C**) and downregulated (**D**) cellular functions and signalling pathways in sgMCUa_KD_ cells ordered by percentage of associated proteins, based on DEPs shown in (**A**). (**E**) HOMER analysis of the proteomic data showing the top five most enriched motifs (TFs). Analysis is based on *q*-values (Benjamini-Hochberg-C).

To better understand the underlying mechanisms responsible for the robust impact of MCU on in vivo T-cell migration/invasion and therefore EAE severity, we performed proteomic analyses of the control and rat sgMCUa_KD_ cell lines 1 and 2 (Fig. EV5A). As depicted in Fig. EV5B–D, similarly to the human T-cell dataset, sgMCUa_KD_ affected the abundance of proteins involved in metabolic regulation. Moreover, we observed downregulation of many proteins involved in T-cell migration and invasion (Fig. EV5D). These findings provided additional cues regarding the role of MCU and validated that mitochondrial metabolic control of T-cell mobility might play a central role in T-cell-mediated autoimmune diseases, strengthening our experimental findings. Hypergeometric optimization of motif enrichment (HOMER) analyses identified several TFs affected by MCUa downregulation, thus providing additional information about regulatory mechanisms that link MCU and T-cell-induced autoimmunity (Fig. EV5E). The exact role of these TFs in T-cells bids further investigation.

In summary, in vivo evaluation of the role of MCU in CD4^+^ T-cells indicated that _m_Ca^2+^ is a crucial determinant of mitochondrial metabolism and T-cell migration and invasion. Moreover, our findings suggest a central role of the MCU complex in T-cell-mediated autoimmune responses.

## Discussion

Engagement of T-cell receptors at the surface of naive CD4^+^ T-cells and the subsequent formation of an immune synapse are central events required for proper T-cell function. Many signalling pathways are initiated to precisely control the complex transformation from a quiescent, naive T-cell phenotype, to a highly active phenotype that characterises a fully developed effector T-cell. Accordingly, this T-cell phenotype switch is accompanied by changes of transcriptional and proteomic profiles, as well as a robust metabolic reprogramming, alterations in biosynthesis and mitochondrial biogenesis (Pearce et al, 2013). The central role of mitochondria in T-cell activation has been demonstrated by studies that found that depletion of essential mitochondrial proteins such as SHMT, a serine hydroxymethyltransferase, the mitochondrial enzyme COX10, or mitochondrial transcription factor A (TFAM), causes altered T-cell biology (Desdín-Micó et al, 2020; Ron-Harel et al, 2016; Tan et al, 2017).

Mitochondrial function is highly dependent on the Ca^2+^ dynamics within the matrix, but also within the intermembrane space (Petrungaro et al, 2015). For example, several mitochondrial dehydrogenases are activated by elevation of matrix Ca^2+^ levels (Denton, 2009) and different IMM transporters appear to have Ca^2+^-binding motifs, indicating that mitochondrial metabolite exchange is also a Ca^2+^-controlled process.

The major molecular components of the MCU complex were identified more than a decade ago and, since, a number of studies have been focused on addressing the structural insights and the functional relevance of MCU in different experimental models (Foskett and Philipson, 2015; Mammucari et al, 2018; Nemani et al, 2018; Shanmughapriya et al, 2015). Most of these studies report that MCUa, MCUb, MICU1-2, and EMRE have important functional relevance in immunity, muscle cell physiology, as well as pathological conditions such as cancer, cardiovascular and neurodegenerative diseases, among others (Kwong et al, 2015; Marchi et al, 2019; O’Rourke et al, 2021; Patron et al, 2018; Qin et al, 2023; Seegren et al, 2020; Stejerean-Todoran et al, 2022; Tosatto et al, 2016; Vultur et al, 2018; Walters and Usachev, 2023). Despite the involvement of mitochondria in T-cell activation and differentiation, along with the well-established role of Ca^2+^ in mitochondrial function, the contribution of MCU in CD4^+^ T-cell biology is not fully understood thus far.

Two recent studies explored the role of MCU in mouse CD4^+^ T-cells and Jurkat T-cells. Yoast et al focused on examining the impact of MCU on SOCE in both cell types, but did not evaluate the direct influence of MCU on metabolic and functional T-cell parameters such as proliferation, cytokine secretion or migration (Yoast et al, 2021). On the other hand, using *Mcu*^fl/fl^*Cd4*^Cre^ mice, Wu et al analysed the functional impact of MCU in T helper cells in vivo (Wu et al, 2021). Unexpectedly, despite the absence of MCUa in the CD4^+^ T-cells, no significant alterations in several functional parameters could be observed. Moreover, mitochondrial OCR was not affected, and only a small effect on SOCE could be observed. Such unexpected observations are not novel in the MCU research field and it is becoming increasingly apparent that complete and chronic deletion of the *Mcu* gene in vivo is followed by yet-unidentified compensation mechanism(s) that seem to render the targeted cells and organ systems less dependent on MCU-controlled _m_Ca^2+^ uptake (Murphy et al, 2014). Thus, a more complex experimental set-up was necessary to understand the role of MCU in T-cells. Hence, in this study, we applied different experimental techniques to acutely suppress MCUa expression and prevent the development of compensatory mechanisms. To this end, a recent study compared the effects of chronic versus acute deletion of MCU in regulatory T-cells (Tregs) and concluded that development of compensatory mechanisms is a plausible explanation for the lack of functional alterations in the *Mcu*^fl/fl^*Cd4*^Cre^ mice (Jost et al, 2023). Furthermore, the authors found that downregulation of MCU impairs the suppressive capacity of Tregs, which can influence EAE. However, we can largely rule out the involvement of Tregs in our experimental system, as the Cas9-mediated MCUa knockdown in our study occurs exclusively in fully differentiated effector T-cells, unlike the global T-cell knockdown seen in the mouse model. Based on previous studies and sequencing data from this work, it is clear that the rat effector T-cells in our model do not differentiate into Tregs. Therefore, we are confident that the observed effects of MCUa knockdown are solely mediated by encephalitogenic Th1/Th17 effector T-cells.

The present study provides novel insights into the role of MCU-mediated Ca^2+^ uptake in CD4^+^ T-cell metabolism and function. We found that *MCUa*, *MICU1* and *MCUR1* expression is increased in effector T-cells, while *MCUb* is decreased. Analyses of already published proteome datasets from mouse CD4^+^ T-cells supported our findings, as MCUa was upregulated 16- and 24 hrs after stimulation. Furthermore, both studies reported MCUb downregulation, while MICU1 was upregulated 24 hrs following activation (refer to Appendix Fig. S5, and (Howden et al, 2019; Ron-Harel et al, 2016)).

The functional evaluation of the findings based on _m_Ca^2+^ measurements in naive and effector T-cells further supported the hypothesis that activation processes are linked with an increased Ca^2+^ uptake capacity. Our findings indicate that the mitochondrial Ca^2+^ influx and signalling are essential events required to support the robust metabolic transformation following T-cell activation. The increased capacity for Ca^2+^ influx might thus contribute to a more efficient exploitation of the mitochondrial system for ATP replenishment and biomolecule synthesis. Based on our data, we propose that this elevated mitochondrial Ca^2+^ uptake capacity is at least, in part, the driver of the observed metabolic, transcriptomic and proteomic alterations. Indeed, we identified a number of signalling pathways that were affected by MCUa downregulation and demonstrated that MCUa_KD_ suppresses mitochondrial bioenergetics along with essential T-cell functions including cytokine secretion and migration/invasion.

Although the contribution of mitochondrial bioenergetics to the metabolic rewiring following TCR stimulation is evident, alternative non-mitochondrial signalling mechanisms that affect T-cell biology, and are affected by the changes in _m_Ca^2+^, are plausible. For example, regulation of NFAT signalling by MCU-controlled alterations in cytosolic Ca^2+^ or ROS production could also influence cellular function. In this scenario, MCU-linked Ca^2+^ uptake capacity will affect SOCE and/or cause altered mitochondrial ROS, events that have already been described to affect NFAT activity in T-cells and cancer cells (Vaeth et al, 2017; Zhang et al, 2019). Taken altogether and considering the robust functional reprogramming followed by TCR stimulation, additional unidentified mechanisms in different cellular compartments are plausible and demand further investigation.

Having in mind the contribution of MCU to T-cell biology, we tested the relevance of MCUa in EAE. In such a model, the T-cells are efficiently genetically modified ex vivo, ensuring that their encephalitogenic potential is not altered. In contrast to the conventional strategies of global or cell-specific knockout, this approach is significantly less prone to artefacts. The generated cells can be reliably tracked and functionally characterised in vivo via their fluorescence expression (Ben-Nun et al, 1981; Flügel et al, 1999; Lodygin et al, 2019). Intact global Ca^2+^ signalling has been shown to play an important role in the pathogenic potential of encephalitogenic effector T-cells (Cordiglieri et al, 2010; Lodygin et al, 2013; Ma et al, 2010). However, the contribution of _m_Ca^2+^ signalling is not yet understood. Our findings, based on two distinct EAE models, demonstrate a decreased autoimmune potential in effector T-cells lacking MCUa. Moreover, they demonstrate that T-cell migration and invasion are highly dependent on _m_Ca^2+^ dynamics. The identification of several TFs that are regulated following MCUa silencing revealed additional mechanistic cues about the functional role of MCU in T-cells. Nevertheless, future studies focusing on understanding the molecular mechanisms that link _m_Ca^2+^ uptake to effector function of CD4^+^ T-cells as well as mechanisms responsible for the differences between the two experimental EAE models (grey versus white matter), might provide additional, clinically relevant insights into the role of MCU as a therapeutic target for treating autoimmunity.

Altogether, this study demonstrates a key role of MCU in T-cell biology. Our findings suggest that T-cell-specific inhibition of MCU could be harnessed as an efficient strategy to treat T-cell-mediated autoimmunity. Combining the current knowledge regarding the design of efficient MCU inhibitors (Woods and Wilson, 2020) with additional preclinical disease models might be beneficial in understanding the therapeutic potential of mitochondrial Ca^2+^ homeostasis in T-cells as well as in other immune cells.

## Methods

**Table:** Reagents and tools table

Antibodies	Source	Identifier/Catalogue Number
CD3	Miltenyi Biotec	Cat# 130-114-710
CD4	Miltenyi Biotec	Cat# 5190304074
CD8	Miltenyi Biotec	Cat# 130-104-168
CD134-AF647	BD Biosciences	Cat #565531
CD25-BV750	Biolegend	Cat #102077
Histone H3 (1:1000)	Cell Signaling Technology	Cat# 4499S
Calnexin (1:1000)	Enzo	Cat# ADI-SPA-860-F
β-actin (1:2500)	Sigma	Cat# A5441
TBP (1:1000)	Cell Signaling Technology	Cat# 14997S
MICU1 (1:500)	Sigma	Cat# HPA037479
SDHA (1:2000)	Invitrogen	Cat# 459200
uS14m (1:500)	Proteintech	Cat# 16301-1-AP
TMBIM5/GHITM (1:1000)	Proteintech	Cat# 16296-1-AP
NDUFA9 (1:1000)	Rehling Laboratory	Mick et al, 2012
COX1 (1:1000)	Rehling Laboratory	Mick et al, 2012
COX6A (1:2000)	Rehling Laboratory	Mick et al, 2012
TACO1 (1:1000)	Rehling Laboratory	Mick et al, 2012
MITRAC12 (1:500)	Rehling Laboratory	Mick et al, 2012
ATP5B (1:10000)	Rehling Laboratory	Dennerlein et al, 2021
AFG3L2 (1:1000)	Rehling Laboratory	Poerschke et al, 2024
uL1m (1:1000)	Rehling Laboratory	Richter-Dennerlein et al, 2016
uL10m (1:1000)	Proteintech	Cat# 16652-1-AP
IRDye 800CW donkey anti-rabbit	Li-Cor	Cat# 926-32213
IRDye 680LT donkey anti-mouse	Li-Cor	Cat# 926-68022
Goat anti-rabbit IgG (H + L) HRPO	Dianova	Cat# 111-035-144
NFAT1 (1:100)	Cell Signaling Technology	Cat# D43B1
AF488-goat anti-rabbit	Thermo Fisher	Cat# A-11008

Oligonucleotides		
**RT-qPCR and T7E1 assay primers**	**Forward sequence**	**Reverse sequence**
*TBP*	CGGAGAGTTCTGGGATTGT	GGTTCGTGGCTCTCTTATC
*Orai1*	ATGAGCCTCAACGAGCACT	GTGGGTAGTCGTGGTCAG
*STIM1*	CAGAGTCTGCATGACCTTCA	GCTTCCTGCTTAGCAAGGTT
*STIM2*	GTCTCCATTCCACCCTATCC	GGCTAATGATCCAGGAGGTT
*NFAT1*	AAACTCGGCTCCAGAATCCA	TGGACTCTGGGATGTGAACT
*NFAT4*	ACCCTTTACCTGGAGCAAAC	CTTGCAGTAGCGACTGTCTT
*MCUa*	CACACAGTTTGGCATTTTGG	TGTCTGTCTCTGGCTTCTGG
*MCUb*	TTTTGCGTGTGAAGCTGTGT	TACCAAGGGAAGGCCATGT
*EMRE*	CTTGAGGAAAGATGGCGATG	CGACATAGAGAAAGGGGATCA
*MICU1*	GTGTTCAGCCCTCACAACCT	CCACCAAACTGCCTCTCAGT
*MICU2*	AGCGCTTCATGCAGTTTTCT	CAGCTGTTTGGATCCCTGAC
*MICU3*	CCAGTTTGGAAAGGCTCATC	ATTCTGAACCCTGCATGTGG
*MCUR1*	GCCCTTCCCCAGTACCAC	AGAGTTTCCTGCTCCCAGAA
*NCLX*	ATGGTGGCTGTGTTCCTGACCT	GGTGCAGAGAATCACAGTGACC
*IL-2*	TGTCACAAACAGTGCACCTA	TCAGTTCTGTGGCCTTCTTG
*IFNγ*	GCTGTTACTGCCAGGACCC	TTTTCTGTCACTCTCCTCTTTCC
*FoxP3*	CAGCACATTCCCAGAGTTCCT	AGGCAAACATGCGTGTGAAC
*GATA3*	CCCTCATTAAGCCCAAGCGA	AGTCAGGGGTCTGTTAATATTGTG
*Tbet*	TGATCATCACCAAGCAGGGAC	GTACAGGCGGTTTCCTGGCA
*RORγ-t*	GCAGCGCTCCAACATCTTCT	ACGTACTGAATGGCCTCGGT
*CD25*	AATGCACAAGCTCTGCCACT	TGCCCCACCACGAAATGATA
*CXCR3*	AGCTTTGACCGCTACCTGAA	CGGAACTTGACCCCTACAAA
*AFG3L2*	ACGAGTGATTGGTGGCTTAGAG	GCCACGTGGGATGATGGAT
*IL-6*	ACCCCCAGGAGAAGATTCCA	CACCAGGCAAGTCTCCTCATT
*IL-4*	CTTTGCTGCCTCCAAGAACAC	CCAACGTACTCTGGTTGGCT
*IL-17*	AGACCTCATTGGTGTCACTGC	CAGTCCGGGGGAAGTTCTTG
*IL-22*	GCCCTATATCACCAACCGCA	AGCGCTCACTCATACTGACTC
*IL-9*	TTGGGCATTCCCTCTGACAA	GTGGTTTGGTTGCATGGCTG
*IL-10*	CCAGACATCAAGGCGCATGT	TAGATGCCTTTCTCTTGGAGCTTA
rat-*TBP*	TAAGGCTGGAAGGCCTTGTG	TCTGCTCTAACTTTAGCACCTGT
rat-*MCUa*	TGTAATGACGCGCCAGGAAT	TGTAGCGGGTCTCTCAGTCT
MCUa sg1 T7assay	CTTTTCCATGAAGAGTCCATCATTC	TACACCCCTCCTCTAATCGCCCCTTC
MCUa sg2 ﻿T7assay	CAAATCGAACGTGCCATCCCTTACT	GTCCGGTGCTGCTGGTGCGGCCGCT
MCUa sg3 ﻿T7assay	GTACGGATTGAAATTAGCAGGAAAGC	GAGCCTCAGAACTTTAAGAATAATA

Short guide RNAs	Sequence (PAM underlined)	
Rn *MCUa* sgRNA 1	CCGGCGTCCTGGCAGAGCGTGGG	
Rn *MCUa* sgRNA 2	CCGCAGGTAGATCGCTCCTGCTG	
Rn *MCUa* sgRNA 3	CCTGGGACATCATGGAACCGGTC	

siRNAs	Sequence (5’–3’)	Overhang
siMS_Control	AGGUAGUGUAAUCGCCUUG	dTdT
siMCU_1	CAGGUGCCUUGCAAAGGUUGA	dTdT
siAFG3L2_1	CCACUGCCAAGGUCUUAAA	dTdT

Overexpression oligonucleotides	Sequence	
MCU-IFap1.f MS#1771	gtcgaggagaatcctgGGCCaATGGCGGCCGCCGCAGGTAGATCGC	
MCU IFap1.r MS#1772	CGCCCTTGCTCACCATgggcccactgcccccTTCCTTTTCTCCGATCTGTCGGAG	

Plasmids	Source/Reference	Identifier/Catalogue Number
Ratiometric MT3.1 Pericam	Nagai et al, 2001	n/a
GCaMP6s	Addgene	#40753
HyPer3	Addgene	#42131
HyPer-dMito	Evrogen	#FP942
4mt-D3cpV	Addgene	#36324
4mt-TNXL	Addgene	#51994
pcDNA3.1_MCU_flag	Rizzuto Laboratory	n/a
pcDNA3.1	Thermo Fisher	#V79020

Chemicals and other relevant reagents	Source	Identifier/Catalogue Number
Lymphocyte Separation Medium	PromoCell	Cat# C-44010
AIMV medium	Life Technologies	Cat# 12055-091
RPMI 1640 medium	Thermo Fisher	Cat# 72400021
Fetal calf serum (FCS)	Sigma-Aldrich	Cat# A9418
OptiMEM	Thermo Fisher	Cat# 31985070
Lipofectamine^TM^ 3000	Thermo Fisher	Cat# L3000001
β-synuclein peptide	Life Tein	LKPEEVAQEAAEEPLIEPL
Oligomycin	Sigma	Cat# C2759-100MG
Antimycin	Sigma	Cat# A8674
Carbonylcyanide-3-chlorophenylhydrazone (CCCP)	Sigma	Cat# C2759-100MG
Rotenone	MP Biomedicals	Cat# 215015401
Recombinant human CCL19	Peprotech	Cat# 300-29B
Recombinant human CXCL12	Peprotech	Cat# 300-28A
Recombinant rat CXCL10	BioLegend	Cat#770904
Matrigel	Corning	Cat# 354277
Poly-L-ornithine	Sigma	Cat# P3655
Thapsigargin	Sigma	Cat# T9033
Ionomycin	Sigma	Cat# I0634
Digitonin	Sigma	Cat# D141
Probenecid	Sigma	Cat# P-8761
Emetine	Merck	Cat# 324693
^35^S methionine	Hartmann Analytic	Cat# SCM-01

Dyes	Source	Identifier/Catalogue Number
BioTracker ATP-Red Live Cell Dye	Sigma	Cat# SCT045
Fura2-AM	Thermo Fisher	Cat# F1221
Rhod2-AM	Thermo Fisher	Cat# R1244
MitoTracker Green	Thermo Fisher	Cat# M7514
MitoTracker Deep Red	Thermo Fisher	Cat# M22426
Calcium-Green-5N	Thermo Fisher	Cat# C3737
TMRE	Thermo Fisher	Cat# T669
JC-1	Invitrogen	Cat# T3168
H_2_DCFDA	Thermo Fisher	Cat# D399

Enzymes	Source	Identifier/Catalogue Number
*BbsI* Fast Digest	Thermo Fisher	Cat# FD0434
*ScaI* Fast Digest	Thermo Fisher	Cat# FD0434
*ApaI* Fast Digest	Thermo Fisher	Cat# FD1414

Buffers and media	Source	
Erythrocyte lysis buffer	Custom-made (composed of 155 mM NH_4_Cl, 10 mM KHCO_3_, 0.1 mM EDTA, pH 7.3)	
Protein lysis buffer-DDM-based	Custom-made (composed of 20 mM Tris/HCl, 150 mM NaCl, 1 mM EDTA, 10% Glycerol, 0.5% DDM, 1x Protease Inhibitor)	
Protein lysis buffer-NP40-based	Custom-made (composed of 50 mM Tris/HCl, 140 mM NaCl, 2 mM PMSF, 1% NP40, 1x Protease Inhibitor, 10 mM Mg_2_Cl)	
1 mM [Ca^2+^] Ringer buffer	Custom-made (composed of 145 mM NaCl, 4 mM KCl, 10 mM Hepes (4-(2-hydroxyethyl)-1-piperazineethanesulfonic acid; pH 7.4), 10 mM Glucose, 2 mM MgCl_2_, and 1 mM CaCl_2_)	
Rat T-cell re-stimulation medium	Custom-made (DMEM-based medium containing 1% nonessential amino acids, 1% sodium pyruvate, 1% penicillin-streptomycin, glutamine, asparagine, 4 µL/L β-mercaptoethanol, 5% IL-2-containing supernatant, and 1% rat serum)	

Kits	Source	Identifier/Catalogue Number
CD4^+^ T-cell isolation kit	Miltenyi Biotec	Cat# 130-096-533
Dynabeads^TM^ Human T-cell Activator	Gibco	Cat# 11132D
ImmunoCult^TM^ Human CD3/CD28 T-cell Activator	StemCell Technologies	Cat# 10991
P3 Primary Cell 4D Nucleofector™ X Kit	Lonza	Cat# V4XP-3024
CellTiter Blue^®^ Cell Viability assay kit	Promega	Cat# G8080
NucleoSpin^TM^ RNA Plus kit	Macherey-Nagel	Cat# 740984.50
RNAEasy isolation kit	Qiagen	Cat# 74004
SuperScript IV Reverse Transcriptase kit	Invitrogen	Cat# 18090200
IL-2 Lumit^TM^ human Immunoassay kit	Promega	Cat# W6020
IFN gamma Human Uncoated ELISA Kit	Thermo Fisher	Cat# 88-7316-86
Cell Titer Glo^®^ Luminescent Cell Viability assay kit	Promega	Cat# G9241
Complex I Human Enzyme Activity Assay Kit	Abcam	Cat# ab109721
Complex IV Human Enzyme Activity Assay Kit	Abcam	Cat# ab109909

Relevant instrumentation	Source	
Amaxa 4D Nucleofector^®^	Lonza	
Berthold, Mithras LB 940 microplate reader	Berthold Tech	
Stratagene Mx3000P	Agilent	
HiSeq 4000 sequencing system	Illumina	
Odyssey^®^ CLx system	Li-Cor	
Amersham ImageQuant^TM^ 800 system	Cytiva Life Sciences	
Q Exactive^TM^ Plus Orbitrap mass spectrometer	Thermo Fisher	
Fusion Lumos mass spectrometer	Thermo Fisher	
CLARIOstar microplate reader	BMG LabTech	
Seahorse XFe Analyser	Agilent	
Zeiss D1 Cell Observer fluorescent microscope	Zeiss	
Axiovert S100TV fluorescence microscope	Zeiss	
Zeiss Laser Scanning Confocal Microscope 710	Zeiss	
Nikon Ti inverted fluorescence microscope	Nikon	
Countess^TM^ 3 Automated Cell Counter	Thermo Fisher	
Synergy H1 microplate reader	BioTek	
CytoFLEX flow cytometer	Beckmann Coulter	
FACS Canto II	BD Biosciences	

Relevant software	Source	
FlowJo v10	FlowJo	
MxPro v4.10	Agilent	
Python programming language frameworks		
KNIME v5.1.2	Knime Analytics	
Image Studio^TM^ Lite	Li-Cor	
Maxquant v2.0.3.0		
Seahorse Wave Desktop Software	Agilent	
VisiView v4.2.0.0	Visitron Systems GMbH	
Zen v2.6	Zeiss	
Huygens Professional v23.04		
CytExpert	Beckmann Coulter	
GraphPad Prism v9, v10		

Other	Reference	
MitoCarta3.0	Rath et al, 2021	
Bionic Visualizations	Liebermeister et al, 2014	
PANTHER	Mi et al, 2019	
DAVID Tools	Sherman et al, 2022	
Perseus v1.6.6.0		
HOMER workflow *findMotifs.pl*		

### Cell culture and genetic manipulation

#### Primary human CD4^+^ T-cells

Human peripheral blood mononuclear cells (PBMCs) were isolated from leukoreduction system chambers obtained from healthy anonymous donors at a local department of Transfusion Medicine (Ethical approval 2/3/18) by density gradient centrifugation in Lymphocyte Separation Medium 1077 (PromoCell, Cat# C-44010), followed by erythrocyte lysis using a custom-made lysis buffer (Reagents and Tools Table). Primary human naive CD4^+^ T-cells were isolated from the obtained PBMCs by negative microbeads isolation (Miltenyi Biotec, Cat# 130-096-533), following manufacturer’s instructions. Isolated naive CD4^+^ T-cells were maintained in AIMV medium (Life Technologies, Cat# 12055-091) supplemented with 10% fetal calf serum (FCS) (Sigma, Cat# A9418). Naive CD4^+^ T-cell purity after isolation was confirmed by flow cytometry analysis, by staining the cells with anti-CD3 (Miltenyi Biotech, Cat# 130-114-710), anti-CD4 (Miltenyi Biotech, Cat# 5190304074) and anti-CD8 (Miltenyi Biotech, Cat# 130-104-168) markers (FACS Canto II, BD Biosciences). Effector CD4^+^ T-cells were generated by activation for 12, 24, 48, or 72 hrs with 50 µL of Dynabeads^TM^ Human T-cell Activator CD3/CD28 (Gibco, Cat# 11132D) (1:1 bead-to-cell ratio) or ImmunoCult^TM^ Human CD3/CD28 T-cell Activator (StemCell Tech, Cat# 10991). Effector CD4^+^ T-cell re-stimulation was performed by addition of 30 µL of Dynabeads^TM^ Human T-cell Activator CD3/CD28 or ImmunoCult^TM^ Human CD3/CD28 T-cell Activator for 8 hrs prior to cell harvest. ImmunoCult^TM^ Activator antibody concentration was titrated according to the type of experiment performed (0.5–25 µL/mL).

#### Cloning of expression constructs for downregulation of rat MCUa gene and generation of retroviral packaging cell lines

With the aim to generate stable rat effector CD4^+^ T-cell lines with MCUa downregulation, three different short guide (sg) RNAs were designed for targeting *MCUa* (each one targeted to a different site of the MCUa gene (refer to Reagents and Tools Table for sequence information). One sgRNA was designed as a control, targeting the ubiquitously-expressed non-coding locus *Rosa26*. Each sgRNA construct was cloned into the *Bbs*I site (Fast Digest, Thermo Fisher, Cat# FD0434) downstream of the U6 RNA polymerase III promoter. To enable mitochondrial Ca^2+^ detection, the retroviral expression vector was modified by replacing the Turquoise fluorescent protein with a fusion Scarlet-GCaMP6s protein targeted to the mitochondrial matrix (2mt) (refer to Fig. EV4A).

After establishing the vectors, verifying them by sequencing (SeqLab, Microsynth AG, Germany), and confirming their targeting efficiency in HEK293 cells transiently co-transfected with rat MCU-mCherry and Cas9 expression constructs, four stable packaging cell lines (MCUa sg1, -2, -3 and sgRosa26) were established using GP + E86 cells (refer to (Markowitz et al, 1988) for details). Briefly, 1–2 µg of Puro2A-2mt-Scarlet-GCaMP6s-U6sgRNA plasmid was linearized by digestion with *Sca*I restriction enzyme (Fast Digest, Thermo Fisher, Cat# FD0434) and precipitated by 1:1 addition of isopropanol followed by centrifugation (maximal speed, 10 min, RT). The DNA pellet was dried and resuspended in 50 µL of OptiMEM (Thermo Fisher, Cat# 31985070). Next, 2.5 µL of Lipofectamine^TM^ 3000 reagent (Thermo Fisher, Cat# L3000001) were mixed with 47.5 µL of OptiMEM and the resuspended DNA solution. After incubation at RT for 10 min, the transfection mixture was added dropwise to the GP + E86 cells, previously seeded in 12-well plates (40–60% confluency). Two days after transfection, the cell lines were seeded onto T75 cell culture flasks and selection using 1 µg/mL puromycin started. After 7 days of selection, the cells with the brightest Scarlet-GCaMP6s signal were enriched from the pool of puromycin-resistant GP + E86 by FACS sorting (FACS Aria II cytometer, Beckton Dickinson).

#### Cloning of overexpression constructs for rat MCUa and generation of retroviral packaging cell lines

The sequence of rat *MCUa* open reading frame without stop codon was PCR-amplified from Lewis rat cDNA obtained from effector T-cells using the following oligonucleotides:

gtcgaggagaatcctgGGCCaATGGCGGCCGCCGCAGGTAGATCGC *MCU-IFap1.f MS#1771* and CGCCCTTGCTCACCATgggcccactgcccccTTCCTTTTCTCCGATCTGTCGGAG *MCU-IFap1.r MS#1772*, combined with CloneAmp polymerase mix (Takara). The resulting PCR product was isolated from an agarose gel and cloned into *Apa*I-digested (Fast Digest, Thermo Fisher, Cat# FD1414) MSCV-PuroP2A-tdTomato plasmid upstream of and in-frame with the Tomato-coding sequence using the InFusion assembly mix (Takara) (refer to Appendix Fig. S4G).

After verification of construct integrity by sequencing, GP + E86 packaging cell lines were generated: a control Tom70-Scarlet cell line (mitochondrial localisation signal fused to a red fluorescent protein) and an MCUa-mTomato overexpression cell line, as described in the previous section.

#### Primary rat CD4^+^ effector T-cell generation and culture

Rat CD4^+^ T-cell lines were established as described (Lodygin et al, 2019). Draining lymph nodes were aseptically dissected and placed in ice-cold Re-stimulation medium (DMEM-based medium containing 1% nonessential amino acids, 1% sodium pyruvate, 1% penicillin-streptomycin, glutamine, asparagine, 4 µL/L β-mercaptoethanol, 5% IL-2-containing supernatant, and 1% horse serum). The tissue was homogenised by pressing it through a metal strainer. Cell suspension was collected and 20 × 10^6^ lymph node cells were seeded in U-bottom 96-well plates on top of previously-seeded (1.5 × 10^4^) GP + E86 cell lines, in Re-stimulation medium containing 20 µg/mL myelin-basic protein (MBP, isolated from guinea pig brain) or β-synuclein. On day 2 after the start of primary cell culture, 50 µL of T-cell growth factor (TCGF - T-cell medium supplemented with 10% horse serum and 1:50 diluted supernatant from IL-2 producing cell lines) were added per well in all of the prepared U-plates. On day 4, the generated T-cells were transferred to flat-bottom 96-well plates and supplemented with 50 µL of TCGF and 1 µg/mL puromycin for selection. The first T-cell re-stimulation was performed on day 7 after start of culture, by removing 100 µL of supernatant and adding 1.4 × 10^6^ irradiated (30 Gy) Lewis thymocytes per well. Experiments with the MCU knockdown cell lines were performed 4–5 days after second and third re-stimulation cycles, whereas with the MCU overexpression cell lines they were performed after the first re-stimulation cycle to minimise possible secondary adaptation effects caused by the overexpression.

#### Transient cell transfection

Introduction of plasmid DNA (Reagents and tools table) in primary human CD4^+^ T-cells was achieved by nucleofection (Amaxa 4D Nucleofector^®^, Lonza GmbH, Cologne, Germany). Briefly, 2–3 × 10^6^ naive or effector T-cells were transfected using the P3 Primary Cell 4D Nucleofector™ X Kit (Lonza, Cat# V4XP-3024) and 1 µg/mL plasmid DNA, according to manufacturer’s instructions (electroporation pulse programmes: naive T-cells—EH-100; effector T-cells—E0-115). In the event of T-cell activation with magnetic beads, the beads were removed by magnetic separation using a DynaMag-15^TM^ magnet (Invitrogen, Cat# 12301D) prior to transfection. After transfection, T-cells were maintained in RPMI 1640 medium (Thermo Fisher Cat# 72400021) supplemented with 10% FCS.

Introduction of small interfering (si) RNA (Reagents and tools table) was achieved by transfecting 5–6 × 10^6^ naive or effector CD4^+^ T-cells using the same P3 Primary Cell Nucleofector™ kit and 2 µM of siRNA working solution. Experiments were performed 48–72 hrs after siRNA delivery. Knockdown efficiency was confirmed via RT-qPCR and immunoblotting.

### Assessment of cell proliferation

For estimation of the number of viable proliferating and metabolically active cells, the CellTiter Blue^®^ Cell Viability assay kit (Promega, Cat# G8080) was used per manufacturer’s instructions. Proliferation was assessed by measuring resorufin fluorescence intensity at 2, 24, 48, and 72 hrs after cell seeding by using a Berthold, Mithras LB 940 microplate reader (Berthold Tech, Cat# 4300). Absolute fluorescence signal was plotted against the time of measurement and different cell lines or conditions were compared.

### Determination of gene expression

Total RNA from human CD4^+^ T-cells was isolated using the NucleoSpin^TM^ RNA Plus kit (Macherey-Nagel, Cat# 740984.50), according to manufacturer’s instructions. RNA isolation from rat CD4^+^ T-cells was performed using the RNAEasy isolation kit (Qiagen, Cat# 74004), according to manufacturer’s instructions. Subsequent cDNA synthesis was performed using 200-800 ng of isolated RNA per sample, by addition of 1 µL of Oligo d(T)20 (50 μM) (Invitrogen, Cat# 18418-020) and 1 μL of dNTP (10 mM) (Invitrogen, Cat# 18427-013), followed by a denaturation step (5 min, 65 °C). Reverse transcription was performed by using the SuperScript IV Reverse Transcriptase kit (Invitrogen, Cat# 18090200) (10 min at 50 °C, followed by 10 min at 80 °C). To quantify gene expression, several validated primer pairs were used (refer to Reagents and Tools Table). Synthesized cDNA and primer pairs were added to a master mix (2x GoTaq^®^ qPCR Master Mix, Promega, Cat# A6001). Amplification and detection were performed using the Stratagene Mx3000P system. The Ct values were analysed in the MxPro 4.10 software and normalised to the housekeeping (HK) gene *TBP* and to primer E-values using the 2^-ΔCt^ method.

### RNA sequencing

Samples containing 1500 ng RNA were sequenced in the Institute of Human Genetics in Göttingen. The HiSeq 4000 sequencing system (Illumina) was used to perform detailed RNA sequencing. Processed RNA-seq data, by using DEseq2, were utilised to identify differentially-expressed genes (DEGs). Candidates for the ‘*naive-effector*’ dataset were obtained using a *q*-value (Benjamini-Hochberg-corrected) cut-off of 0.05. Candidates for the ‘*siControl-siMCUa*’ dataset were selected using a *p*-value cut-off of 0.05. Both datasets were further filtered by an absolute logarithmical fold change (log2 FC) of 0.5 and sorted as up- or down-regulated depending on the fold change (up—FC > 0; down—FC < 0). Significant DEGs were illustrated using volcano plots. DEGs of both datasets were filtered based on the MitoCarta3.0 dataset (Rath et al, 2021). MitoCarta and non-MitoCarta-related DEGs were further investigated using pathway analysis. Proteomaps were generated by using Bionic Visualizations (Liebermeister et al, 2014) based on either up- or downregulated non-MitoCarta-related DEGs. Functional annotation of gene ontology of all non-MitoCarta-related DEGs was done using PANTHER (Mi et al, 2019). KEGG Pathways were obtained using DAVID Tools (Sherman et al, 2022). Annotated terms and pathways were filtered by a *q*-value cut-off of 0.05. MitoCarta DEGs were analysed using annotated pathways from MitoCarta3.0 by performing Fisher’s Exact test. Pathway analysis results were filtered by a *q*-value (Benjamini-Hochberg-corrected) cut-off of 0.05.

### Immunoblotting

At least 30 µg of extracted protein (using either a DDM-based or an NP-40-based lysis buffer; refer to Reagents and tools table) were separated using 10–12% sodium dodecyl sulfate polyacrylamide gel electrophoresis (SDS-PAGE) and transferred onto 0.2 µm nitrocellulose membranes (Trans-Blot^®^ Turbo^TM^ Transfer System, BioRad). Primary antibody incubations were performed overnight at 4 °C, followed by three washing steps with 1x tris-buffered saline containing Tween^®^ 20 (TBS-T). Secondary antibody incubation was performed at RT for 1 h, followed by three additional washing steps with 1x TBS-T. Imaging was performed using the Odyssey^®^ CLx system or the Amersham ImageQuant^TM^ 800 system. Bands were quantified using Image Studio^TM^ Lite (normalisation with background, and loading and/or sample controls). Refer to Reagents and Tools Table for detailed antibody information.

### Proteomics

#### Human CD4^+^ T-cells

At least 1 × 10^7^ naive and 0.5 × 10^7^ activated CD4^+^ T-cells at different time points (12, 24, 48, and 72 h) were collected and washed twice with ice-cold 1xDPBS. Cell pellets were snap-frozen in liquid nitrogen and stored at −80 °C until further use. The proteomics core facility from CECAD Cologne performed the mass spectrometry. After thawing naive and activated CD4^+^ T-cell pellets, 20 µL lysis buffer (4% SDS in PBS containing protease inhibitor) were added and the samples were sonified. Afterwards, the samples were boiled for 5 min at 96 °C to precipitate the proteins. To remove detergents, a fourfold volume of ice-cold acetone was added and samples were frozen at −80 °C overnight. The next day, samples were thawed and centrifuged for 15 min at 16,000 × *g* at 4 °C. Supernatant was removed, pellets were washed twice with 500 µL of ice-cold acetone and then air-dried. Cell pellets were lysed in 50 µL urea buffer (8 M urea and 50 mM TEAB containing protease inhibitor) and sonified. The samples were centrifuged for 15 min at 20,000 × *g* and the supernatants were transferred to a clean tube. Protein concentration was determined using the Pierce Protein Assay Reagent (Thermo Fisher Scientific, #22660), according to the manufacturer’s protocol. From each sample, 50 µg of protein were reduced with DTT, to a final concentration of 5 mM, for 1 hrs at 37 °C. Afterwards, chloroacetamide (CAA), with a final concentration of 40 mM, was added for 30 min in the dark at RT. Lys-C was used to digest the peptides, with an enzyme:substrate ratio of 1:75 and incubation of 4 hrs at 25 °C. Next, samples were diluted with TEAB buffer (50 mM) to reduce the urea concentration to <2 M before adding trypsin. Trypsin was added with an enzyme:substrate ratio of 1:75 and incubated at 25 °C overnight. To terminate the digestion, 1% formic acid was added to the samples. Two-layer SDB-RPS stage tips were used for desalting/mixed-phase clean-up. Mass spectrometry was performed by the proteomics core facility from CECAD Cologne, followed by data processing and analyses.

Prepared samples were loaded on a Q Exactive^TM^ Plus Orbitrap mass spectrometer coupled to an EASY nLC (both Thermo Fisher Scientific). Raw data analysis was performed using Maxquant (version 2.0.3.0). Missing values were imputed using normal distribution. Differentially expressed proteins (DEPs) were identified using a *q*-value cut-off of 0.05 and sorted as up- or down-regulated depending on the fold change (up—FC > 0; down—FC < 0). Significant DEPs were illustrated using a timeline of volcano plots. DEPs were filtered based on the MitoCarta3.0 dataset (Rath et al, 2021). MitoCarta and non-MitoCarta-related DEPs were further investigated using pathway analysis. Proteomaps were generated by using Bionic Visualizations based on either up- or down-regulated non-MitoCarta-based DEPs. Functional annotation of gene ontology of all non-MitoCarta-related DEPs was done using PANTHER (Mi et al, 2019). KEGG Pathways were obtained using DAVID Tools (Sherman et al, 2022). Annotated terms and pathways were filtered by a *q*-value (Benjamini-Hochberg-corrected) cut-off of 0.05. MitoCarta DEPs were analysed using annotated pathways from MitoCarta3.0 by performing Fisher’s Exact test. Pathway analysis results were filtered by a *q*-value cut-off of 0.05.

#### Rat CD4^+^ T-cells

At least 5 × 10^6^ rat CD4^+^ T-cells (control, sgMCUa_KD_ 1 and sgMCUa_KD_ 2 cell lines) were collected and washed twice with ice-cold 1xDPBS. Cell pellets were frozen and stored at −80 °C until further use. Initially, thawed T-cell pellets were lysed in an aqueous buffer containing 7 M urea, 2 M thiourea, 4% (*w*/*v*) 3-[(3-Cholamidopropyl) dimethylammonio]-1-propanesulfonate and 30 mM 2-Amino-2-(hydroxymethyl) propane-1,3-diol (pH=8), as described earlier (Brenig et al, 2020). 10 µg of protein lysates were prepared for mass spectrometric (MS) analysis by the single-pot, solid-phase-enhanced sample preparation method including reduction, alkylation and digestion with trypsin, as described (Hughes et al, 2019). 500 ng of resulting peptides were firstly separated by an U3000 rapid separation liquid chromatography system (Thermo Fisher Scientific) on 25 cm C18 column using a 2 hrs gradient, as described (Brenig et al, 2020). Peptides were injected via an electrospray nano-source interface into a Fusion Lumos mass spectrometer (Thermo Fisher Scientific) operated in data-independent positive mode. The mass spectrometer was equipped with a high field asymmetric waveform ion mobility spectrometry device operated with a compensation voltage of −50 V. First, a precursor spectrum was recorded in the Orbitrap analyser (resolution 60,000, scan range 380–985 *m*/*z*, automatic gain control target 400,000, maximum injection time 100 mseconds, profile mode). Next, precursors were selected within isolation windows of 10 *m*/*z* (with an overlap of 1 *m*/*z*) within a precursor mass range of 380–980 *m*/*z*. After fragmentation by higher energy collisional dissociation (30% collision energy, 5% stepped collision energy), fragment spectra (scan range 145–1450 *m*/*z*) were recorded in the Orbitrap analyser at a resolution of 15,000 (automatic gain control target 100,000, maximum injection time 40 mseconds, centroid mode). Cycle time was set to 3 s. Mass spectrometry data was analysed with DiaNN (version 1.8.1) using standard settings (except enabled methionine oxidation as a variable modification). Within DiaNN, a spectral library was predicted from contaminant sequences from MaxQuant 2.1.0. 0 and 47945 *Rattus Norvegicus* protein entries downloaded from UniProt knowledge base on 12.01.2023 (UP000002494).

Quantitative and identification data was processed with custom R (version 1.0.1) scripts and within the Perseus framework (version 1.6.6.0). Proteins were filtered for containing at least two peptides and no missing quantitative values, and potential contaminants were removed. Annotations were added from Perseus and UniProt (06.10.2023). To detect differences between the groups, an ANOVA was performed including permutation-based false discovery rate (FDR) truncation (5% FDR, 250 randomizations, S0 = 0.1) and Tukey’s honestly significant difference (THSD) post hoc tests (5% FDR). Proteins were selected as differentially expressed according to the following criteria: (1) Significant ANOVA *p*-value (5% FDR); (2) Significant THSD (5% FDR) for comparison of sgMCUa_KD_ 1 vs Control; (3) Significant THSD (5% FDR) for comparison of sgMCUa_KD_ 2 vs Control; and (4) Knockdown-induced abundance change in the same direction for both mentioned comparisons. Selected proteins were used for enrichment and network analysis using String version 12 and Cytoscape (version 3.10.1; ClueGo v 2.5.10) with all identified proteins as a background set. Proteomaps were generated by using Bionic Visualizations based on either up- or down-regulated DEPs. Functional annotation of gene ontology of all non-MitoCarta-related DEPs was done using PANTHER (Mi et al, 2019). KEGG Pathways were obtained using DAVID Tools (Sherman et al, 2022). Annotated terms and pathways were filtered by a *q*-value (Benjamini-Hochberg-corrected) cut-off of 0.05.

#### HOMER analyses

For each dataset, differentially expressed proteins were identified and merged, yielding a total of 556 regulated protein names. To uncover potential regulatory motifs, the HOMER workflow *findMotifs.pl* was employed using the identified proteins, conducting motif analysis against a background of GC-balanced sequences (16,111 background sequences). The top five most enriched motifs were illustrated along with their *q*-values (Benjamini-Hochberg-corrected).

### Quantification of IL-2 secretion

IL-2 secretion was assessed by using the Lumit^TM^ human Immunoassay kit (Promega, Cat# W6020) based on the NanoLuc^®^ luciferase. Supernatants collected from 2 × 10^6^ re-stimulated human T-cells were diluted 1:100 and used for IL-2 measurements according to manufacturer’s instructions. Average luminescence readings (RLU) for replicate wells were calculated using the CLARIOstar microplate reader and the average zero-background control was subtracted from each sample and standard. A standard curve was generated from the known standard concentrations and was used to calculate the cytokine concentration of unknown samples.

### IFNγ enzyme-linked immunosorbent assay (ELISA)

Secretion of IFNγ was assessed using the IFN gamma Human Uncoated ELISA Kit (Thermo Fisher, Cat# 88-7316-86). Supernatants collected from 2 × 10^6^ re-stimulated T-cells were diluted 1:50 and utilised according to manufacturer’s instructions. 3,3’,5,5-tetramethylbenzidine (TMB) absorbance was measured after addition of 2N H_2_SO_4_ stop solution, using the CLARIOstar microplate reader. Values at 570 nm were subtracted from values at 450 nm for each well. Average values for replicate wells were calculated and the average zero-background control was subtracted from each standard value. A standard curve was generated from the known standard concentrations and was used to calculate IFNγ concentration of unknown samples.

### Determination of mitochondrial respiration

Oxygen consumption rates (OCR) and extracellular acidification rates (ECAR) were measured as previously described (van der Windt et al, 2016), with minor modifications, with the aid of the Seahorse XFe Analyser (Agilent). Briefly, 2 × 10^5^ T-cells were seeded per well in a 96-well Seahorse plate (Agilent) (coated with 50 µg/mL poly-D-lysine overnight) in Seahorse XF DMEM (Agilent, #103575-100) supplemented with 4.5 g/L glucose, 1 mM Na-pyruvate and 2 mM L-glutamine. A sensor cartridge was hydrated with Seahorse XF calibrant solution (Agilent, Cat# 100840-000), incubated in a non-CO_2_ incubator (37 °C) (Agilent) overnight and loaded with the respective drugs on the day of measurement: Oligomycin (Sigma, Cat# O4876-5MG), carbonylcyanide-3-chlorophenylhydrazone; CCCP (Sigma, Cat# C2759-100MG), and Antimycin A (Sigma, Cat# A8674) plus Rotenone (MP Biomedicals, Cat# 215015401). Periodic measurements were performed at basal state and after injection of the respective drugs. Normalisation according to viable cell number was performed by using the CyQUANT™ Cell Proliferation Assay kit (Thermo Fisher, Cat# C7026), following manufacturer’s instructions. Quantification was performed using the Seahorse Wave Desktop Software.

### Assessment of cellular ATP levels

Estimation of global levels of ATP in cells was performed using the Cell Titer Glo^®^ Luminescent Cell Viability assay kit (Promega, Cat# G9241) according to manufacturer’s instructions. Briefly, 2 × 10^5^ T-cells were seeded per well in an opaque 96-well plate in their respective culture medium. ATP standards were prepared at concentrations ranging from 10 nM to 500 µM. The Cell Titer Glo^®^ reagent was added onto the cells and the plate was incubated for 15 min under low-light conditions at RT. Luminescence levels (RLU) were measured by using the CLARIOstar (BMG LabTech) microplate reader. ATP levels were calculated by using the generated standard curve average values.

### Assessment of mitochondrial ATP levels using live-cell imaging

Mitochondrial ATP levels were measured by staining 2 × 10^5^ T-cells with 5 µM of BioTracker ATP-Red Live Cell Dye (Sigma, Cat# SCT045) for 15 min at 37 °C. Measurements were performed at 37 °C in 1 mM [Ca^2+^] Ringer’s buffer solution (containing 145 mM NaCl, 4 mM KCl, 10 mM Hepes (4-(2-hydroxyethyl)-1-piperazineethanesulfonic acid; pH 7.4), 10 mM Glucose, and 2 mM MgCl_2_) by live single-cell fluorescence imaging on a fluorescence Zeiss D1 Cell Observer microscope equipped with a 40x/1.3 oil Neofluar objective, Axiocam 702 mono and LED system (Colibri, Zeiss). Images were acquired upon excitation at 555 nm (excitation filter: 550/32 nm) together with a 573 nm dichroic mirror and 630/92 nm emission filter. 5 mM external Mg^2+^-ATP (Sigma, Cat# A-9062) and 2 µM external CCCP (Sigma, Cat# C2759-100MG) were added as a control. Acquisition was performed with the Zen 2.6 software, data processing and statistics with Microsoft Excel 2019, and plotting with GraphPad Prism 9. Single cell measurement points were analysed by marking only the mitochondrial area, and plotted as the mean ATP-Red fluorescence intensity (normalised to background fluorescence) over time (minutes).

### Trans-well CD4^+^ T-cell migration/invasion

CD4^+^ T-cells were kept in starvation medium (RPMI or DMEM culture medium w/o FCS) for 3 hrs in standard culture conditions. Subsequently, 750 µL of conditioned medium (CD4^+^ T-cell supernatant collected after 48 hrs of culture, supplemented with 20% FCS and 50 ng/mL recombinant human CCL19, Peprotech Cat# 300-29B, and CXCL12, Peprotech Cat# 300-28A), or recombinant rat CXCL10 (BioLegend, Cat#770904) were added per well (in triplicate) in a 24-well plate. Following, 1 × 10^6^ starved T-cells were subjected to a trans-well assay for 4 hrs by seeding them in 5 µm pore inserts previously coated with 40 µL of Matrigel (1:1 dilution in RPMI or DMEM w/o FCS, Corning, Cat# 354277). Quantification of total migrated cells in T-cell conditioned medium was achieved by cell counting using the Countess^TM^ 3 Automated Cell Counter (Thermo Fisher).

### Cytosolic Ca^2+^ measurements

Fura2-AM-based cytosolic [Ca^2+^] measurements in T-cells were performed as outlined in (Gibhardt et al, 2020) and (Bogeski et al, 2010). Briefly, 2 × 10^5^ cells were loaded with 1 µM Fura2-AM (Thermo Fisher, Cat# F1221) in culture medium for 30 min at RT and allowed to attach for 20 min at RT onto a 25 mm coverslip (VWR^®^ Cat# 631-0172) previously coated with 100 µg/mL of poly-L-ornithine (diluted in ddH_2_O) (Sigma, Cat# P3655). Cells were washed once with Ringer’s buffer (containing 145 mM NaCl, 4 mM KCl, 10 mM Hepes (4-(2-hydroxyethyl)-1-piperazineethanesulfonic acid; pH 7.4), 10 mM Glucose, 2 mM MgCl_2_, and 0.5 mM CaCl_2_, 1 mM CaCl_2_ or 0 mM CaCl_2_ with 1 mM EGTA and measurements were performed at RT and ER Ca^2+^ store depletion was achieved using 1 µM of the SERCA inhibitor thapsigargin (Tg; Sigma, Cat# T9033) in 0 mM CaCl_2_ with 1 mM EGTA, while 1 mM CaCl_2_ was used for re-addition of external Ca^2+^, as indicated in the figures. Physiological Ca^2+^ uptake in naive CD4^+^ T-cells was stimulated using anti-CD3/CD28 Dynabeads^TM^ or antibody solution. Ratiometric time-lapse imaging was performed on a fluorescence microscope equipped with an Axiovert S100TV (Zeiss), pE340Fura (CoolLED) light source with LED 340 nm (excitation at 340/20 nm) and 380 nm (excitation at 380/20 nm), a T400 LP dichroic mirror, 515/80 nm emission filter, a Fluar 40x/1.3 oil objective, and a sCMOS camera pco.edge. Bead-activated T-cells were chosen for analysis by selecting only the cells that came into contact with a bead throughout the measurement. When antibody solution was used, all cells were selected. Background-corrected data were exported with the VisiView 4.2.0.0 software (Visitron Systems GmbH, Germany), analysed using Microsoft Excel 2019, and plotted as average ratio of F340/380 nm (and when indicated normalised to T-cell volume (in µm^3^)) over time (minutes), using GraphPad Prism 9.

### Mitochondrial Ca^2+^ measurements

#### Human CD4^+^ T-cells

Mitochondrial [Ca^2+^] (_m_Ca^2+^) in human T-cells was measured using the ratiometric protein sensor MT3.1 Pericam (Nagai et al, 2001) or the FRET-based _m_Ca^2+^ protein sensors 4mtD3cpV (Addgene #36324) and 4mtTNXL (Addgene #51994) in Ringer’s buffer containing 1 mM Ca^2+^ at 37 °C. Measurements were performed by live single-cell fluorescence imaging on a fluorescence Zeiss D1 Cell Observer microscope equipped with a 40x/1.3 oil Neofluar objective, Axiocam 702 mono and LED system (Colibri, Zeiss). Images were acquired upon excitation at 420 nm (excitation filter: 420/40 nm) and 505 nm (excitation filter: 500/15 nm) together with a 515 nm dichroic mirror and 539/25 nm emission filter for MT3.1 Pericam. FRET-based mitochondrial Ca^2+^ sensors (4mt‐D3cpV and 4mt-TNXL) were measured by using CFP (excitation: 420/40 nm, emission: 483/32 nm, beam-splitter 458 nm) and YFP (excitation: 505/15 nm, emission: 542/27 nm, beam-splitter 530 nm). Acquisition was performed with the Zen 2.6 software, data processing and statistics with Microsoft Excel 2019, and plotting with GraphPad Prism 9. Single cell measurement points were analysed by marking only the mitochondrial area of the cells, and plotted as a MT3.1 Pericam 420/505 nm ratio (normalised to background fluorescence and T-cell volume when required) over time (minutes). FRET was calculated using the background and bleed-through corrected FRET/donor ratio (Eq. 1):

1 $$	{{{\rm{FRET}}}}/{{{\rm{donor}}}}\; {{{\rm{ratio}}}}=\\ 	\quad\frac{\left({{{{\rm{FRET}}}}}-{{{{\rm{background}}}}}\right)-\left[\left({{{{\rm{donor}}}}}-{{{{\rm{background}}}}}\right)\cdot {{{{\rm{CFd}}}}}\right]-\left[\left({{{{\rm{acceptor}}}}}-{{{{\rm{background}}}}}\right) \cdot {{{{\rm{CFa}}}}}\right]}{\left({{{{\rm{donor}}}}}-{{{{\rm{background}}}}}\right)}$$

CF: correction factor for donor (d) and acceptor (a) bleed-through.

Human T-cells overexpressing MCUa were co-stained with the mitochondrial indicator Rhod2-AM (500 nM; Thermo Fisher, Cat# R1244) and MitoTracker Green (500 nM; Thermo Fisher, Cat# M7514). Images were acquired upon excitation at 505 nm (excitation filter: 500/15 nm) together with a 515 nm dichroic mirror and 539/25 nm emission filter for MitoTracker Green, and 555 nm (excitation filter: 550/32 nm) together with a 573 nm dichroic mirror and 630/92 nm emission filter for Rhod2-AM. Data acquisition was performed using the Zen 2.6 software, data processing and statistics with Microsoft Excel 2019, and plotting with GraphPad Prism 9. Data were analysed by marking only the mitochondrial area of the cells, and plotted as a Rhod2/MitoTracker Green ratio over time (minutes).

Physiological _m_Ca^2+^ uptake by the cells was achieved by addition of anti-CD3/CD28 Dynabeads^TM^ or antibody solution. Non-physiological stimulation was achieved by addition of 1 µM thapsigargin or 4 µM ionomycin (Sigma, Cat# I0634).

Mitochondrial Ca^2+^ uptake in naive and effector T-cells was additionally indirectly assessed using a Calcium Retention Capacity (CRC) assay with digitonin-permeabilized cells. Briefly, 400,000 cells were permeabilized in 100 µL 0 mM Ca^2+^ Ringer’s buffer solution (containing 1 mM EGTA) with 15 µM digitonin (Sigma, Cat# D141) for 10 min at RT. Digitonin was removed by a short centrifugation step and the cells were resuspended in 50 µL Ringer’s buffer containing 0 mM Ca^2+^, 1 mM EGTA and 1 µM Calcium-Green-5N (Thermo Fisher, Cat# C3737) and seeded in a 96-well plate (in duplicate). Calcium-Green-5N fluorescence intensity (excitation: 488/15 nm, emission: 535/20 nm) was detected every 12 s using the CLARIOstar (BMG LabTech) microplate reader and 4 µL of 2 mM Ca^2+^ containing Ringer’s buffer were subsequently were applied to induce _m_Ca^2+^ uptake, observed as a rapid decrease in total fluorescence upon Ca^2+^ addition.

#### Rat CD4^+^ T-cells

Rat CD4^+^ effector T-cells with sgMCUa_KD_ or overexpression (rat MCU o/e 2 cell line) were designed to stably express the Ca^2+^ protein biosensor GCaMP6S, containing a mitochondrial targeting sequence (2mt) and a fluorescent Scarlet-tag. The generated cell lines were used to measure basal [_m_Ca^2+^] and _m_Ca^2+^ uptake at 37 °C upon both physiological and non-physiological stimulation via live single-cell fluorescence imaging on a fluorescence Zeiss D1 Cell Observer microscope equipped with a 40x/1.3 oil Neofluar objective, Axiocam 702 mono and LED system (Colibri, Zeiss). Images were acquired upon excitation at 505 nm (excitation filter: 500/15 nm) and 555 nm (excitation filter: 550/32 nm) together with 515 and 573 nm dichroic mirrors, and 539/25 and 630/92 nm emission filters. Data acquisition was performed using the Zen 2.6 software, data processing and statistics with Microsoft Excel 2019, and plotting with GraphPad Prism 9. Data were analysed by marking only the mitochondrial area of the cells, and plotted as a 505/555 nm ratio (normalised to background fluorescence) over time (minutes).

Rat CD4^+^ effector T-cells with MCU overexpression (rat MCU o/e 1 cell line) were additionally transiently transfected using the FRET-based _m_Ca^2+^ sensor 4mt-D3cpV (Addgene #36324) as described above.

### Mitochondrial membrane potential measurements

#### TMRE measurements

Mitochondrial membrane potential (∆Ψm) in CD4^+^ T-cells was measured using the dye tetramethylrhodamine ethyl ester (TMRE) (Thermo Fisher, Cat# T669). 1 × 10^5^ naive and effector CD4^+^ T-cells were re-suspended in 1 mM Ca^2+^ Ringer’s buffer containing 50 µM probenecid (Sigma, Cat# P-8761) and 10 nM TMRE. Cells were added onto a 25 mm poly-L-ornithine-coated coverslip and stained for 30 min at 37 °C under low-light conditions. Measurements were performed at 37 °C, while keeping the dye and the inhibitor concentration consistent. 2 µM CCCP (Sigma, Cat# C2759-100MG) were added towards the end of the measurement as a control stimulus. A fluorescence Zeiss D1 Cell Observer microscope was used to assess TMRE intensity with a 40x/1.3 oil Neofluar objective, Axiocam 702 mono and LED system (Colibri, Zeiss). Images were acquired upon excitation at 555 nm (excitation filter: 550/32 nm) together with a 573 nm dichroic mirror, and 630/92 nm emission filter. Data acquisition was performed with the Zen 2.6 software, data processing and statistics with Microsoft Excel 2019 and plotting with GraphPad Prism 9. Data were analysed by marking only the mitochondrial area of the cells and plotted as mean TMRE fluorescence intensity (normalised to background fluorescence) over time (minutes).

#### JC-1 measurements

Further, ∆Ψm measurements were performed using the lipophilic ratiometric dye JC-1 (Invitrogen, Cat# T3168). To that end, 2 × 10^5^ CD4^+^ T-cells per donor (naive or effector) were stained with 500 nM JC-1 for 20 min at RT under low-light conditions. The cells were left to attach on previously-coated (100 µg/mL of poly-L-ornithine) 25 mm coverslips and measured in 1 mM Ca^2+^ containing Ringer’s buffer. A fluorescence Zeiss D1 Cell Observer microscope was used to perform JC-1 measurements at 37 °C using a 40x/1.3 oil Neofluar objective, Axiocam 702 mono and LED system (Colibri, Zeiss). Images were acquired upon excitation at 555 nm (excitation filter: 550/32 nm) and 485 nm (excitation filter 550/32 nm) together with 573 and 515 nm dichroic mirrors, and 539/25 and 630/92 nm emission filters. Data acquisition was performed with the Zen 2.6 software, data processing and statistics with Microsoft Excel 2019 and plotting with GraphPad Prism 9. Data were analysed by marking only the mitochondrial area of the cells, and plotted as red/green ratio (normalised to background fluorescence) over time (minutes).

### Cytosolic and mitochondrial hydrogen peroxide measurements

Cytosolic and mitochondrial H_2_O_2_ in CD4^+^ T-cells were measured using the ratiometric protein biosensors HyPer3 and HyPer-dMito at 37 °C in Ringer’s buffer solution (1 mM [Ca^2+^]). Measurements were performed on a Zeiss Observer D1 equipped with a 40x/1.3 oil Neofluar objective, Axiocam 702 mono and LED system (Colibri, Zeiss). Images were acquired upon excitation at 420 nm (excitation filter: 420/40 nm) and 505 nm (excitation filter: 500/15 nm) together with a 515 nm dichroic mirror and 539/25 nm emission filter. Data acquisition was performed with the Zen 2.6 software, data processing and statistics with Microsoft Excel 2019, and plotting with GraphPad Prism 9. Data were analysed and plotted as a 505/420 nm ratio (normalised to background fluorescence) over time (minutes).

### Cellular ROS measurements

Global ROS levels in naive and effector T-cells were assessed using the cell-permeable dye 2’,7’-dichlorodihydrofluorescein diacetate (H_2_DCFDA; Thermo Fisher, Cat# D399). T-cells were stained with 1 µM working H_2_DCFDA solution for 40 min at RT in low-light conditions. Next, 200,000 cells were seeded per well in 1 mM CaCl_2_ containing Ringer’s buffer in a black 96-well plate with a clear bottom. Fluorescence levels were measured using the CLARIOstar microplate reader (Ex/Em: 485/535 nm). Data were analysed and plotted as mean H_2_DCFDA fluorescence intensity (normalised to background fluorescence).

### Immunofluorescence imaging of nuclear NFAT1 translocation

Cultured primary human T-cells (1 × 10^5^) were seeded on top of 12 mm glass coverslips (thickness #1.5–0.73 µm) coated with 100 µg/mL of poly-L-ornithine (diluted in ddH_2_O) in the appropriate culture medium. They were left to adhere for at least 3 hrs at 37 °C. Next, the cells were gently washed with 1x DPBS. Thapsigargin (1 µM) was added to half of the prepared coverslips to initiate an increase in _c_[Ca^2+^], thereby NFAT1 translocation to the nucleus, for 20 min in DPBS at RT. Immediately after, the solution was removed and the cells were fixed in 4% PFA for 10 min at RT, protected from light. Upon fixation, the coverslips were washed with 1x DPBS and a blocking step using 1x DPBS + 5% FCS was performed (1 hrs at RT). Cells were stained with NFAT1 antibody (Cell Signaling Technology, Cat# D43B1, 1:100 dilution) overnight at 4 °C. Next, cells were stained with Alexa-Fluor-488-goat anti-rabbit secondary antibody (Thermo Fisher, Cat# A-11008; 1:50 dilution) for 1 hrs at RT (low light conditions). Finally, the coverslips were mounted on glass slides using the Fluoromount-G + DAPI mounting medium (Invitrogen #00-4959-52). Imaging of NFAT translocation was performed on an LSM800 Confocal microscope (Zeiss) equipped with a 63x/1.3 glycerine LCI Plan-Neofluar objective, a 405 nm laser for DAPI (at 2% power), a 488 nm laser for NFAT (at 1.5% power) and 2 MA-PMT. Quantification was performed based on the NFAT intensity signal using the Zen 3.5 software (Zeiss). Cytosolic NFAT signal intensity was quantified by subtracting the nuclear NFAT signal intensity from the total cellular NFAT signal intensity. Additionally, nuclear NFAT signal intensity was quantified as the ratio between the nuclear NFAT signal intensity to the total cellular NFAT signal intensity of one cell. Single cell intensities were quantified and plotted using Microsoft Excel 2019 and GraphPad Prism 9, respectively.

### Confocal microscopy

#### Human CD4^+^ T-cells

To quantify and visualise cell and mitochondrial volumes, naive and effector T-cells were stained with MitoTracker Deep Red (100 nM working dilution) for 15 min and fixed immediately after with 4% PFA solution. The cells were mounted on glass slides using a Fluoromount-G medium (Invitrogen Cat# 00-4958-02). Identical acquisition settings were applied with a Nikon Ti inverted microscope equipped with a 100× 1.49 NA oil immersion objective, a Yokogawa CSU-W1 spinning disk unit and an Andor iXon Ultra 888 EMCCD camera in order to acquire z-stacks of individual naive and effector T-cells. The resulting images were deconvolved with Huygens Professional (v. 23.04). Using pixel classification within the Imaris 10.1.0 software package, the ubiquitously-present, low-intensity MitoTracker Deep Red signal within the cells was used to create the respective surface depicting the individual cell, while the pixel population of high-intensity MitoTracker Deep Red signal was applied to segment the mitochondria. Both naive and effector T-cells were segmented using the same, automated image analysis conditions. The resulting data were organized using KNIME v. 5.1.2 software package (Berthold et al, 2009) and plotted using GraphPad Prism 9. The ratio of mitochondrial-to-total cell volume was used for normalisation purposes in other experiments.

#### Rat CD4^+^ T-cells

Rat effector T-cells transduced with the 2mtScarlet-GCaMP6s construct were fixed in 2% PFA for 5 min at RT and attached to glass slides using cytospin centrifugation (Shandon). After 5 min of staining with DAPI in PBS, cells were mounted on coverslips in Fluoromount medium (Southern Biotech). Confocal microscopy was performed using a Zeiss Laser Scanning Microscope 710 (Zeiss) containing the Zeiss ZEN 2012 software (Zeiss). Fluorophores were excited using 405 nm UV-diode laser for DAPI (at 0.6% power), 488 nm Argon laser for GCaMP6s (at 24% power), a 561 nm DPSS laser for mtScarlet (at 8.5% power). Accordingly, filters were set from 422 to 497 nm for DAPI, 505 to 553 nm for GCaMP6s, and 588 to 634 nm for mtScarlet acquisition. A 40× oil/1.3 NA Plan Apochromat objective (Zeiss) was used. Images were sequentially acquired using a pinhole size of 62 μm for track 1 (GCaMP6s and mtScarlet) and 90 µm for track 2 (DAPI).

### Mitochondrial protein translation-^35^S metabolic labelling

Naive T-cells were activated with beads, according to the protocol outlined previously, for 24, 48 and 72 h, and the siMCUa_KD_ model was generated as described. T-cells (2 × 10^6^) were pelleted and resuspended in 1 mL of serum- and methionine-free medium in a low-binding tube. Cells were then treated with emetine (100 µg/mL, Merck, Cat# 324693) for 10 min at 37 °C, followed by labelling with 20 µL of 200 µCi ^35^S methionine (Hartmann Analytic, Cat# SCM-01) for 60 min. The labelled cells were then harvested, washed with cold 1x PBS and protein concentration was determined using a Bradford assay. 40 µg total protein (with loading dye) was incubated at 37 °C for 15 min in the presence of Benzonase, loaded onto a Tris-Tricine gel and run overnight at 80 V, 25 mA. Protein transfer was performed the following day and the membrane was exposed to phosphoscreens and signals obtained by autoradiography. The 13 mitochondrially-synthesised proteins were normalised to the respective control. Quantification was performed using the Image Studio Lite Version 5.2 software.

### Complex activity assays

Complex I activity in human CD4^+^ T-cells was assessed using the colorimetric Complex I Enzyme Activity Assay Kit (Abcam, Cat# ab109721) according to manufacturer’s instructions. Total T-cell sample protein concentration was determined by a Bradford assay and the concentration was adjusted to 5.5 mg/mL. Optical density (OD) was measured for 30 min in an interval of 30 s, using the Synergy H1 (BioTek) plate reader at 450 nm wavelength. Complex I activity in each well was proportional to the increase in absorbance at OD 450 nm within each well.

Complex IV activity was assessed using the Complex IV Human Enzyme Activity Microplate Assay Kit (Abcam, Cat# ab109909) according to manufacturer’s instructions. Total sample protein concentration was measured with a Bradford assay and adjusted to 5 mg/mL. Absorbance at OD of 550 nm was measured for 2 hrs in an interval of 60 s using the Synergy H1 (BioTek) plate reader. Complex IV activity is measured as the rate of oxidation of cytochrome c.

### T7 endonuclease 1 (T7E1) mismatch detection assay

To confirm and characterise the intended edit of the MCU sequence in the generated stable rat effector CD4^+^ T-cell lines, as well as to control for off-target effects after CRISPR Cas9 editing, the T7E1 assay was performed. Briefly, isolated genomic DNA from the generated cell lines (sgRosa26 Control, sgMCUa_KD_ 1, sgMCUa_KD_ 2, and sgMCUa_KD_ 3) was amplified by PCR using primers specific for the edited regions (refer to Reagents and Tools Table). Following PCR amplification, the products were denatured at 95 °C for 5 min and reannealed using ramping cooling protocol, then incubated with T7 endonuclease (NEB; 1 µL enzyme per 20 µL PCR reaction) at 37 °C for 30 min. The wild-type strands and resulting cleaved fragments were then resolved in an agarose gel through electrophoresis and visualized under UV light.

### In vitro CD4^+^ rat T-cell re-stimulation

Rat CD4^+^ T-cells were re-stimulated by using 96-well culture plates coated with anti-rat CD3 (clone G4.18) and anti-rat CD28 (clone JJ316) antibodies (BD Pharmingen) (1 µg/mL in 1xDPBS, overnight at 4 °C). The cells (70,000 per well) were added in re-stimulation medium. At specific time points (0, 3, 6, and 9 hrs after re-stimulation), the cells were pelleted and collected for RNA isolation.

### Experimental autoimmune encephalomyelitis (EAE) induction in rats

In vivo EAE induction was performed by adoptive T-cell transfer in Lewis rats using the generated control cell line (sgRosa26 Control) and the sgMCUa_KD_ cell lines (sgMCUa_KD_ 1 and sgMCUa_KD_ 2) as described in detail in (Lodygin et al, 2019). EAE scores and animal body weight changes were monitored and plotted using GraphPad Prism 10. All experiments were performed according to the local animal welfare regulations of Göttingen University approved by Lower Saxony Animal Care and Use Committee. Both male and female animals were used.

#### Co-transfer EAE

Scarlet-sgRosa26 Control and Turquoise-sgMCUa_KD_ T-β-syn cells were re-stimulated in parallel, harvested at 48 hrs after stimulation and counted using a Neubauer chamber. T-cells were mixed at 1:1 ratio and injected *i.v*. (3 × 10^6^ T-cells per animal) in Lewis rat recipients. At the day of analysis, EDTA-treated blood was retrieved from the rat heart by cardiac puncture and the animals were perfused with 200 mL ice-cold heparinized PBS before organ explant. PBMCs were isolated from blood by density gradient centrifugation (30 min, 840 × *g*, 20 °C) using Lymphocyte Separation Medium 1077 (PromoCell, Cat #C-44010). Lungs were thoroughly and repeatedly sectioned using a tissue chopper (McIlwain). The homogenized tissue was washed with HEPES-buffered DMEM (Hb-DMEM). Pellets were re-suspended and incubated with 2 mL 0.3% collagenase in PBS for 30 min at 37 °C under constant shaking. Subsequently, the tissue was homogenized using a gentleMACS Dissociator (Miltenyi, Cat# 130-093-235), forced through a cell strainer (40 µm) and washed with Hb-DMEM. The pellet was re-suspended with 5 mL 40% isotonic Percoll and underlaid with 5 mL 70% Percoll (centrifugation at 30 min, 2000 rpm, 4 °C). The leukocyte-enriched interphase was then collected, washed and re-suspended with Hb-DMEM containing 2 mM EDTA. Mediastinal lymph nodes were dissected and passed through a cell strainer (40 µm), washed once with 1x PBS and treated with ACK-buffer for erythrocyte lysis. Brain parenchyma and leptomeninges were passed through a cell strainer (40 µm) and washed once with 1x PBS. Myelin debris was eliminated by Percoll-density gradient centrifugation (30 min, 700 × *g*, 4 °C). The cell pellet was washed in 5 mL of Hb-DMEM, collected by centrifugation and re-suspended in Hb-DMEM containing 2 mM EDTA.

Cell suspensions were stained with anti-CD134-AF647 (Clone OX40, BD Biosciences) and anti-CD25-BV750 (Clone OX39, Biolegend) (dilution 1:300) for 20 min on ice. Cell counts were analysed on a CytoFLEX flow cytometer operated with CytExpert software (Beckmann Coulter).

### Statistical analyses

Raw data analyses were performed using Microsoft Excel 2019 and GraphPad Prism 9. Results were plotted using GraphPad Prism v9 or v10 and are given as mean ± SD or SEM (indicated in the figure legend), unless otherwise specified. Statistical significance was determined by a two-tailed paired or unpaired Student’s t-test, and by ordinary one-way-ANOVA for multiple group comparisons. The two-tailed Wilcoxon signed-rank test, the Mann–Whitney U test, or the Welch’s test were used when unequal variance was assumed, as indicated in each figure legend. Other statistical tests performed are explained in detail in specific figure legends. Significance (*p*) values are marked with * for *p* ≤ 0.05, ** for *p* ≤ 0.01, *** for *p* ≤ 0.001, **** for *p* ≤ 0.0001, and ns for not significant.

## Supplementary information

Peer Review File

Appendix

Source data Fig. 1

Source data Fig. 2

Source data Fig. 3

Source data Fig. 4

Source data Fig. 5

Source data Fig. 6

Source data Fig. 7

Appendix and EV Figures Source Data

Expanded View Figures

## Data Availability

The mass spectrometry proteomics data have been deposited to the ProteomeXchange Consortium via the PRIDE (Perez-Riverol et al, 2021) partner repository with the dataset identifiers PXD048682 (human CD4^+^ T-cells) and PXD048650 (rat CD4^+^ T-cells). RNA-sequencing data has been deposited at Gene Expression Omnibus (GEO) archive under the accession number GSE253049. The source data of this paper are collected in the following database record: biostudies:S-SCDT-10_1038-S44319-024-00313-4.
