# Supplementary material for: Mitochondrial calcium uniporter complex controls T-cell-mediated immune responses
Source: EMBO Rep. 2024 Dec 2;26(2):407–42. doi: 10.1038/s44319-024-00313-4 (PMC11772621; doi:10.1038/s44319-024-00313-4)
Supplement: Supplementary file 2 — Appendix [file 44319_2024_313_MOESM2_ESM.pdf]

MITOCHONDRIAL CALCIUM UNIPORTER COMPLEX CONTROLS T-CELL-MEDIATED IMMUNE RESPONSES

Shumanska et al.

APPENDIX

TABLE OF CONTENTS

APPENDIX FIGURES

Appendix Figure S1. Gating strategy for flow cytometry (related to Fig. EV1A)................. 2

Appendix Figure S2. Characterisation of MCU complex and OXPHOS-related proteins upon T-cell activation (related to Fig. 2).. 3

Appendix Figure S3. Influence of $MCUa_{KD}$ on mitochondrial function................................. 4

Appendix Figure S4. Effects of MCUa overexpression on [$_{m}Ca^{2+}$] and T-cell function......... 6

Appendix Figure S5. Published proteomics datasets confirm changes in MCU expression upon T-cell activation………………………………………………………………………....8

APPENDIX FIGURES

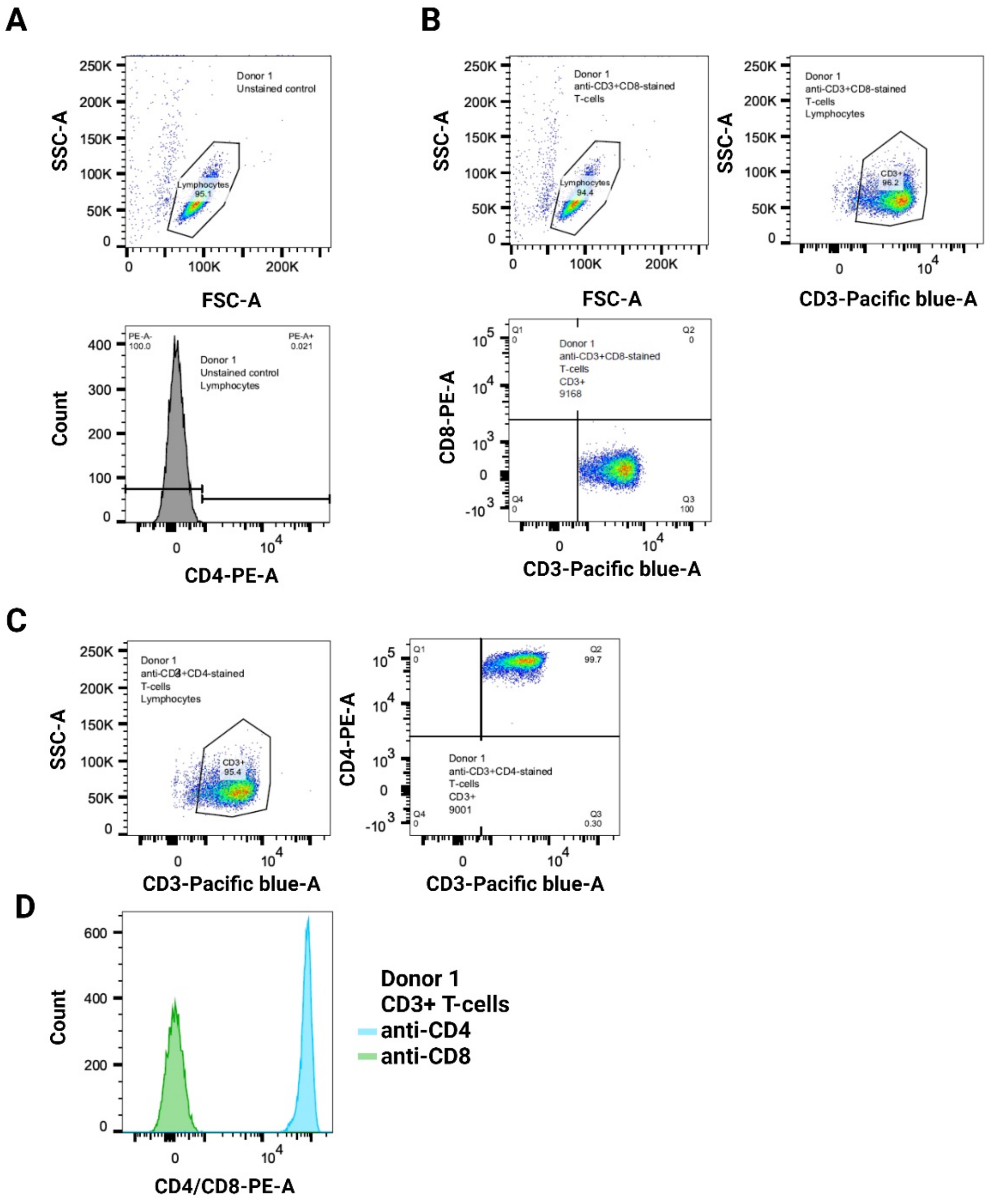

Appendix Figure S1. Gating strategy for flow cytometry (related to Fig. EV1A).

(**A**) Gating strategy for inclusion of lymphocytes and exclusion of debris and doublets in an unstained control sample from one representative donor. (**B**) Gating strategy for inclusion of only T-cells based on CD3 marker expression. 100% of the cells were CD3 positive and CD8 negative (quadrant 3). (**C**) 99% of the gated T-cells were both CD3 and CD4 positive (quadrant 2). (**D**)

Histogram representing the data from **B** and **C**. Both donor samples shown in Fig. EV1A were processed and gated in the same way as represented here by using FlowJo v10.9.0.

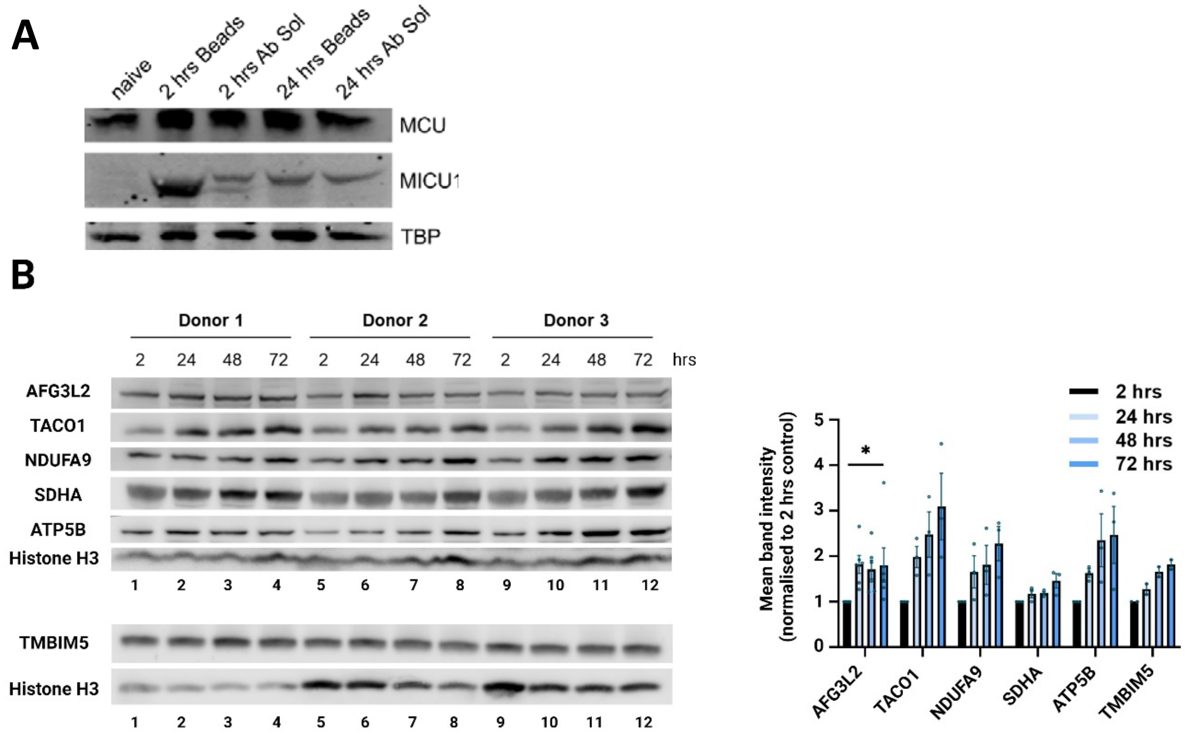

Appendix Figure S2. Characterisation of MCU complex and OXPHOS-related proteins upon T-cell activation (related to Fig. 2)

(**A**) Protein abundance of MCU and MICU1 in human naive and effector T-cells from one donor stimulated with anti-human CD3/CD28-coated beads or anti-CD3/CD28 antibody solution for 2 or 24 hours. (**B**) Activation of naive $CD4^{+}$ T-cells caused upregulation of multiple proteins related to oxidative phosphorylation. The figure and quantification show 3 different donors/biological replicates, activated for 2, 24, 48 and 72 hours with anti-CD3/CD28 beads. For quantification, data are normalised to the 2 hours control samples, respectively for each donor. Data are represented as mean ± SEM. Single donors are given as single points. * - $p \leq 0.05$, assessed by two-tailed paired Student's t-test.

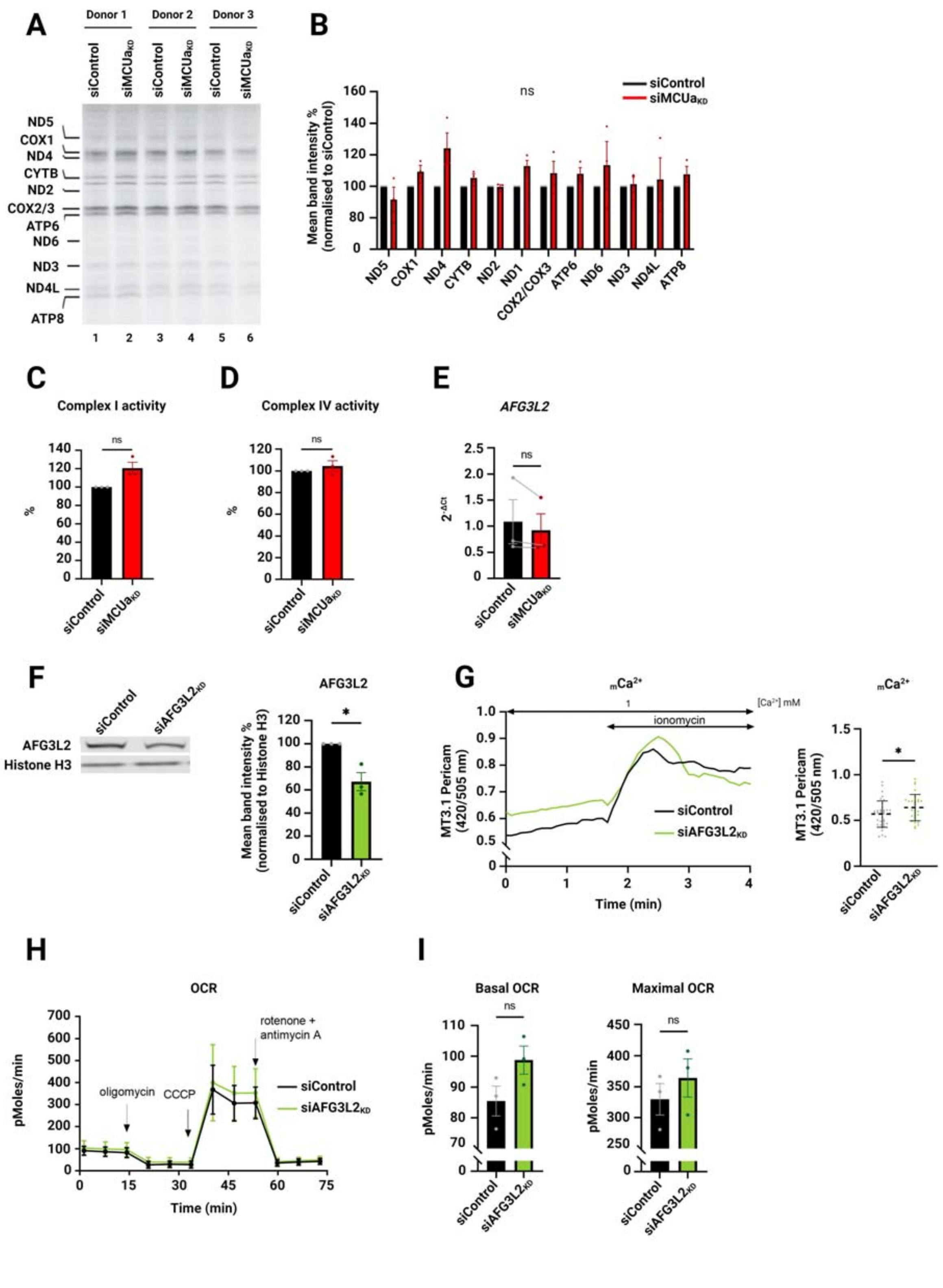
A
Donor 1
Donor 2
Donor 3
siControl
siMCUaKD
ND5
COX1
ND4
CYTB
ND2
COX2/3
ATP6
ND6
ND3
ND4L
ATP8
1 2 3 4 5 6
B
ns
siControl
siMCUaKD
Mean band intensity % (normalised to siControl)
ND5 COX1 ND4 CYTB ND2 ND1 COX2/COX3 ATP6 ND6 ND3 ND4L ATP8
C
Complex I activity
ns
%
siControl
siMCUaKD
D
Complex IV activity
ns
%
E
AFG3L2
ns
2-ΔCt
F
siControl
siAFG3L2KD
AFG3L2
Histone H3
AFG3L2
*
Mean band intensity % (normalised to Histone H3)
G
mCa2+
1
[Ca2+] mM
ionomycin
MT3.1 Pericam (420/505 nm)
siControl
siAFG3L2KD
Time (min)
mCa2+
*
H
OCR
pMoles/min
oligomycin
CCCP
rotenone + antimycin A
siControl
siAFG3L2KD
Time (min)
I
Basal OCR
Maximal OCR
ns
pMoles/min

Appendix Figure S3. Influence of $MCUa_{KD}$ on mitochondrial function

(**A-B**) Mitochondrial protein translation (quantifications represent an average of the 3 different donors/biological replicates shown on the image, (percent of control). Data are shown as mean ± SEM. Single donors are given as single points. Statistical assessment by two-tailed paired Student's t-test. (**C**) No changes in complex I and (**D**) complex IV activity in $siMCUa_{KD}$ cells compared to control. Panel shows the quantified means ± SEM as % of control from 3 different donors (single points represent individual donors). ns – not significant, assessed by Wilcoxon signed-rank test. (**E**) No difference in *AFG3L2* mRNA expression upon MCUa knockdown. The quantification shows the mean of 3 different donors/biological replicates. ns - not significant, assessed by two-tailed paired Student's t-test. (**F**) An average of 50% AFG3L2 knockdown was observed on protein level. The quantification shows the mean ± SEM of 3 separate donors/ biological replicates, normalised to Histone H3 (percent of control). Single donors are given as single points. * - p <0.05 (p=0.0523), assessed by two-tailed paired Student's t-test. (**G**) Resting $_{m}Ca^{2+}$ was increased upon transient downregulation of AFG3L2, with no changes in $_{m}Ca^{2+}$ uptake upon ionomycin (4 µM) addition. Traces and violin plots show a mean ± SD of 36 siControl and 30 $siAFG3L2_{KD}$ $CD4^{+}$ T-cells (biological replicates) from 2 healthy donors. Cells are given as single points. * - p ≤0.05 (p=0.0512), assessed by two-tailed unpaired Student's t-test. (**H-I**) Partial AFG3L2 downregulation did not affect mitochondrial respiration in $CD4^{+}$ T-cells. Basal and maximal OCR represent a mean ± SEM of 3 different donors/biological replicates (given as single points). ns – not significant, assessed by two-tailed unpaired Student's t-test.

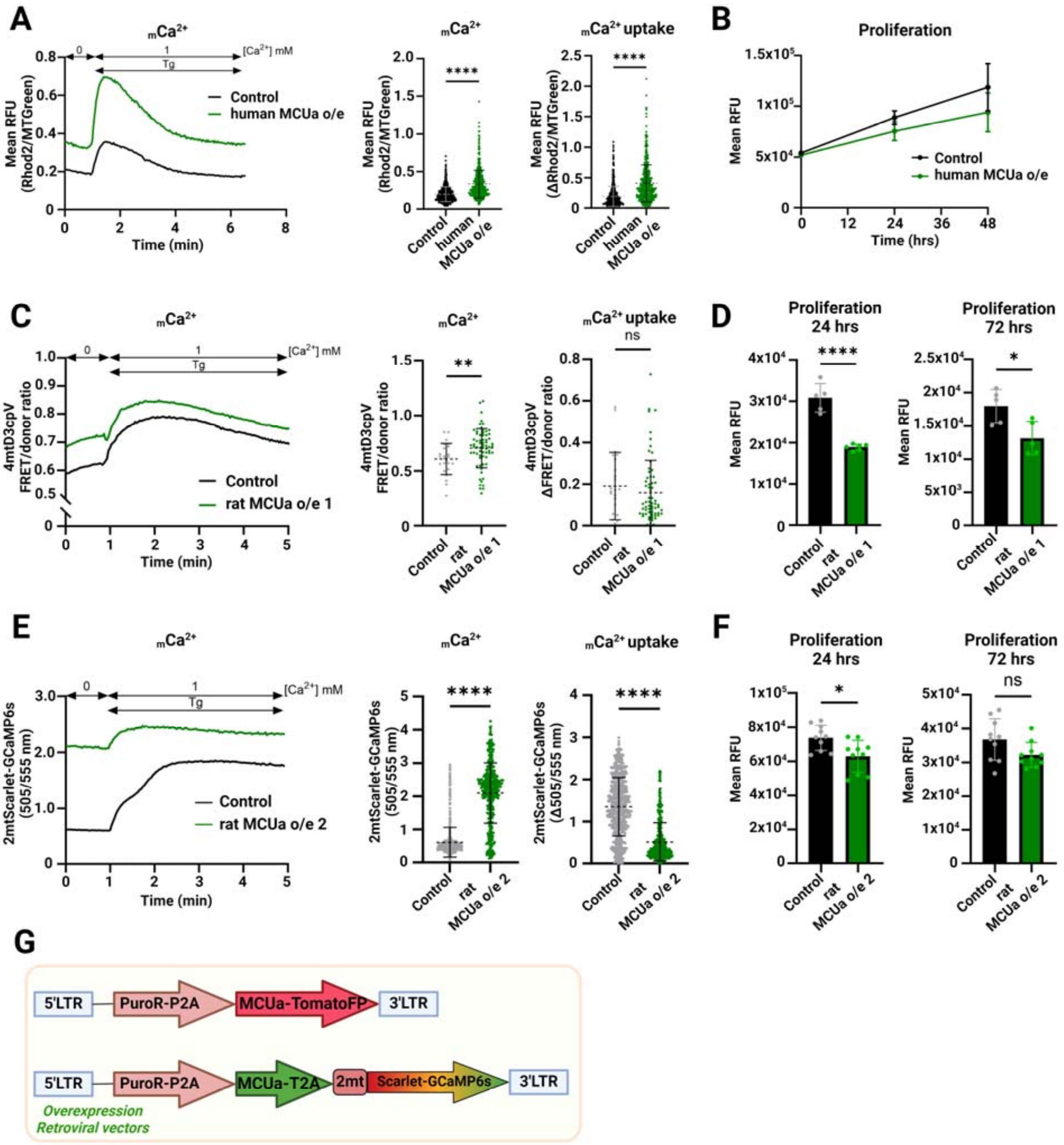

Appendix Figure S4. Effects of MCUa overexpression on [$_m$Ca^{2+}] and T-cell function.

(**A**) $_m$Ca^{2+} levels measured in primary human control and MCUa-overexpression effector T-cells by co-staining the cells with the $_m$Ca^{2+}-sensitive dye Rhod2 and Mito Tracker Green. Traces and quantification plots show an average of 762 control and 499 MCUa o/e cells/ biological replicates from 2 different donors. Cell stimulation was achieved by addition of 1 µM thapsigargin at the indicated time point. The quantification/violin plots show the mean ± SD

basal $_{m}Ca^{2+}$ levels and mitochondrial Ca^{2+} uptake quantified as the ratio of the mean Rhod2 RFU and the mean MT Green RFU. Single cells are given as single points. ****- p <0.0001, assessed by two-tailed unpaired Student's t-test. RFU = relative fluorescence units; MT Green = Mito Tracker Green. (**B**) T-cell proliferation measured in primary human control and MCUa-overexpressing effector T-cells (human MCUa o/e). The traces represent mean ± SEM of 4 different donors/biological replicates. RFU=Relative fluorescence units. (**C**) $_{m}Ca^{2+}$ levels measured in rat control and MCUa-overexpression effector T-cells (rat MCUa o/e 1) by using 4mtD3cpV. Traces and quantification plots show an average of 24 control and 66 MCUa o/e 1 cells/ biological replicates. Cell stimulation was achieved by addition of 1 μM thapsigargin at the indicated time point. The quantification/violin plots show the mean ± SD basal $_{m}Ca^{2+}$ levels and mitochondrial Ca^{2+} uptake. Single cells are given as single points. **-p <0.01; ns – not significant, assessed by Mann–Whitney U test. (**D**) Control and MCUa-overexpressing rat T-cell proliferation at 24- and 72-hours following seeding. Quantifications show a mean ± SEM of 5 separate biological replicates given as single points. **** - p <0.0001; *- p ≤0.05, assessed by two-tailed unpaired Student's t-test. RFU=Relative fluorescence units. (**E**) $_{m}Ca^{2+}$ levels measured in rat control and MCUa-overexpressing effector T-cells (cell line 2) by using the stably-expressed 2mtScarlet-GCaMP6S biosensor. Traces and quantification plots show an average of 631 control and 425 MCUa o/e 2 cells/biological replicates. Cell stimulation was achieved by addition of 1 μM thapsigargin at the indicated time point. The quantification/violin plots show the mean ± SD basal $_{m}Ca^{2+}$ levels and mitochondrial Ca^{2+} uptake. Single cells are given as single points. ****- p <0.0001, assessed by two-tailed unpaired Student's t-test. (**F**) Control and MCUa-overexpressing (MCU o/e 2) rat T-cell proliferation at 24- and 72-hours following seeding. Quantifications show a mean ± SEM of 10 separate biological replicates given as single points. *- p ≤0.05; ns – not significant, assessed by two-tailed unpaired Student's t-test. RFU=Relative fluorescence units. (**G**) The outline of retroviral constructs used to establish control and MCUa overexpression rat effector T-cell lines. LTR – long terminal repeats; PuroR – puromycin resistance gene; U6p – U6 promoter; 2mt – tandem mitochondrial localization signals (Image created by using BioRender).

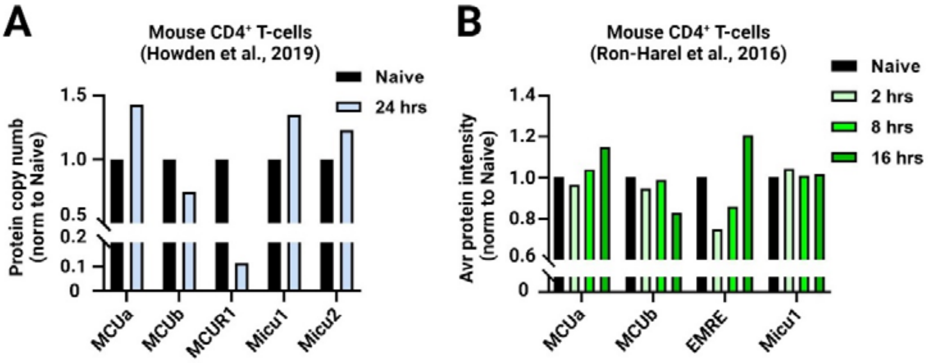

Appendix Figure S5. Published proteomics datasets confirm changes in MCU expression upon T-cell activation.

Published proteomics data on murine $CD4^+$ T-cell activation. MCU complex expression was extracted from online repositories. Data ref: Howden *et al*, (2019) (**A**) performed proteomic analyses with naive and 24 hours-activated mouse $CD4^+$ T-cells (3 technical replicates per condition). Data ref: Ron-Harel *et al,* (2016) (**B**) performed proteomic analyses with naive, 2 hours-activated, 8 hours-activated, and 16 hours-activated mouse $CD4^+$ T-cells (2 technical replicates per condition). MCU complex expression extracted from each study showed similar outcomes; MCUa and MICU1 are upregulated in activated T-cells, whereas MCUb is downregulated.
