# Supplementary material for: Mitochondrial calcium uniporter complex controls T-cell-mediated immune responses
Source: EMBO Rep. 2024 Dec 2;26(2):407–42. doi: 10.1038/s44319-024-00313-4 (PMC11772621; doi:10.1038/s44319-024-00313-4)
Supplement: Supplementary file 4 — Source data Fig. 2 [file 44319_2024_313_MOESM4_ESM.zip › 2G/_35S_raw data/35S labeling-raw image-Donors 2 and 3-labelled.pptx]

## Slide 1
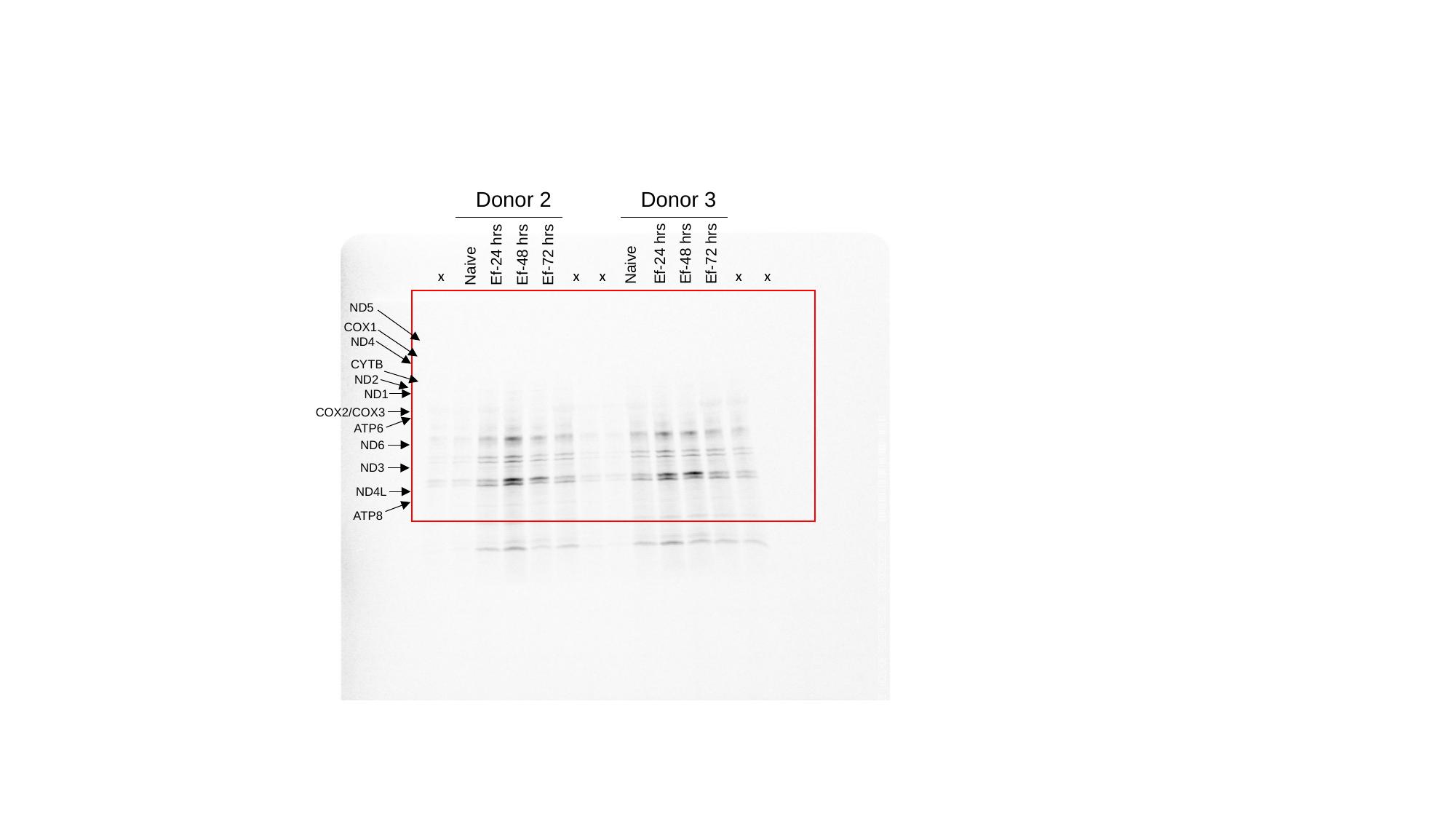

Donor 2
Donor 3
Ef-24 hrs
Ef-72 hrs
Ef-48 hrs
Ef-24 hrs
Ef-72 hrs
Ef-48 hrs
Naive
Naive
x
x
x
x
x
ND5
COX1
ND4
CYTB
ND2
ND1
COX2/COX3
ATP6
ND6
ND3
ND4L
ATP8

## Slide 2
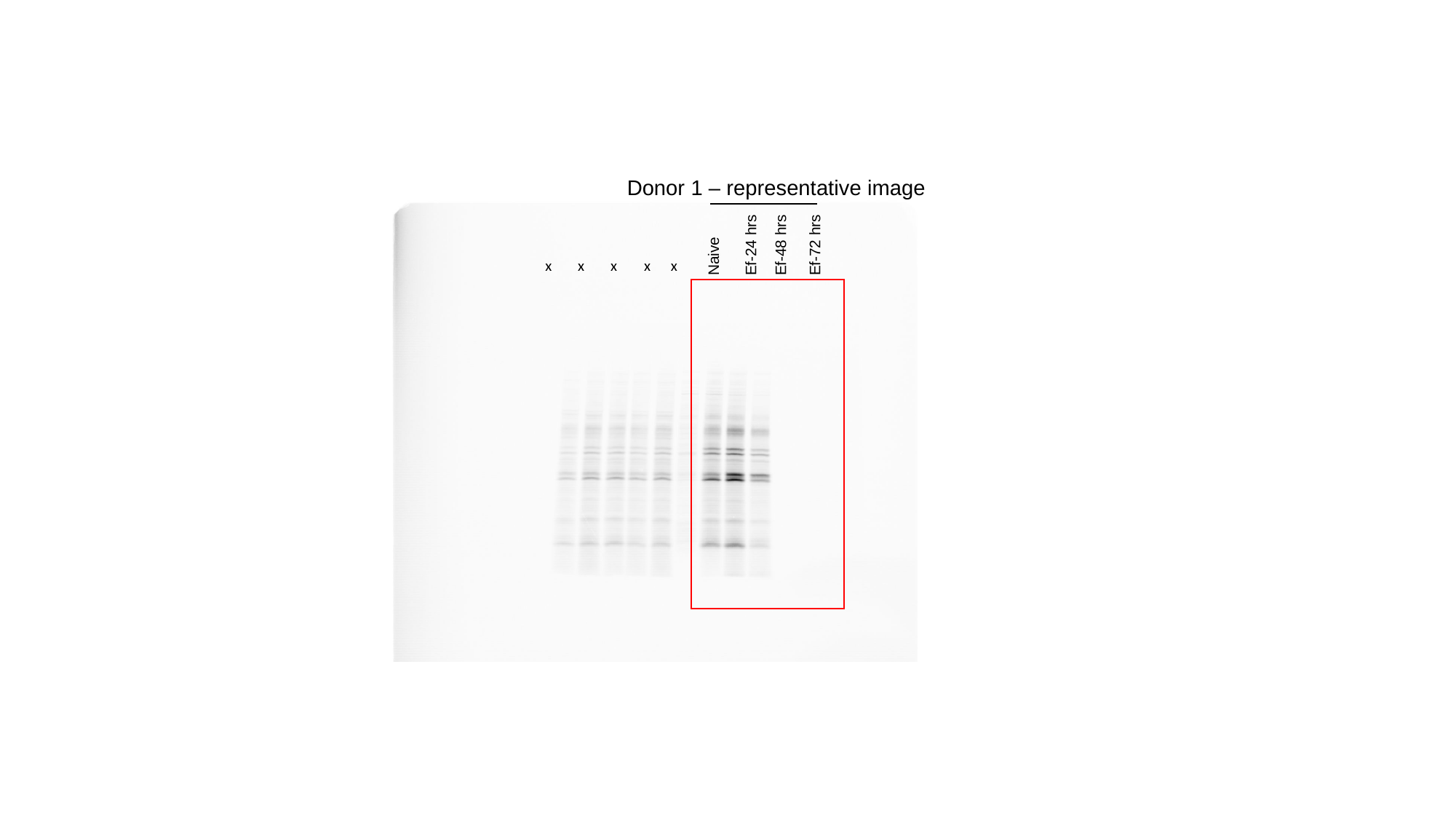

Donor 1 – representative image
Ef-24 hrs
Ef-72 hrs
Ef-48 hrs
Naive
x
x
x
x
x
