# Supplementary figures and images for: Mitochondrial calcium uniporter complex controls T-cell-mediated immune responses
Source: EMBO Rep. 2024 Dec 2;26(2):407–42. doi: 10.1038/s44319-024-00313-4 (PMC11772621; doi:10.1038/s44319-024-00313-4)

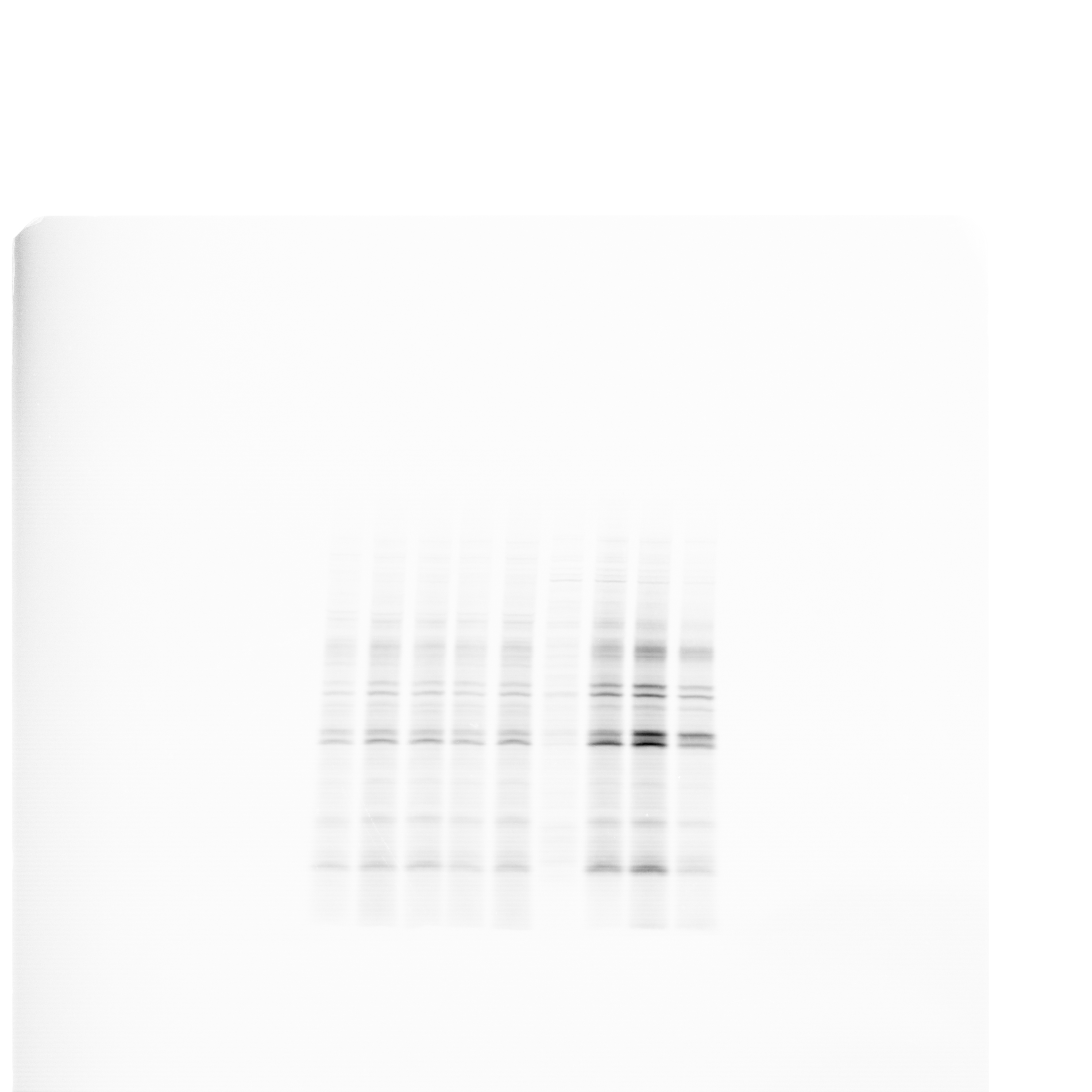

Supplement: Supplementary file 4 — Source data Fig. 2 [file 44319_2024_313_MOESM4_ESM.zip › 2G/_35S_raw data/35S labeling-raw data-Donor 1.tif]

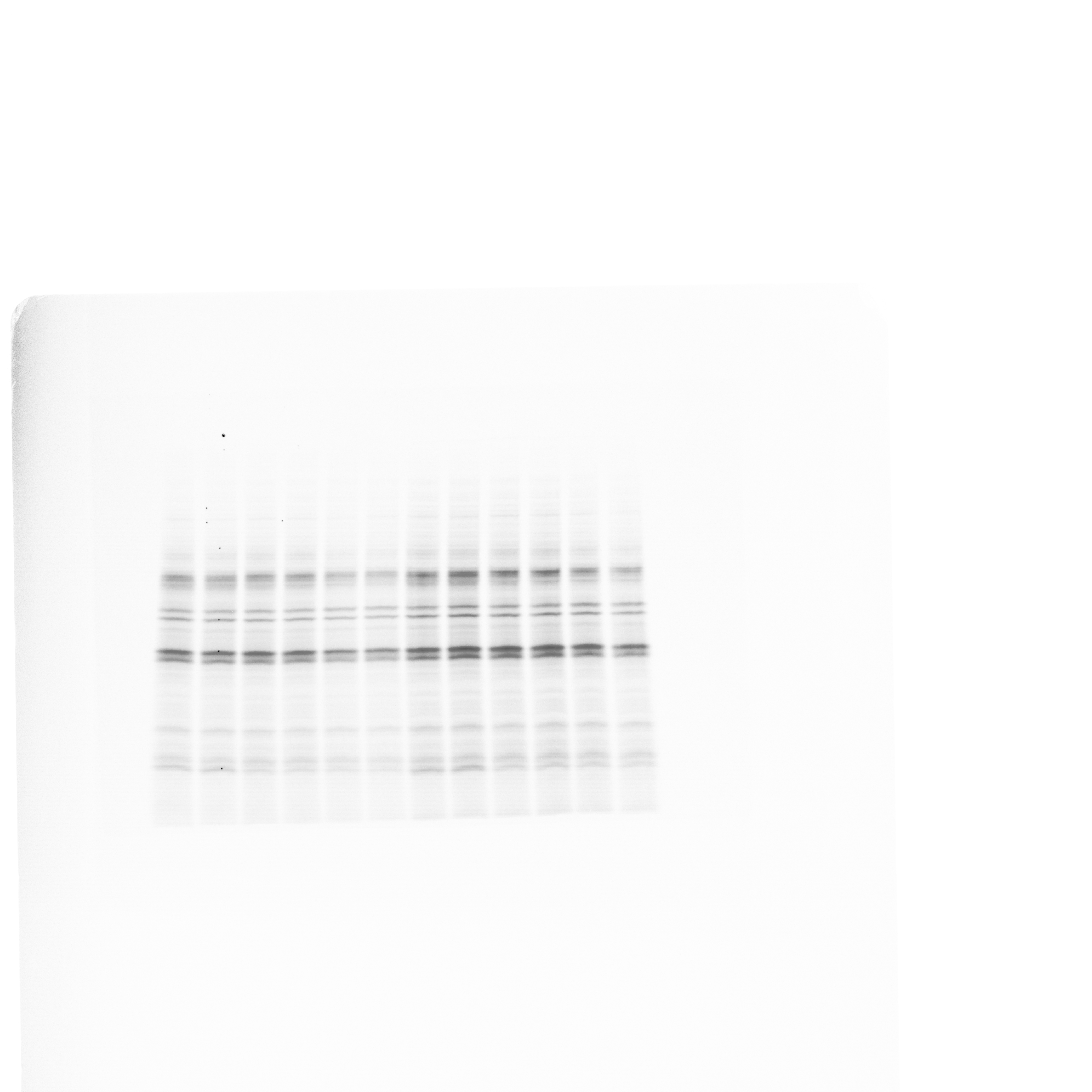

Supplement: Supplementary file 4 — Source data Fig. 2 [file 44319_2024_313_MOESM4_ESM.zip › 2G/_35S_raw data/35S labeling-raw data-Donors 2 and 3.tif]

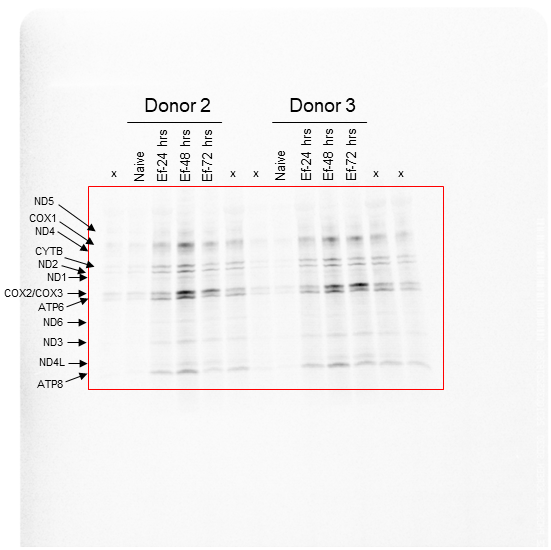

Supplement: Supplementary file 4 — Source data Fig. 2 [file 44319_2024_313_MOESM4_ESM.zip › 2G/_35S_raw data/35S labeling-raw image-Donors 2 and 3-labelled.tif]

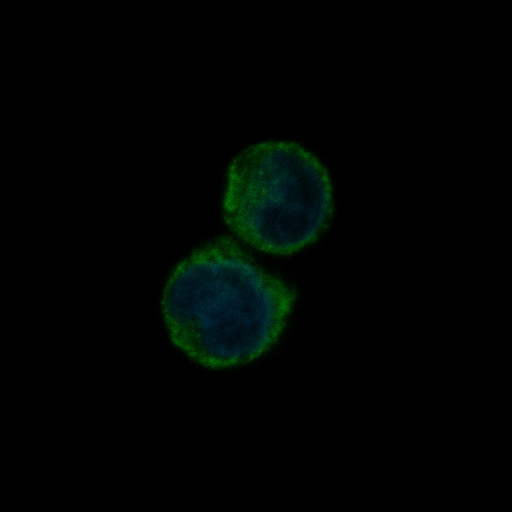

Supplement: Supplementary file 6 — Source data Fig. 4 [file 44319_2024_313_MOESM6_ESM.zip › 4I/Donor2-siControl-no treatment-original/Donor2-siControl-no treatment-5-original_c1-2.jpg]

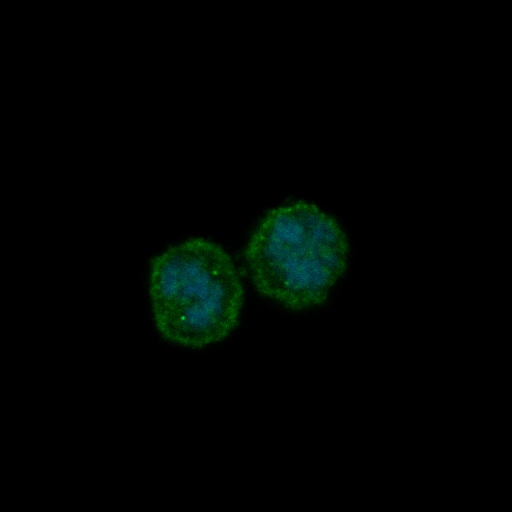

Supplement: Supplementary file 6 — Source data Fig. 4 [file 44319_2024_313_MOESM6_ESM.zip › 4I/Donor2-siControl-TG-original/Donor2-siControl-TG-original_c1-2.jpg]

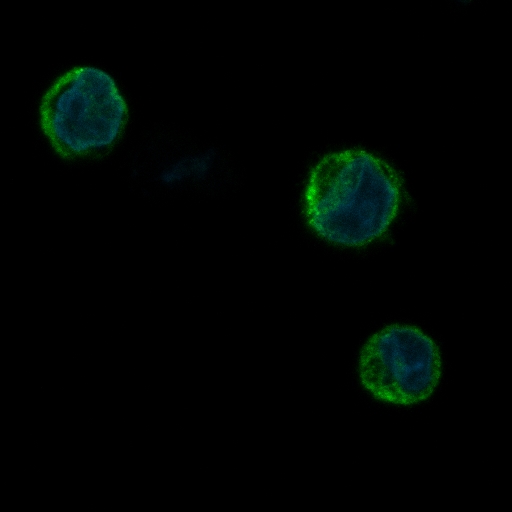

Supplement: Supplementary file 6 — Source data Fig. 4 [file 44319_2024_313_MOESM6_ESM.zip › 4I/Donor2-siMCU-no treatment-original/Donor2-siMCU-no treatment-original_c1-2.jpg]

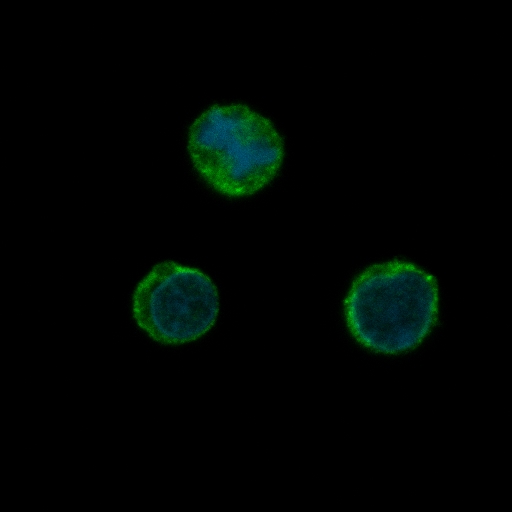

Supplement: Supplementary file 6 — Source data Fig. 4 [file 44319_2024_313_MOESM6_ESM.zip › 4I/Donor2-siMCU-TG-original/Donor2-siMCU-TG-original_c1-2.jpg]

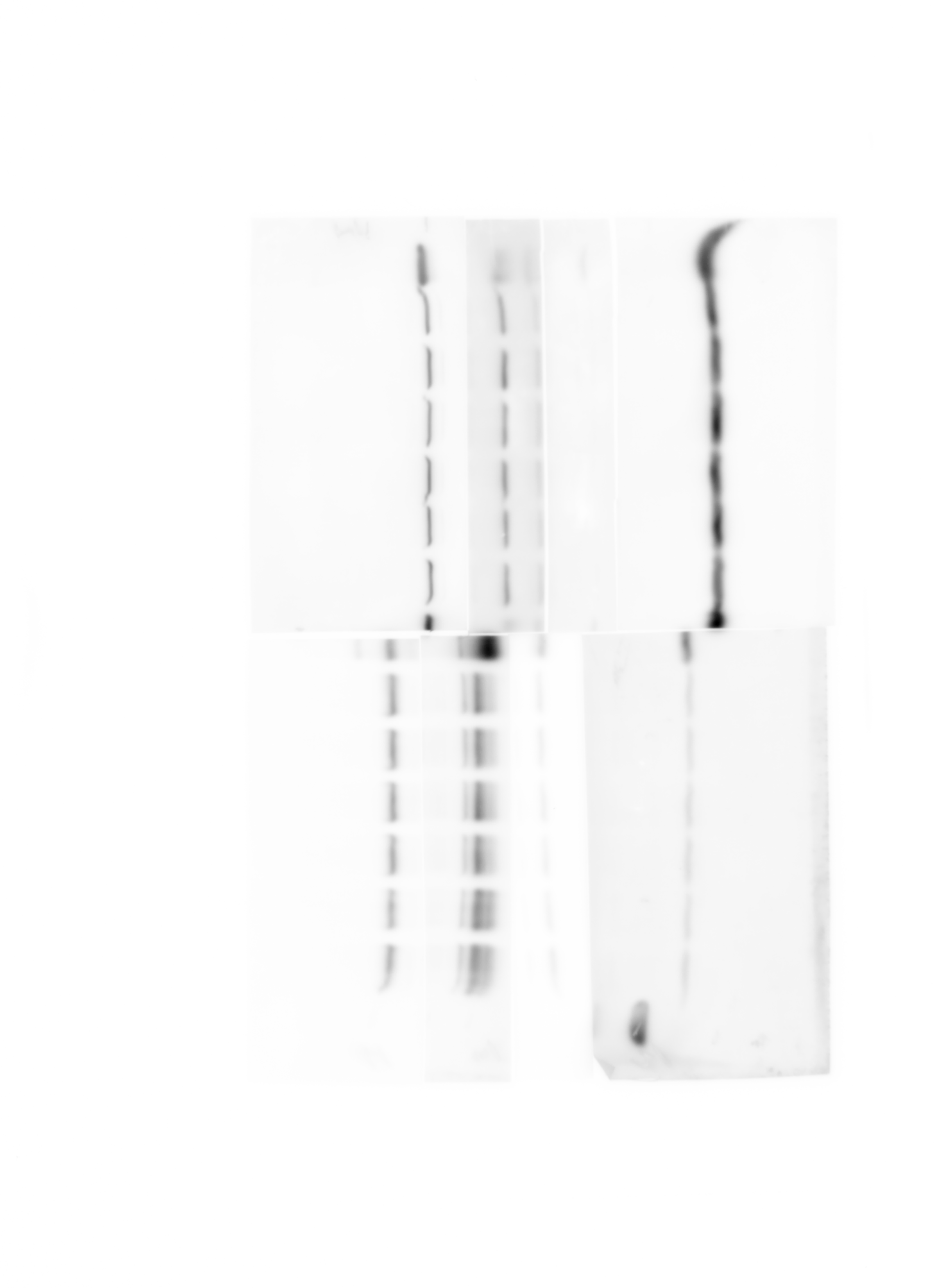

Supplement: Supplementary file 7 — Source data Fig. 5 [file 44319_2024_313_MOESM7_ESM.zip › 5D/1st expo gel1 20210506_174125-01_Ch_Chemi_COX6A.tif]

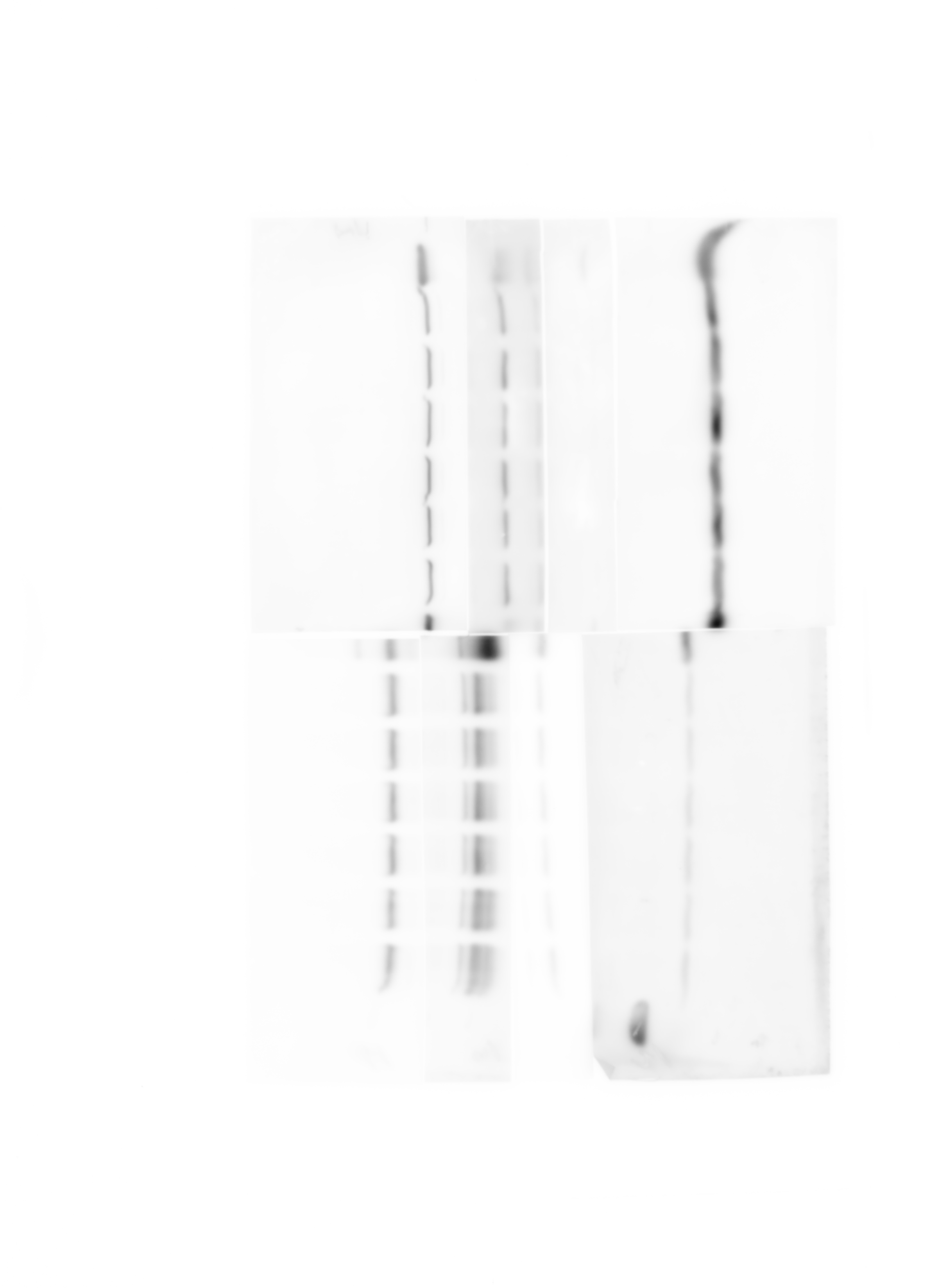

Supplement: Supplementary file 7 — Source data Fig. 5 [file 44319_2024_313_MOESM7_ESM.zip › 5D/1st expo gel1 20210506_174125-03_Ch_Chemi_SDHA.tif]

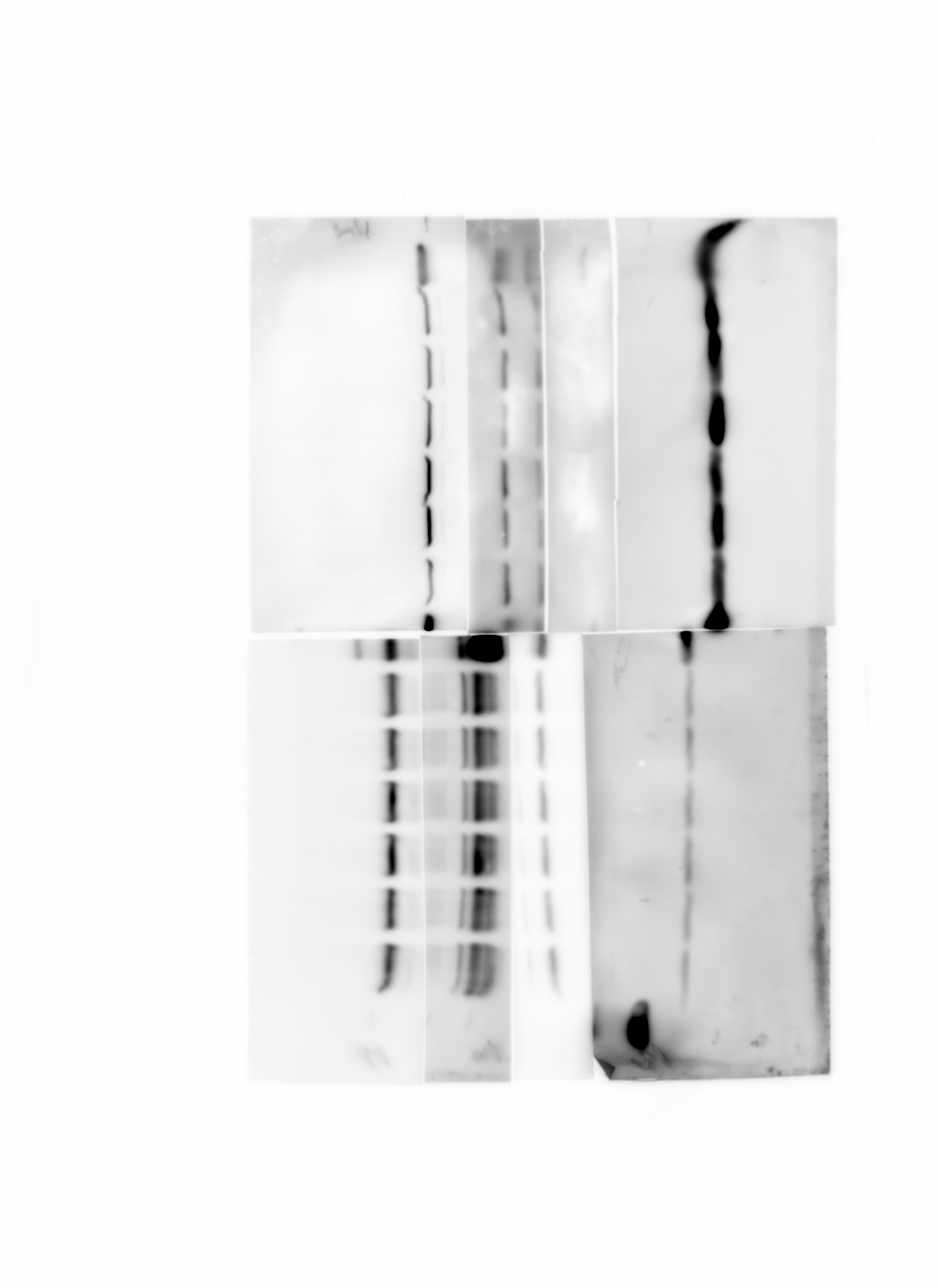

Supplement: Supplementary file 7 — Source data Fig. 5 [file 44319_2024_313_MOESM7_ESM.zip › 5D/1st expo gel1 20210506_174125-20_Ch_Chemi_COX1_TACO1_MITRAC12_ATP5B_uL1m_uL10m_.tif]

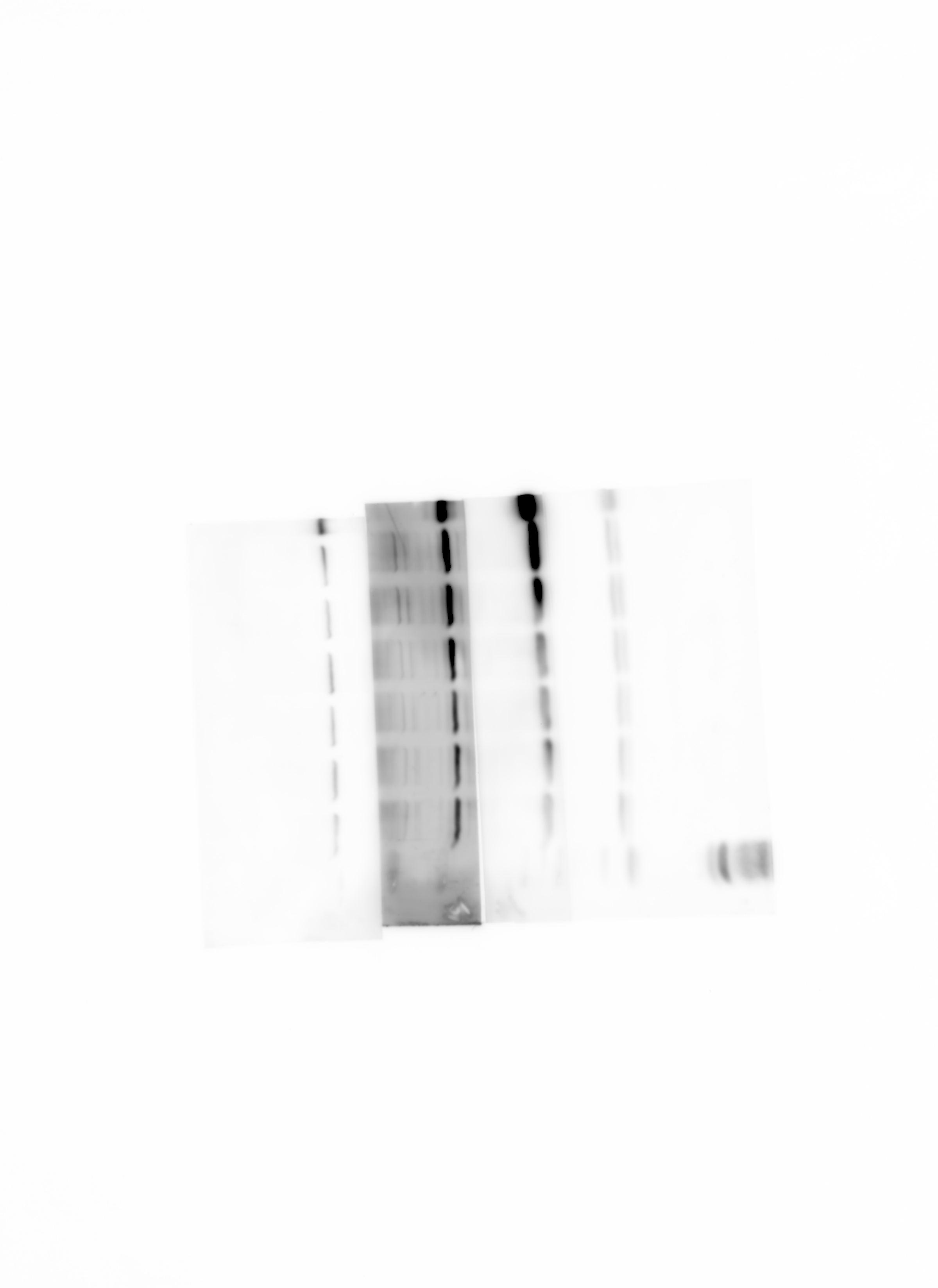

Supplement: Supplementary file 7 — Source data Fig. 5 [file 44319_2024_313_MOESM7_ESM.zip › 5D/1st expo gel2 20210506_175218-20_Ch_Chemi_NDUFA9_uS14m_AFG3L2.tif]
