# Supplementary material for: Mitochondrial calcium uniporter complex controls T-cell-mediated immune responses
Source: EMBO Rep. 2024 Dec 2;26(2):407–42. doi: 10.1038/s44319-024-00313-4 (PMC11772621; doi:10.1038/s44319-024-00313-4)
Supplement: Supplementary file 6 — Source data Fig. 4 [file 44319_2024_313_MOESM6_ESM.zip › 4I/Confocal origical images-raw data.pptx]

## Slide 1
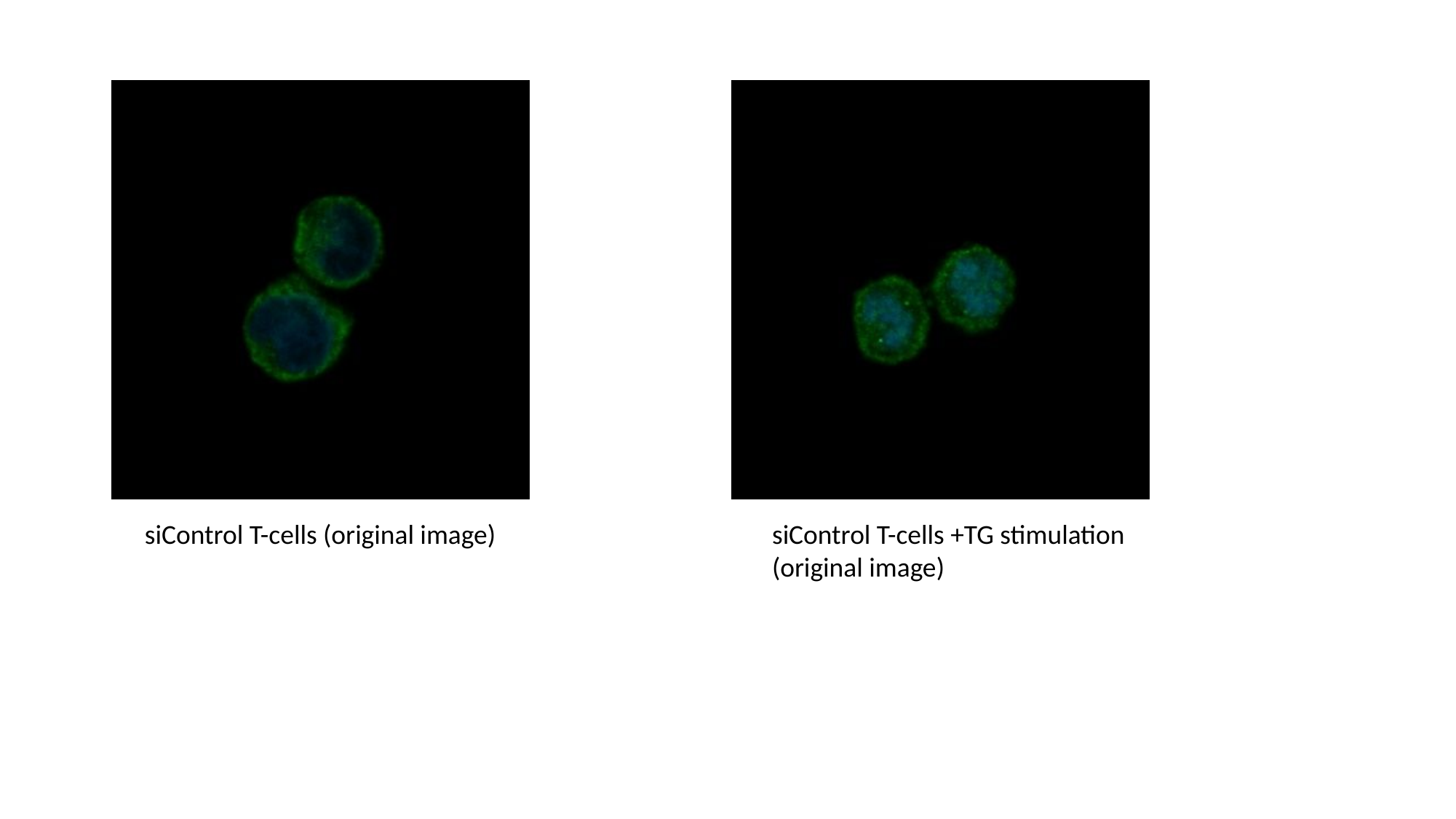

siControl T-cells (original image)
siControl T-cells +TG stimulation
(original image)

## Slide 2
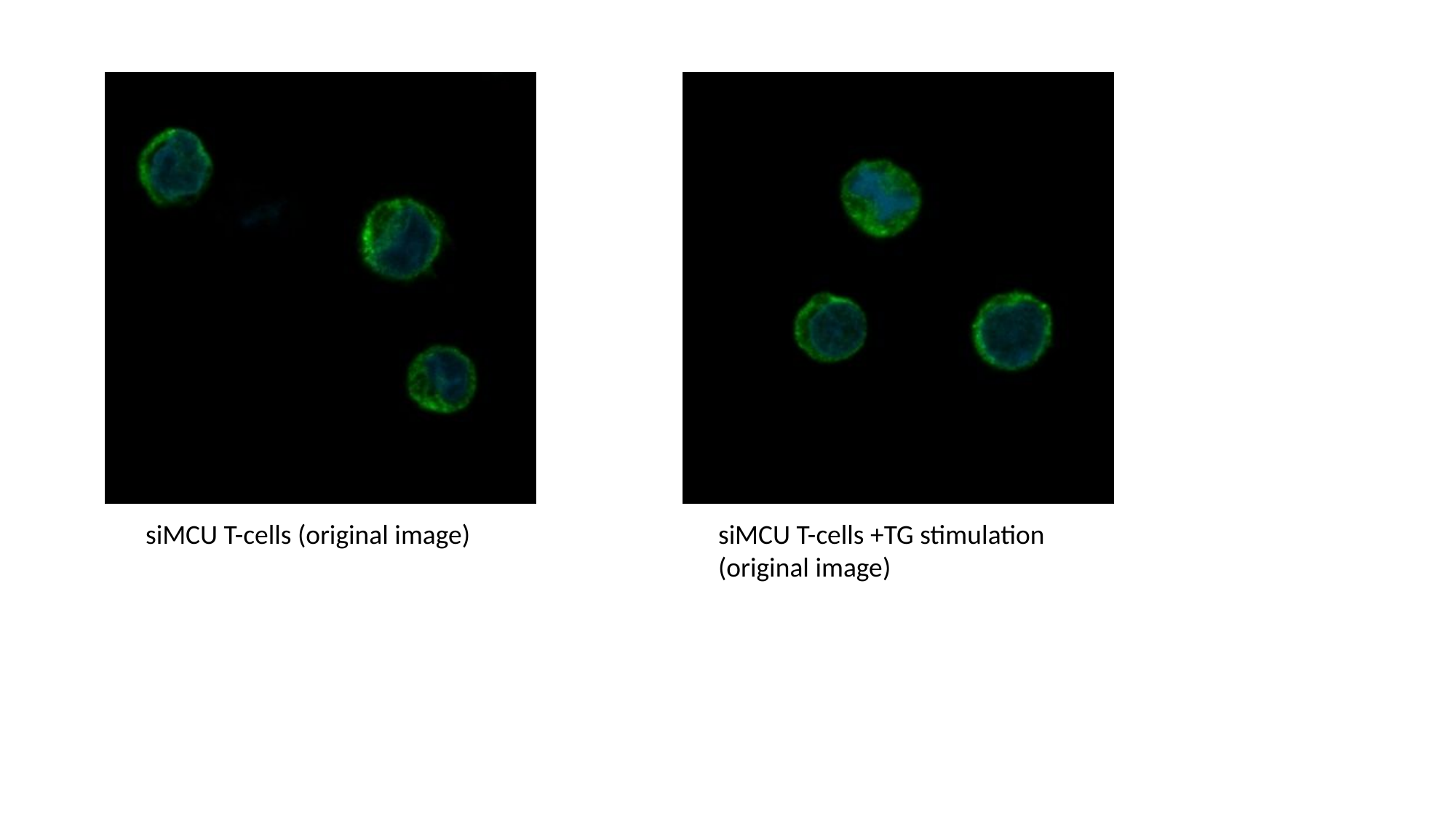

siMCU T-cells (original image)
siMCU T-cells +TG stimulation
(original image)
