# Supplementary material for: Mitochondrial calcium uniporter complex controls T-cell-mediated immune responses
Source: EMBO Rep. 2024 Dec 2;26(2):407–42. doi: 10.1038/s44319-024-00313-4 (PMC11772621; doi:10.1038/s44319-024-00313-4)
Supplement: Supplementary file 7 — Source data Fig. 5 [file 44319_2024_313_MOESM7_ESM.zip › 5D/Figure 5-Panel D-raw blot images-labeled.pptx]

## Slide 1
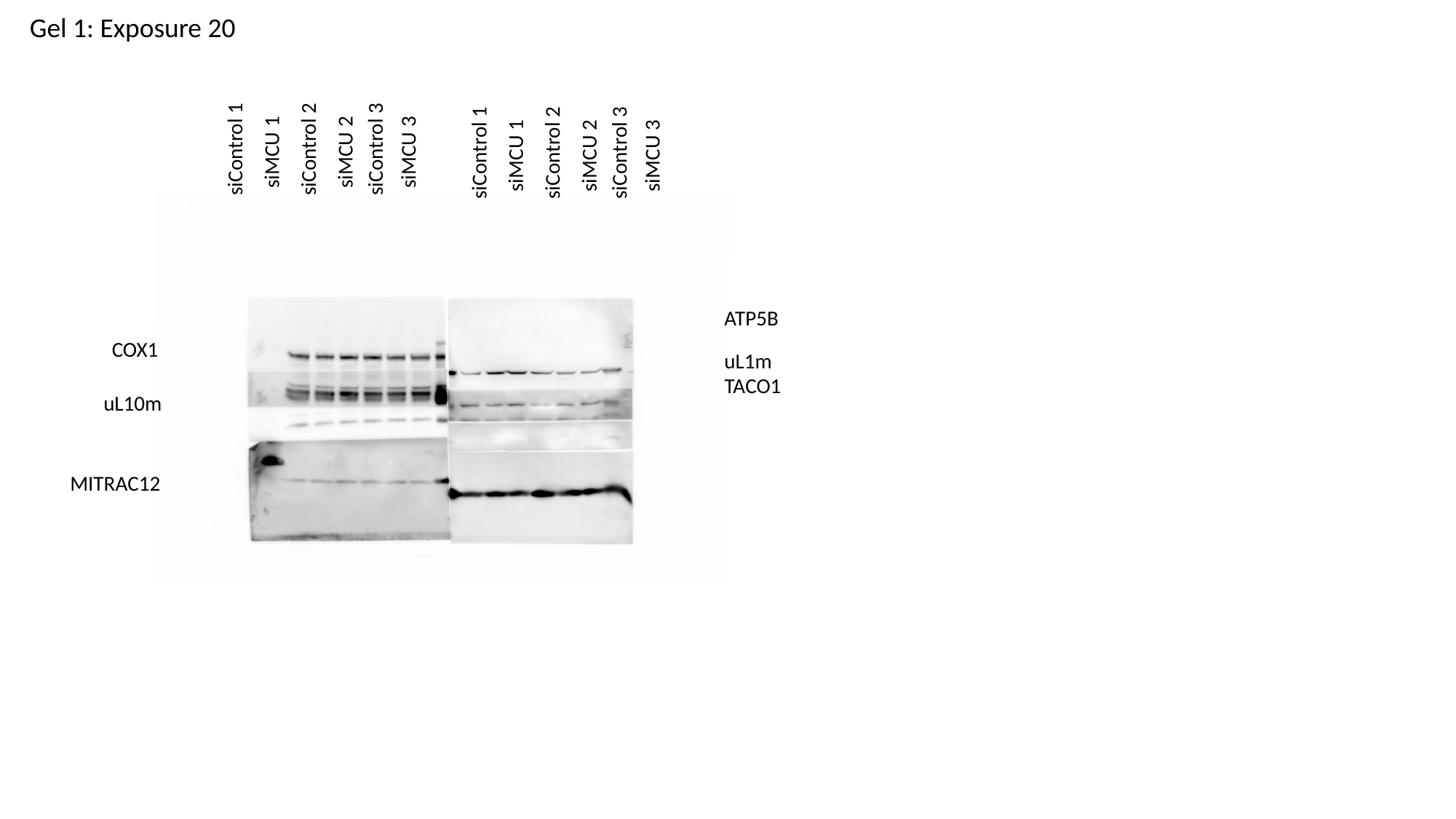

Gel 1: Exposure 20
siControl 1
siControl 2
siControl 3
siMCU 1
siMCU 2
siMCU 3
siControl 1
siControl 2
siControl 3
siMCU 1
siMCU 2
siMCU 3
ATP5B
COX1
uL1m
TACO1
uL10m
MITRAC12

## Slide 2
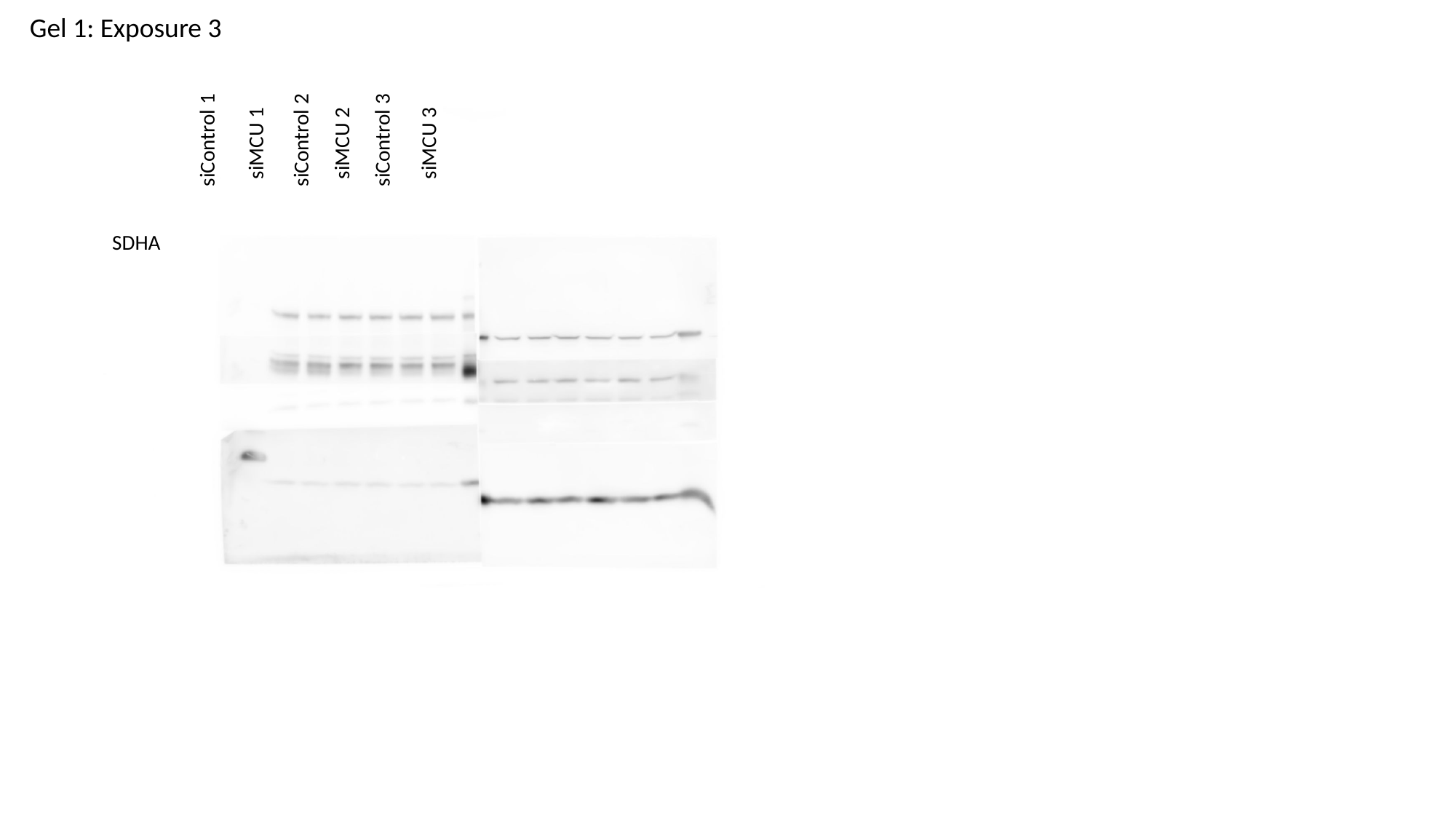

Gel 1: Exposure 3
siControl 1
siControl 2
siControl 3
siMCU 1
siMCU 2
siMCU 3
SDHA

## Slide 3
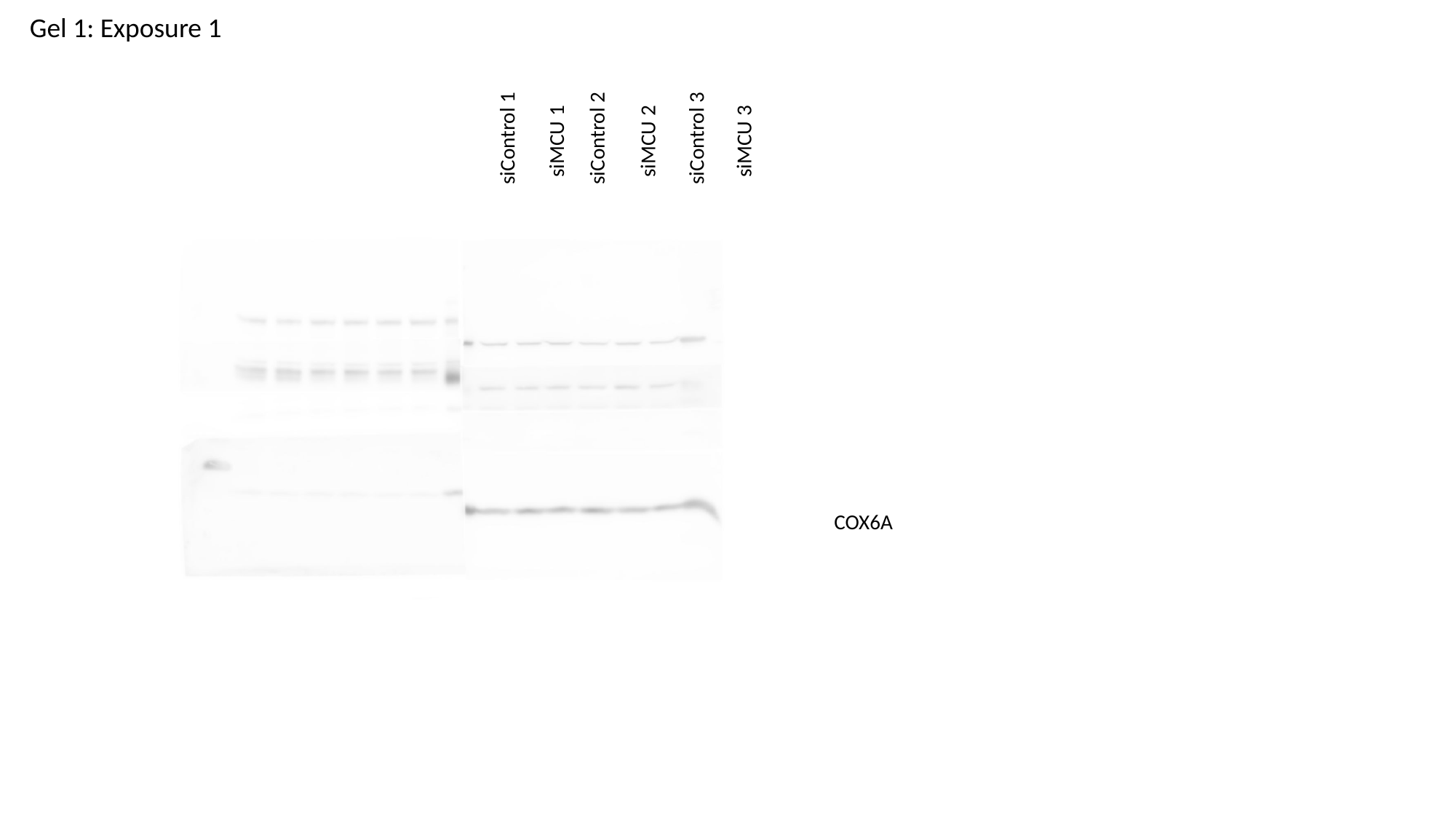

Gel 1: Exposure 1
siControl 1
siControl 2
siControl 3
siMCU 1
siMCU 2
siMCU 3
COX6A

## Slide 4
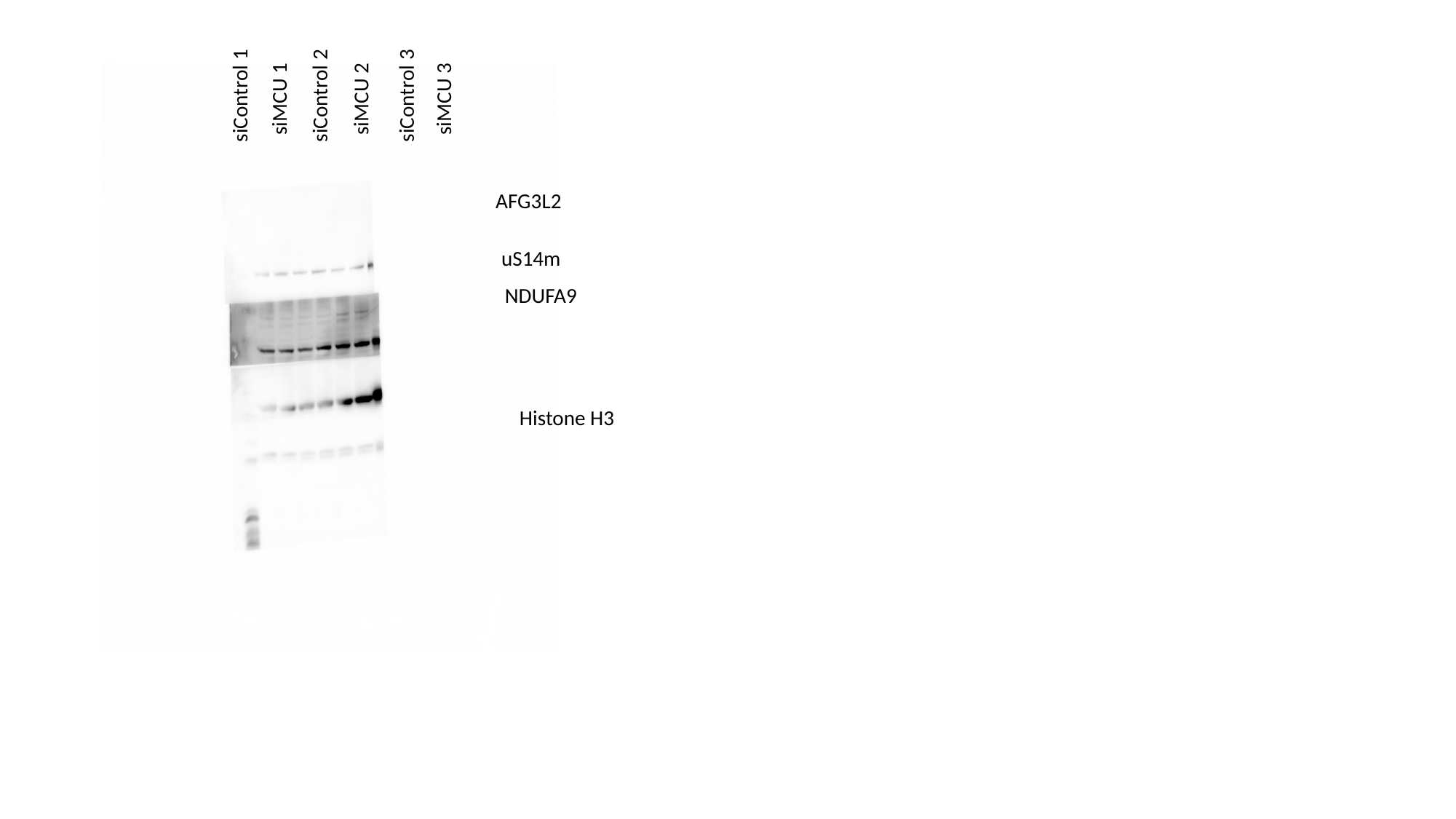

siControl 1
siControl 2
siControl 3
siMCU 1
siMCU 2
siMCU 3
AFG3L2
uS14m
NDUFA9
Histone H3
