# Supplementary material for: Mitochondrial calcium uniporter complex controls T-cell-mediated immune responses
Source: EMBO Rep. 2024 Dec 2;26(2):407–42. doi: 10.1038/s44319-024-00313-4 (PMC11772621; doi:10.1038/s44319-024-00313-4)
Supplement: Supplementary file 10 — Appendix and EV Figures Source Data [file 44319_2024_313_MOESM10_ESM.zip › Original Blot images_raw data-Appendix and EV figures.pptx]

## Slide 1
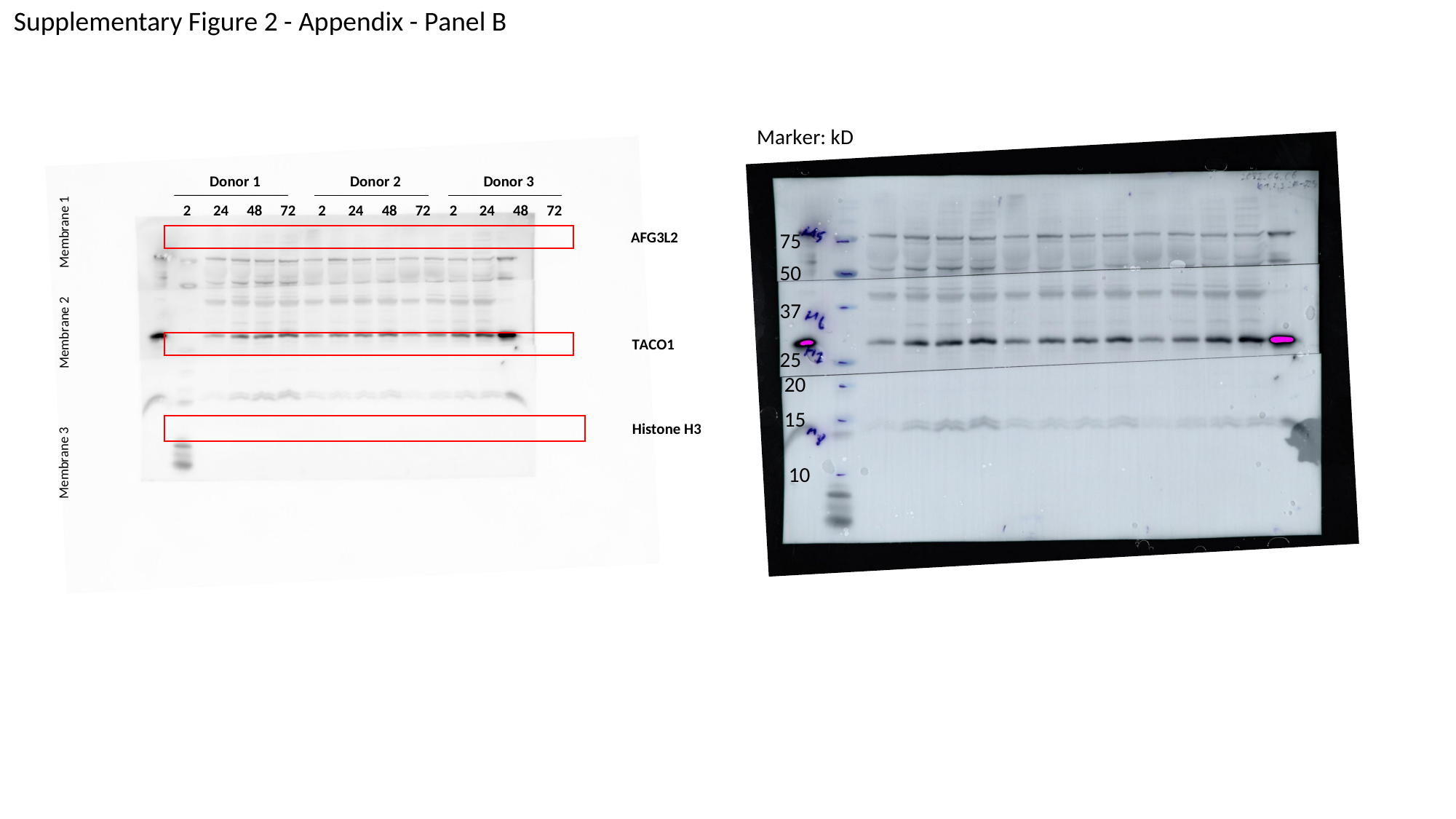

Supplementary Figure 2 - Appendix - Panel B
Marker: kD
Donor 3
Donor 1
Donor 2
72
24
48
72
72
2
24
48
24
48
2
2
Membrane 1
AFG3L2
75
50
37
Membrane 2
TACO1
25
20
15
Histone H3
Membrane 3
10

## Slide 2
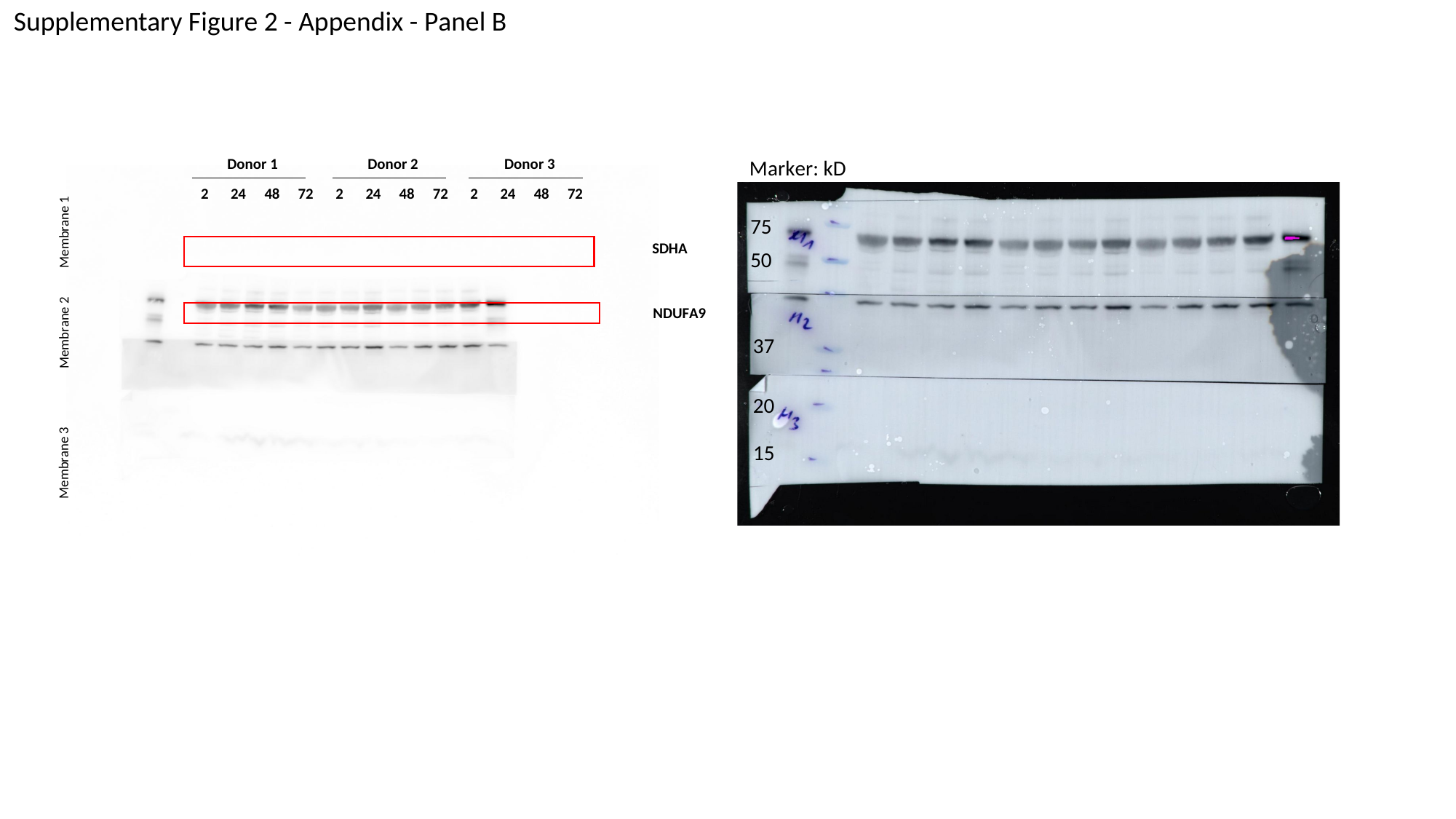

Supplementary Figure 2 - Appendix - Panel B
Donor 3
Marker: kD
Donor 1
Donor 2
72
24
48
72
72
2
24
48
24
48
2
2
75
Membrane 1
SDHA
50
NDUFA9
Membrane 2
37
20
15
Membrane 3

## Slide 3
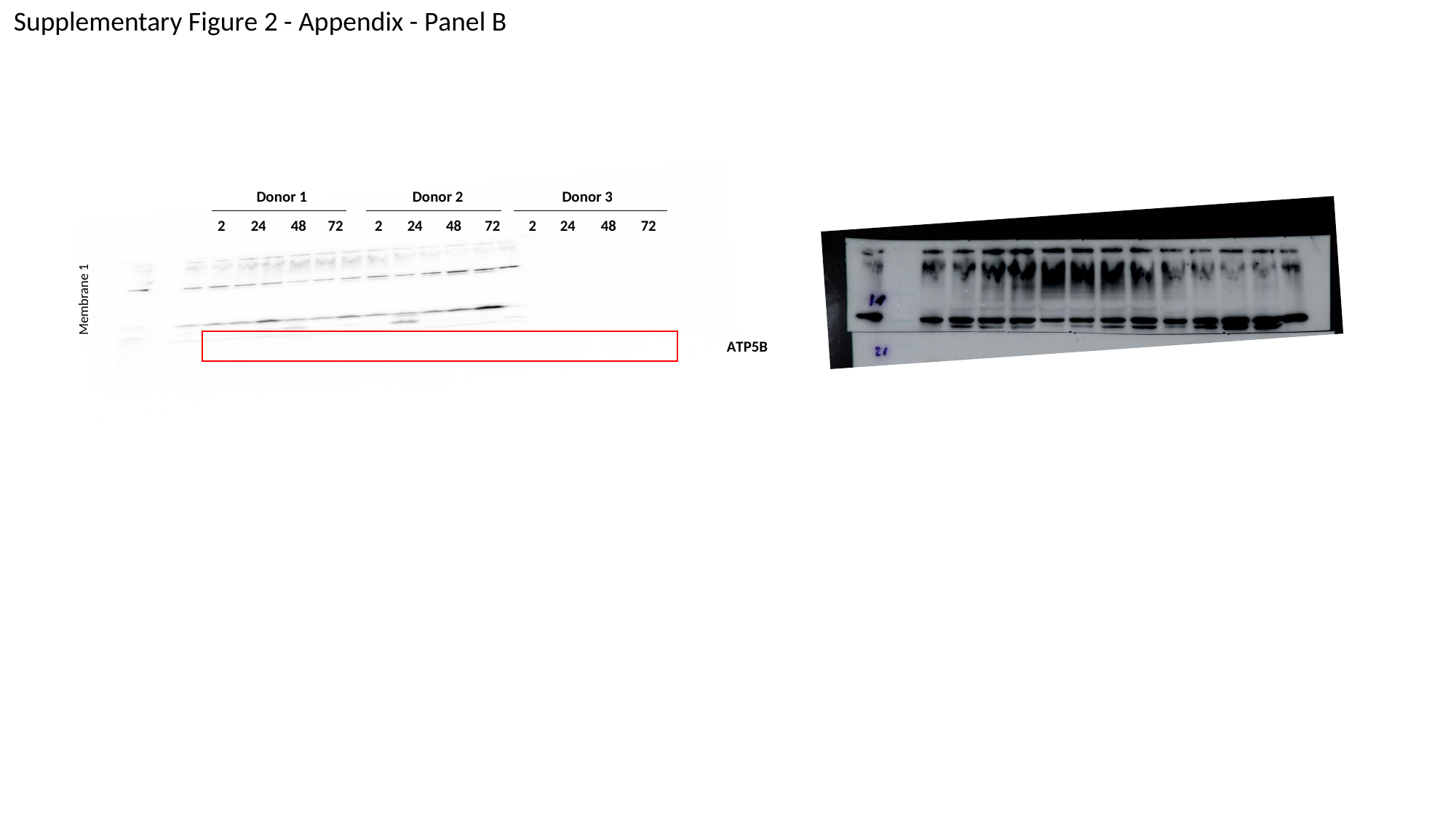

Supplementary Figure 2 - Appendix - Panel B
Donor 3
Donor 1
Donor 2
72
24
48
72
72
2
24
48
24
48
2
2
Membrane 1
ATP5B

## Slide 4
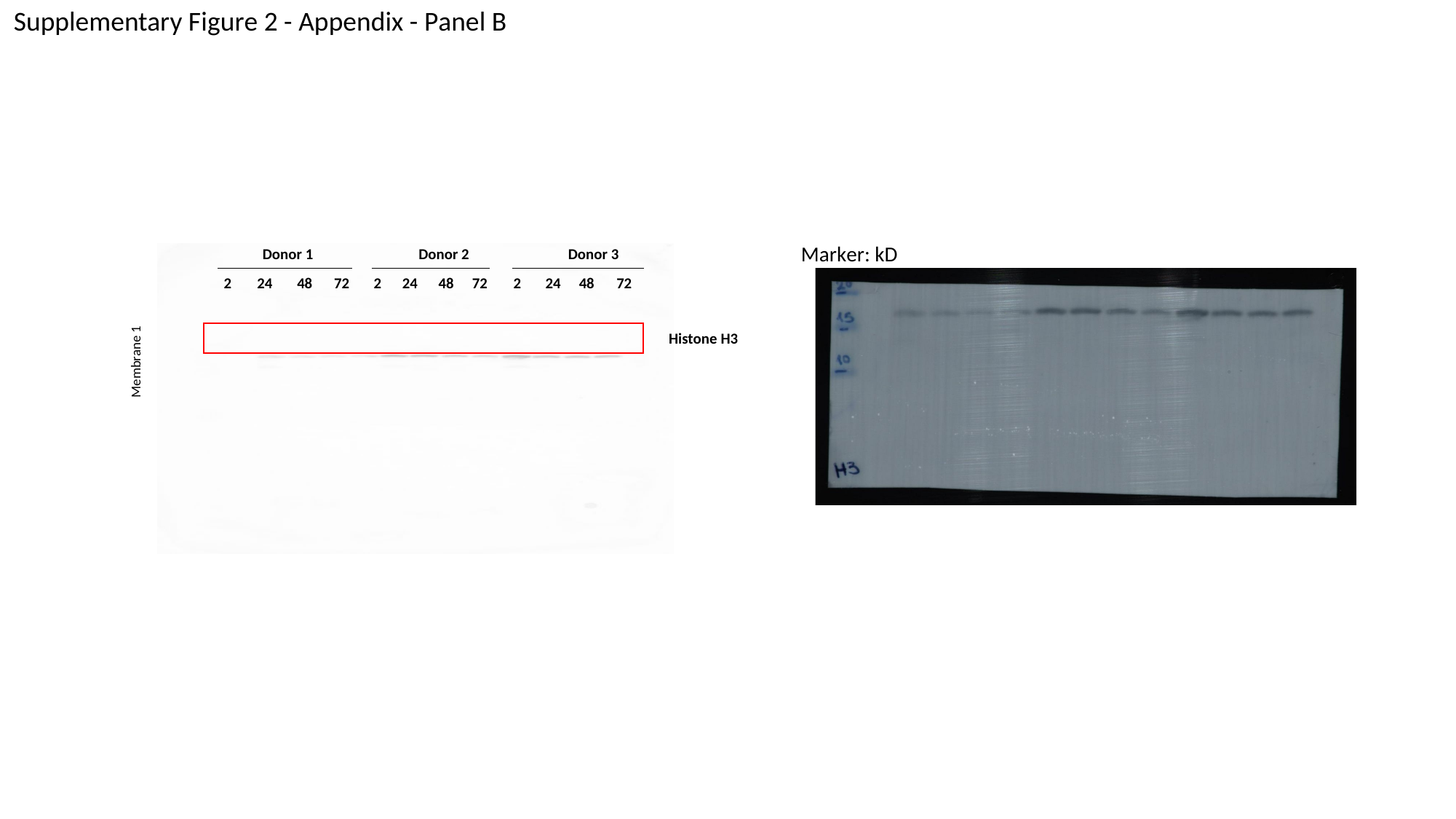

Supplementary Figure 2 - Appendix - Panel B
Marker: kD
Donor 3
Donor 1
Donor 2
72
24
48
72
72
2
24
48
24
48
2
2
Histone H3
Membrane 1

## Slide 5
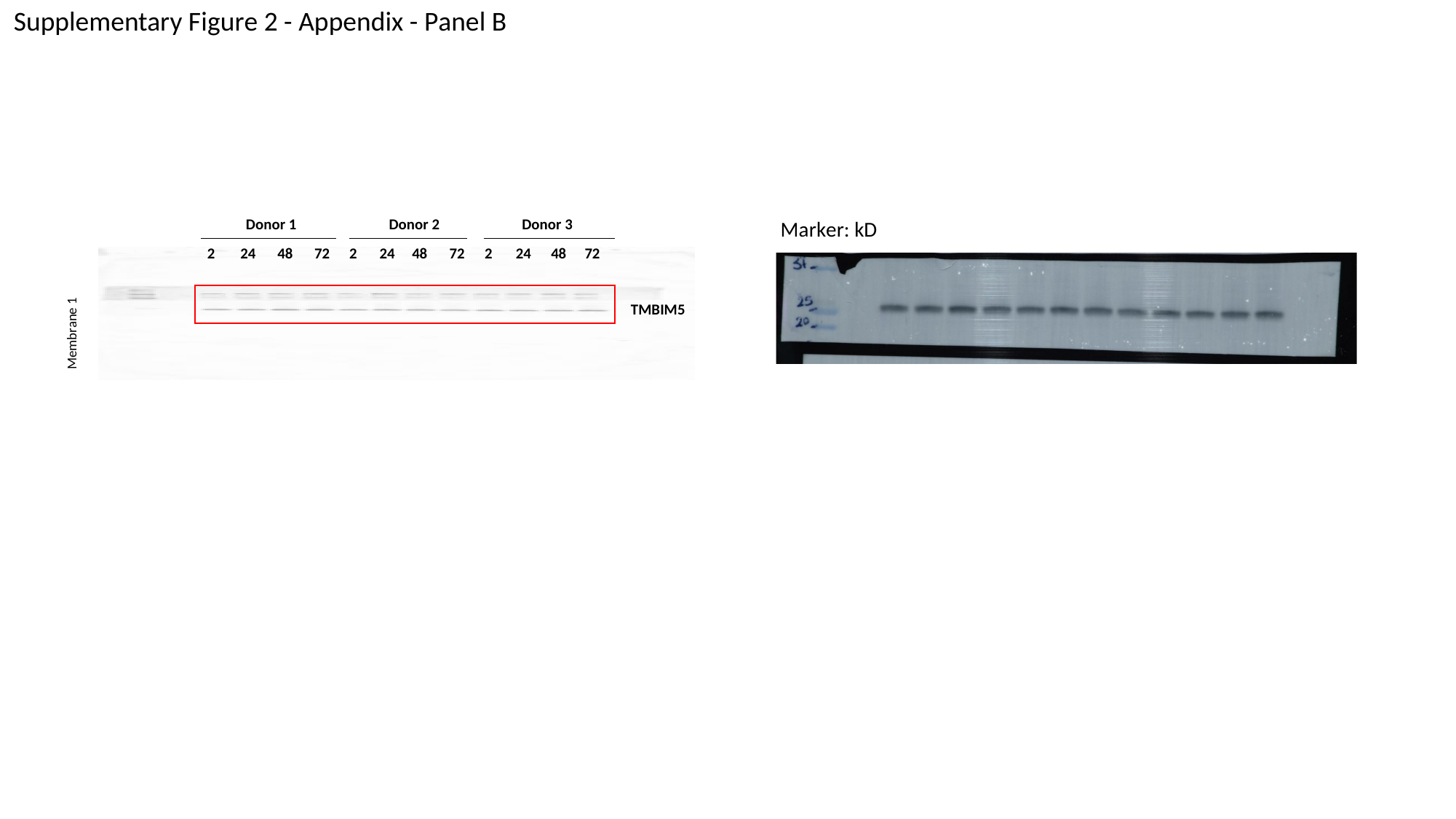

Supplementary Figure 2 - Appendix - Panel B
Donor 3
Donor 1
Donor 2
Marker: kD
72
24
48
72
72
2
24
48
24
48
2
2
TMBIM5
Membrane 1

## Slide 6
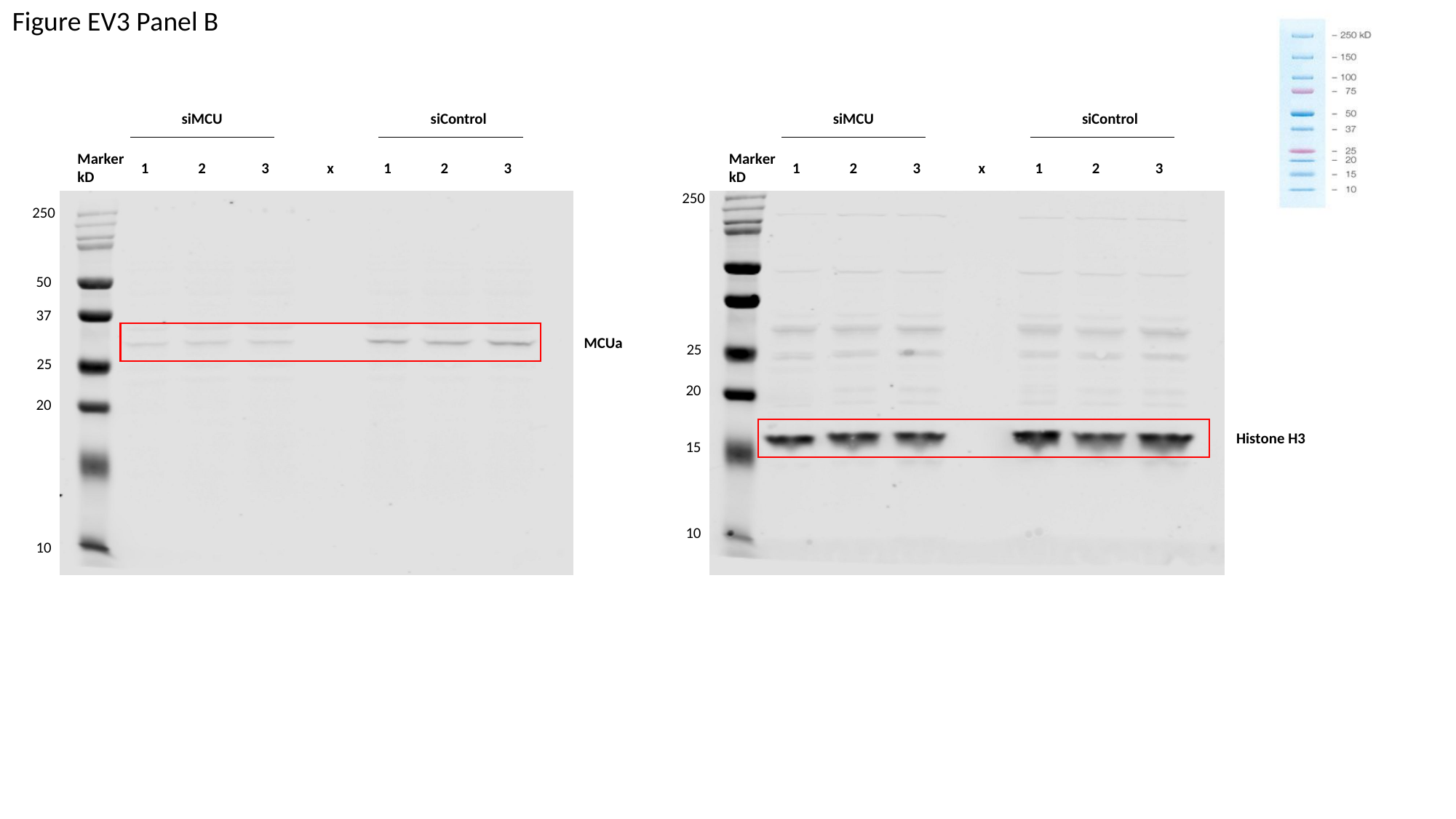

Figure EV3 Panel B
siControl
siControl
siMCU
siMCU
Marker
kD
Marker
kD
x
3
2
3
1
x
3
2
2
1
3
1
2
1
250
250
50
37
MCUa
25
25
20
20
Histone H3
15
10
10

## Slide 7
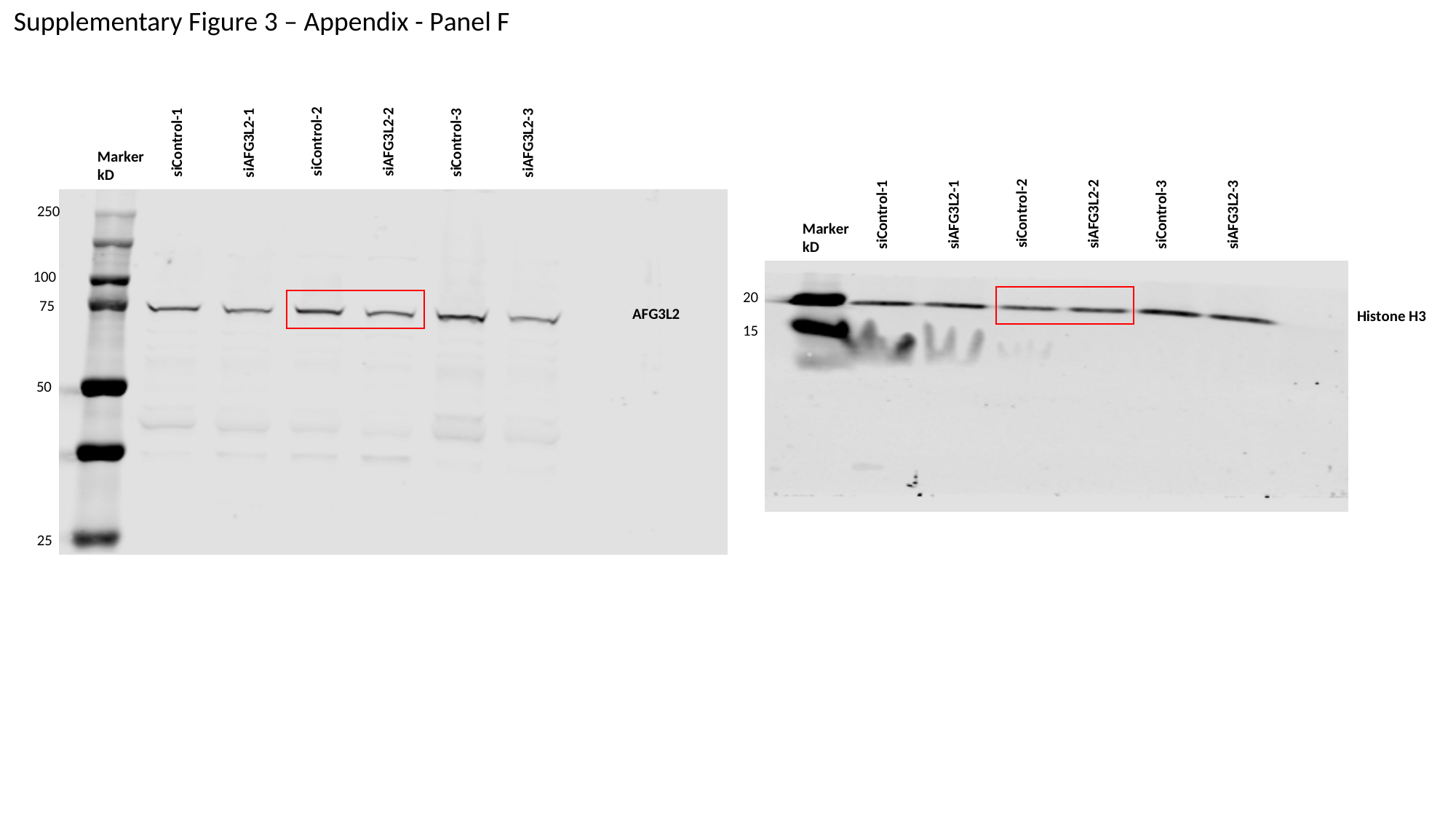

Supplementary Figure 3 – Appendix - Panel F
siControl-2
siAFG3L2-2
siControl-1
siControl-3
siAFG3L2-1
siAFG3L2-3
Marker
kD
250
siControl-2
siAFG3L2-2
siControl-1
siControl-3
siAFG3L2-1
siAFG3L2-3
Marker
kD
100
20
75
AFG3L2
Histone H3
15
50
25

## Slide 8
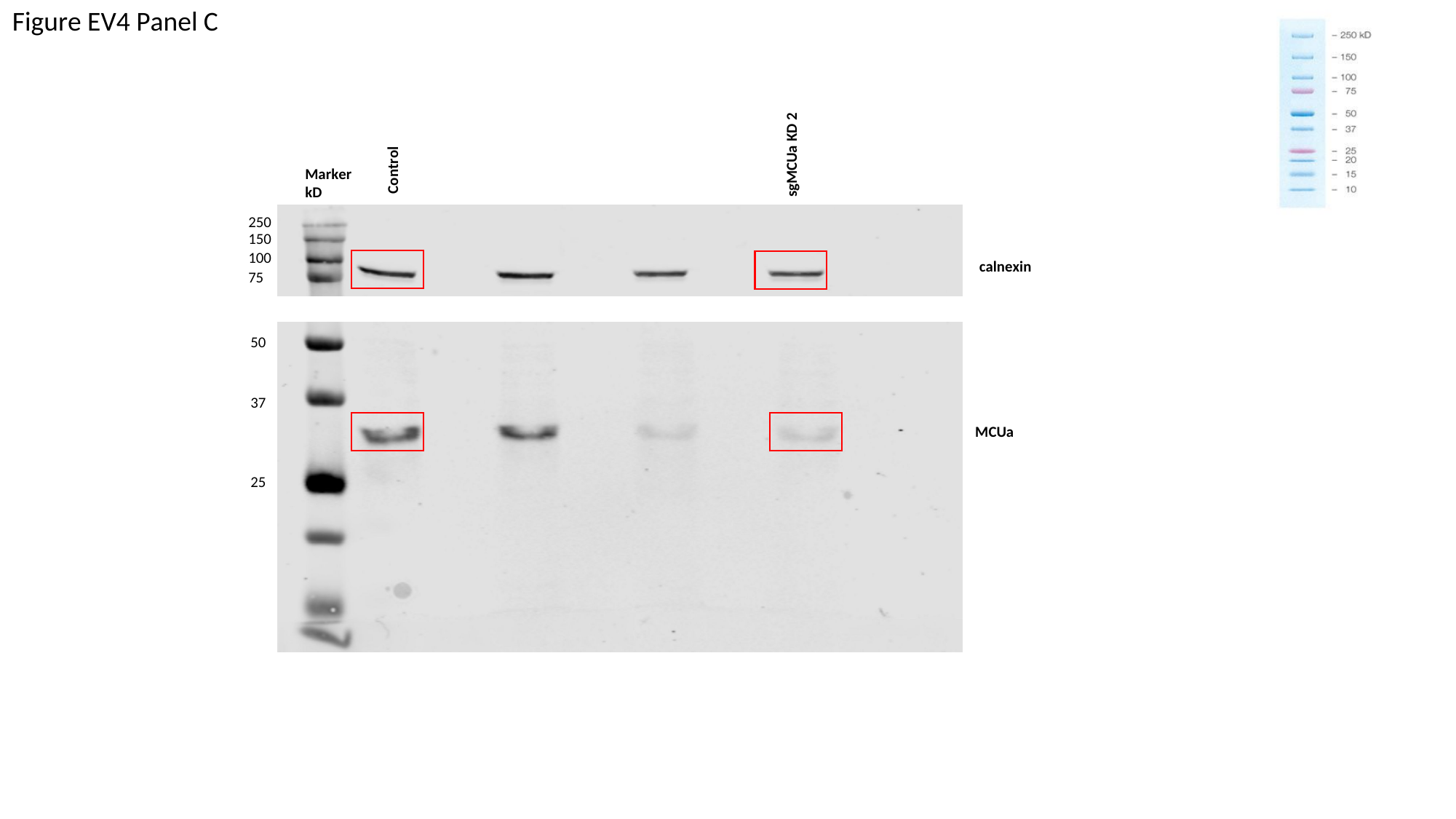

Figure EV4 Panel C
sgMCUa KD 2
Control
Marker
kD
250
150
100
calnexin
75
50
37
MCUa
25

## Slide 9
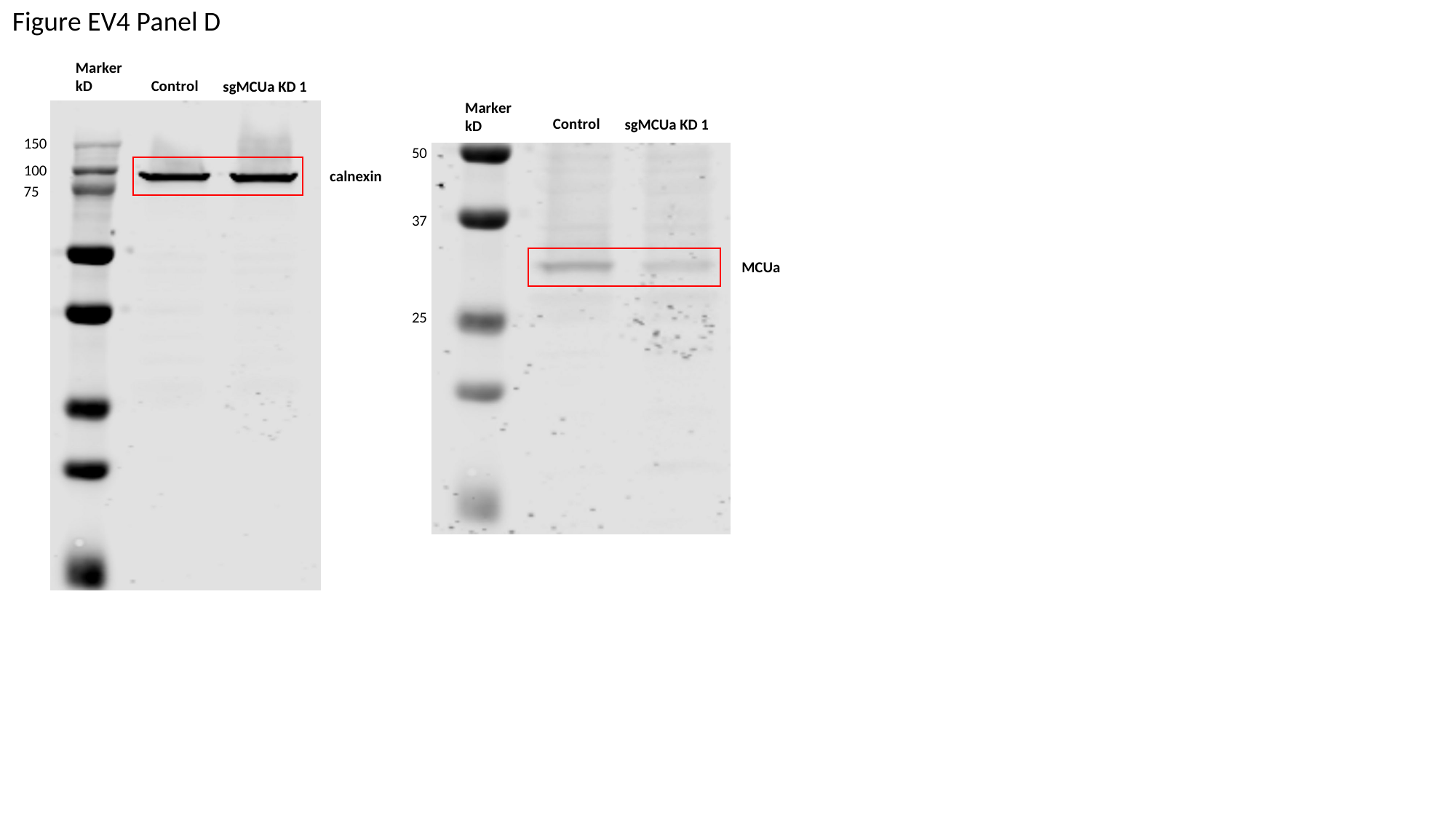

Figure EV4 Panel D
Marker
kD
Control
sgMCUa KD 1
Marker
kD
Control
sgMCUa KD 1
150
50
100
calnexin
75
37
MCUa
25
