# Supplementary material for: Mitochondrial calcium uniporter complex controls T-cell-mediated immune responses
Source: EMBO Rep. 2024 Dec 2;26(2):407–42. doi: 10.1038/s44319-024-00313-4 (PMC11772621; doi:10.1038/s44319-024-00313-4)
Supplement: Supplementary file 10 — Appendix and EV Figures Source Data [file 44319_2024_313_MOESM10_ESM.zip › Appendix Figure S3-F Immunoblot original images.pptx]

## Slide 1
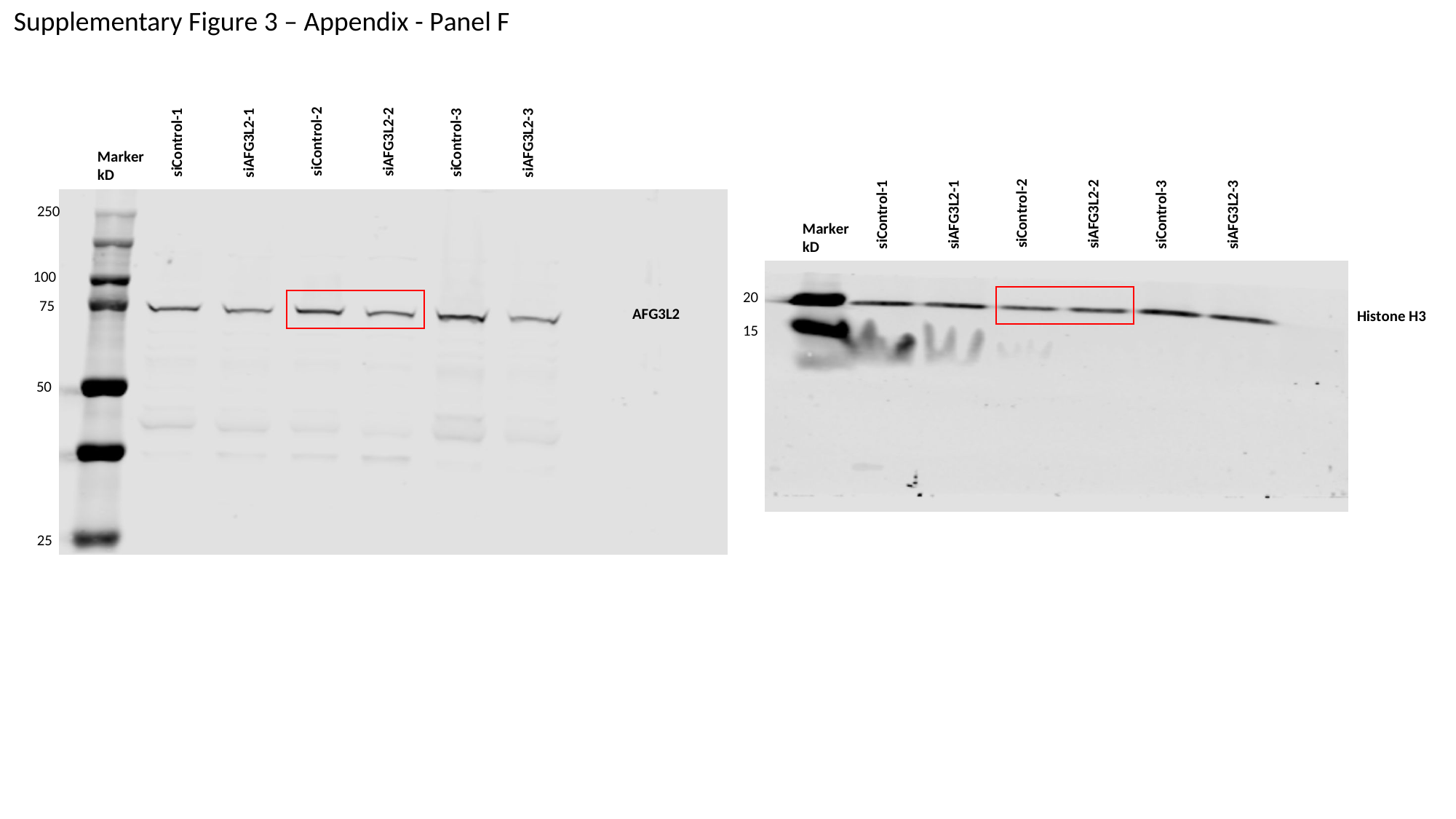

Supplementary Figure 3 – Appendix - Panel F
siControl-2
siAFG3L2-2
siControl-1
siControl-3
siAFG3L2-1
siAFG3L2-3
Marker
kD
250
siControl-2
siAFG3L2-2
siControl-1
siControl-3
siAFG3L2-1
siAFG3L2-3
Marker
kD
100
20
75
AFG3L2
Histone H3
15
50
25
