# Supplementary material for: Mitochondrial calcium uniporter complex controls T-cell-mediated immune responses
Source: EMBO Rep. 2024 Dec 2;26(2):407–42. doi: 10.1038/s44319-024-00313-4 (PMC11772621; doi:10.1038/s44319-024-00313-4)
Supplement: Supplementary file 11 — Expanded View Figures [file 44319_2024_313_MOESM11_ESM.pdf]

Expanded View Figures

Figure EV1. Cytosolic and mitochondrial Ca^{2+} levels are increased upon T-cell activation.

(**A**) Pure $CD4^+$ naive T-cell population isolated from blood of healthy human donors. Naive T-cells mainly exhibited positive CD4 (PE-A) staining, and not CD8. 99% and 96.9% of cells of from donor 1 (left) and donor 2 (right) were $CD4^+$, respectively. The histograms were generated from 10,000 events after gating. The gating strategy for flow cytometry is shown in Appendix Fig. S1. Legend: Grey=unstained control; Green=CD8 staining; Blue = CD4 staining. (**B**) Fura-2 measurements of $_cCa^{2+}$ in naive $CD4^+$ T-cells treated with anti-CD3/CD28-coated magnetic beads (1:5 bead:cell ratio) or antibody solution (1:1000 working solution). Quantification shown as mean ± SD of 1479 bead-activated and 1,656 antibody solution-activated T-cells/biological replicates from 5 healthy donors. ****$p < 0.0001$ ($p = 1.34872E{-}74$), assessed by two-tailed unpaired Student's t-test. (**C**) Naive T-cell proliferation following beads or antibody solution stimulation. Quantification shown as mean ± SEM of 8 healthy donors/ biological replicates. ***$p < 0.001$ ($p = 0.0006$); **$p < 0.01$ ($p = 0.0039$), assessed by two-tailed paired Student's t-test. RFU = relative fluorescence units. (**D**) Fura-2 measurements of $_cCa^{2+}$ in naive and effector T-cells. The SERCA-blocker thapsigargin (Tg; 1 µM) was used to deplete ER Ca^{2+} stores and activate SOCE. (**E**) Quantification of data given in (**D**). Data show mean ± SD of 768 naive T-cells and 577 effector T-cells/biological replicates from 3 healthy donors, normalised to their respective cell volume (in μm^3) as shown in (**G**). ****$p < 0.0001$ ($p = 2.73994E{-}12$), assessed by two-tailed unpaired Student's t-test. (**F**) Representative 3D images of a naive (left) and an effector (right) T-cell. Using pixel classification in Imaris v10.1.0, the cell surface (green) and the mitochondria (red) were created (refer to methods for more details). Quantification of cellular (**G**) and mitochondrial (**H**) volume in naive and effector T-cells showing a mean ± SEM of 66 naive and 52 effector T-cells/biological replicates from 3 healthy donors. Cell volume was quantified by using Machine Learning-based segmentation. ****$p < 0.0001$ ($p = 2.40289E{-}45$) and ($p = 2.3739E{-}37$), assessed by two-tailed unpaired Student's t-test. (**I**) Measurements of $_mCa^{2+}$ levels with the 4mtTNXL biosensor in human naive and effector T-cells. Quantification plots show an average of 118 naive and 331 effector cells/biological replicates from 2 different donors. Cell stimulation was achieved by addition of 1 µM Tg at the indicated time point. The quantification/violin plots show mean ± SD of basal $_mCa^{2+}$ levels and maximal $_mCa^{2+}$ uptake. ****$p < 0.0001$ (Basal $p = 1.6523E{-}33$; Uptake $p = 8.3596E{-}16$), assessed by two-tailed unpaired Student's t-test. (**J**) Measurements of $_mCa^{2+}$ levels with the 4mtTNXL biosensor in human naive and effector T-cells. Traces show an average of 4 naive and 16 effector cells/biological replicates from 1 healthy donor. Cells were stimulated with anti-human CD3/CD28-coated beads at the indicated time point. The quantification/violin plots show mean ± SD for both the basal $_mCa^{2+}$ levels and $_mCa^{2+}$ uptake. ***$p < 0.001$ ($p = 0.0004$). (**K**) Calcium Retention Capacity (CRC) assay measuring $_mCa^{2+}$ uptake in digitonin-permeabilized naive and effector T-cells using Calcium-Green-5N. Traces show mean ± SEM relative fluorescence units (RFU) of 10 naive and 10 effector T-cells/biological replicates from 3 healthy donors (assays always performed at least in duplicate). $_mCa^{2+}$ uptake was induced by subsequent additions of a bolus of Ca^{2+} at the indicated time points (black arrows). The quantification plots show mean ± SEM for basal $_mCa^{2+}$ uptake at the time points indicated by a red star. $_mCa^{2+}$ uptake was quantified as the delta decrease in measured RFU upon Ca^{2+} addition. *$p \leq 0.05$ ($p = 0.0481$-left; $p = 0.0443$-right panel), assessed by two-tailed unpaired Student's t-test.

 © The Author(s)

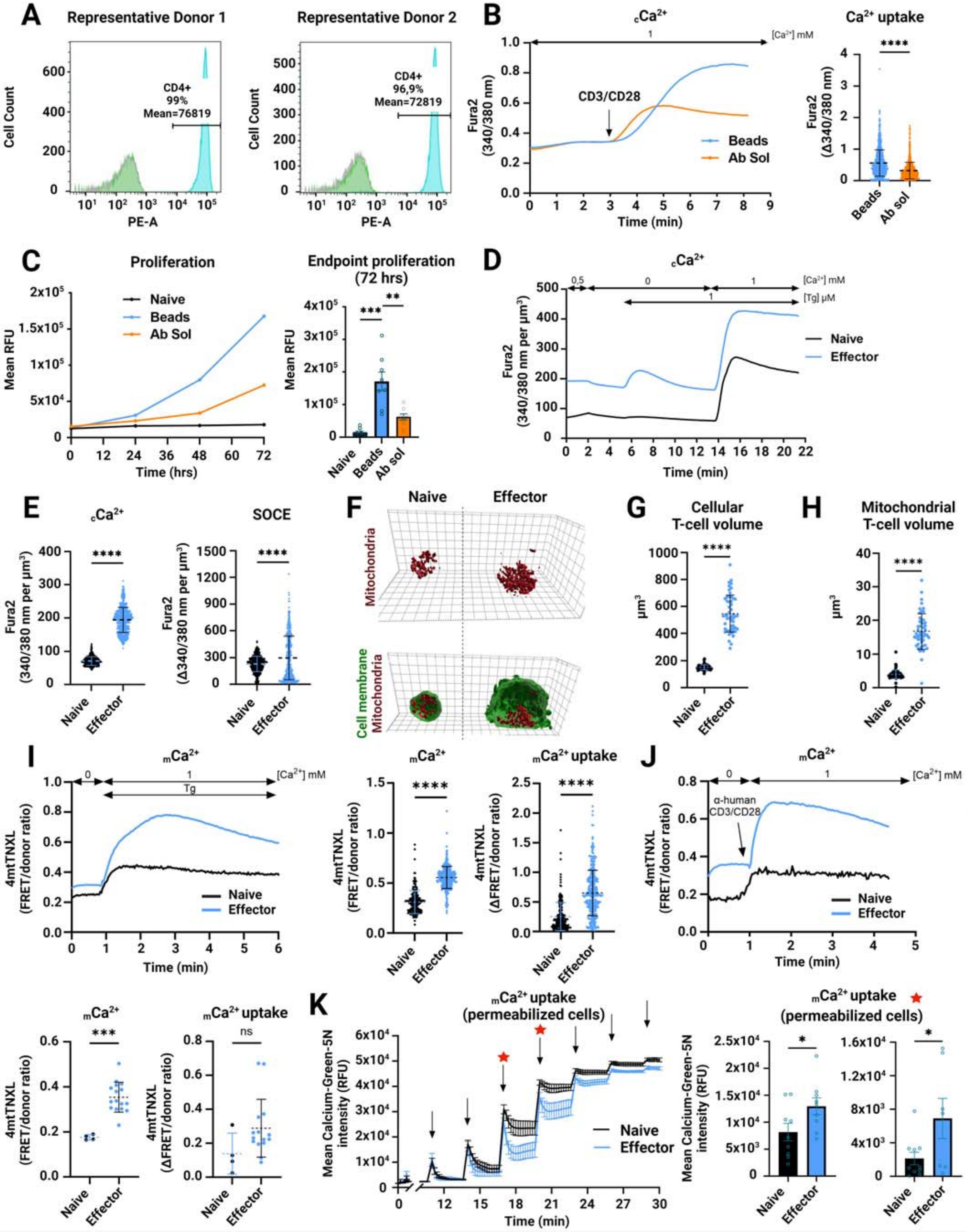
A
Representative Donor 1
Cell Count
CD4+
99%
Mean=76819
PE-A
Representative Donor 2
CD4+
96,9%
Mean=72819
B
cCa2+
[Ca2+] mM
Fura2 (340/380 nm)
CD3/CD28
Beads
Ab Sol
Time (min)
Ca2+ uptake
****
Fura2 (Δ340/380 nm)
Beads
Ab sol
C
Proliferation
Mean RFU
Naive
Beads
Ab Sol
Time (hrs)
Endpoint proliferation (72 hrs)
***
**
Naive
Beads
Ab sol
D
cCa2+
[Ca2+] mM
[Tg] μM
Fura2 (340/380 nm per μm3)
Naive
Effector
Time (min)
E
cCa2+
****
Fura2 (340/380 nm per μm3)
SOCE
Fura2 (Δ340/380 nm per μm3)
Naive
Effector
F
Naive
Effector
Mitochondria
Cell membrane
Mitochondria
G
Cellular T-cell volume
****
μm3
H
Mitochondrial T-cell volume
****
μm3
I
mCa2+
[Ca2+] mM
Tg
4mtTNXL (FRET/donor ratio)
Naive
Effector
Time (min)
mCa2+
****
mCa2+ uptake
****
4mtTNXL (ΔFRET/donor ratio)
J
mCa2+
[Ca2+] mM
α-human CD3/CD28
4mtTNXL (FRET/donor ratio)
Naive
Effector
Time (min)
mCa2+
***
4mtTNXL (FRET/donor ratio)
mCa2+ uptake
ns
4mtTNXL (ΔFRET/donor ratio)
K
mCa2+ uptake (permeabilized cells)
Mean Calcium-Green-5N intensity (RFU)
Naive
Effector
Time (min)
mCa2+ uptake
(permeabilized cells)
*
*
Mean Calcium-Green-5N intensity (RFU)

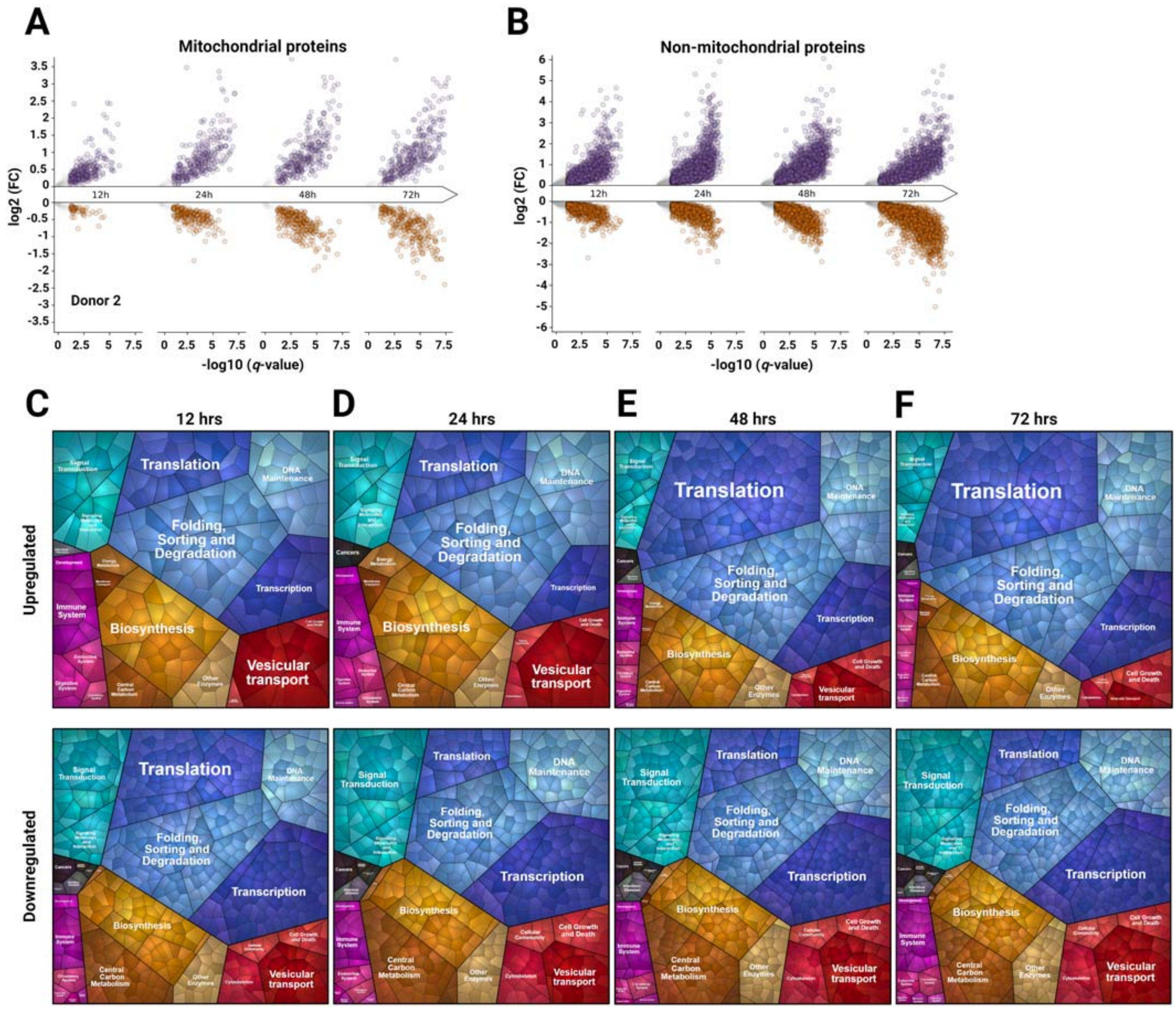

Figure EV2. Activation of naive human T-cells causes time-dependent proteome alterations.

Proteomics and bioinformatic analyses of bead-activated T-cells for 12, 24, 48, and 72 h. The volcano plots (**A**, **B**) show significantly up- (purple) and downregulated (orange) proteins as compared to naive T-cells. (**C–F**) Proteomap-based evaluation of signalling pathways and cellular functions regulated by significantly up- (upper panels) and downregulated (lower panels) proteins following bead-induced T-cell activation (from 12 to 72 h). Proteomaps are based on differentially-expressed proteins (DEPs) depicted in Fig. 3F.

 © The Author(s)

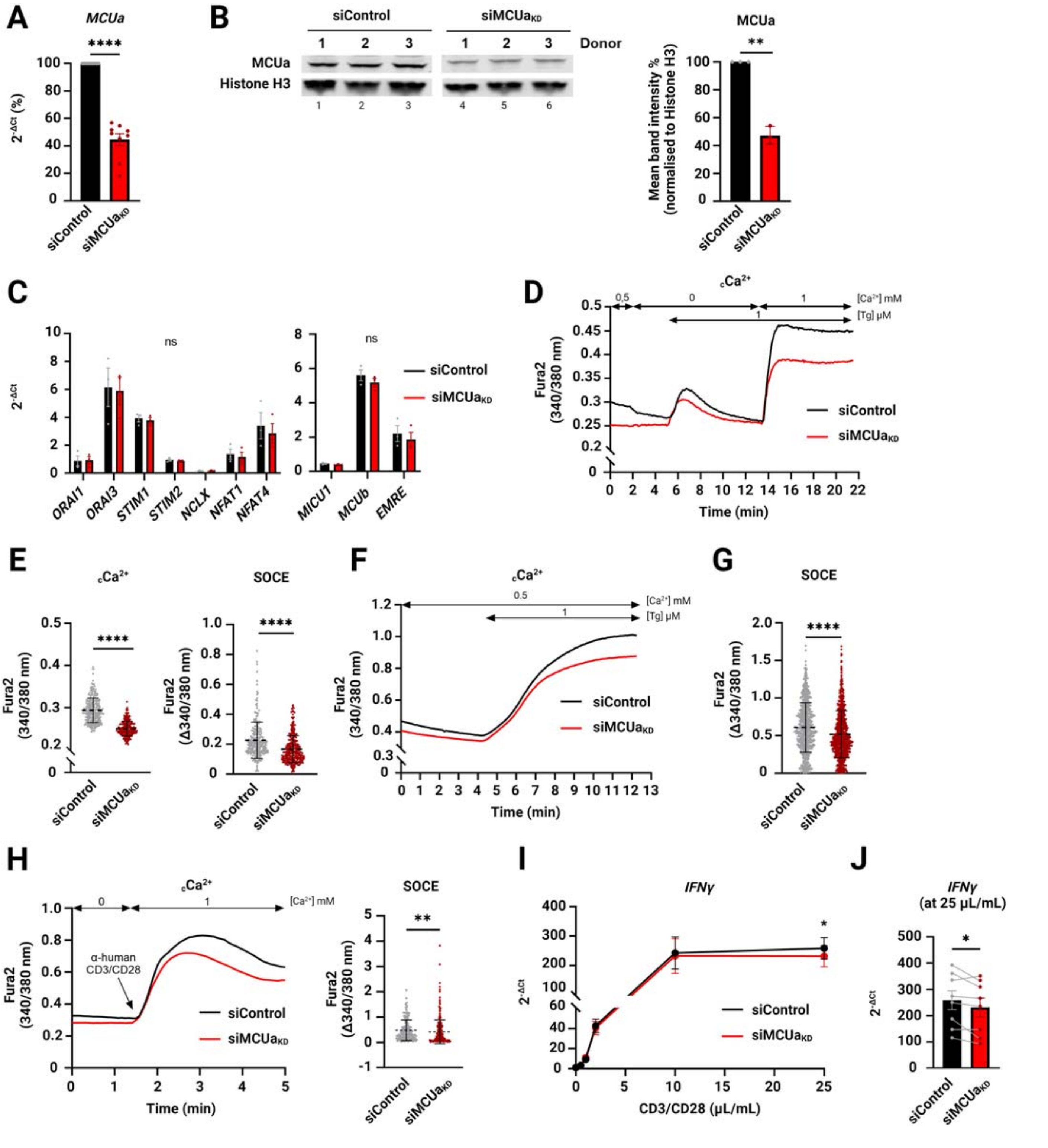
A
MCUa
****
siControl
siMCUaKD
B
siControl
siMCUaKD
Donor
MCUa
Histone H3
Mean band intensity % (normalised to Histone H3)
**
C
ns
ORAI1
ORAI3
STIM1
STIM2
NCLX
NFAT1
NFAT4
MICU1
MCUb
EMRE
D
cCa2+
[Ca2+] mM
[Tg] µM
Fura2 (340/380 nm)
Time (min)
E
SOCE
Fura2 (Δ340/380 nm)
F
G
H
α-human CD3/CD28
I
IFNγ
CD3/CD28 (µL/mL)
J
IFNγ (at 25 µL/mL)

Figure EV3. $MCUa_{KD}$ affects human effector T-cell function.

(**A**) RT-qPCR-based evaluation of transient MCUa knockdown efficiency in human $CD4^{+}$ effector T-cells. The quantified graph shows mean ± SEM of 9 healthy donors/ biological replicates, as compared to control transfection. ****$p < 0.0001$ ($p = 3.89251E-13$), assessed by two-tailed paired Student's t-test. (**B**) Immunoblot-based evaluation of transient MCUa knockdown efficiency in human $CD4^{+}$ effector T-cells. Quantification shows mean ± SEM from 3 healthy donors/biological replicates as compared to control transfection, normalised to Histone H3. **$p < 0.01$ ($p = 0.0033$), assessed by two-tailed paired Student's t-test. (**C**) RT-qPCR-based evaluation of Ca^{2+} signalling-related genes and MCU complex components in effector T-cells with transient knockdown of *MCUa*. Quantifications show mean ± SEM from 3 healthy donors/ biological replicates for all assessed genes. ns - not significant, assessed by two-tailed paired Student's t-test. (**D**, **E**) Measurements of $_{c}Ca^{2+}$ levels using Fura2 in control and $siMCUa_{KD}$ effector T-cells. Quantification shows mean ± SD of 648 control and 739 $siMCUa_{KD}$ T-cells/biological replicates from 3 healthy donors. Tg = 1 µM. ****$p < 0.0001$ (Basal $p = 8.86452E-78$; SOCE $p = 3.26694E-11$), assessed by two-tailed unpaired Student's t-test. (**F**, **G**) Measurements of $_{c}Ca^{2+}$ levels using Fura2 in $siMCUa_{KD}$ effector T-cells. Quantification shows mean ± SD of 848 control and 930 $siMCUa_{KD}$ T-cells/biological replicates from 2 healthy donors. Tg = 1 µM. ****$p < 0.0001$ ($p = 5.75699E-46$), assessed by two-tailed unpaired Student's t-test. (**H**) Measurements of $_{c}Ca^{2+}$ levels using Fura2 in control and $siMCUa_{KD}$ effector T-cells stimulated with anti-human CD3/CD28-coated beads. Quantification shows mean ± SD of 169 control and 365 $siMCUa_{KD}$ T-cells/biological replicates from 2 healthy donors. **$p < 0.01$ ($p = 0.0033$), assessed by Student's t-test. (**I**, **J**) *IFNγ* mRNA levels in re-stimulated $siMCUa_{KD}$ T-cells. The quantification (at 25 µL/mL Ab solution) shows mean ± SEM of 8 healthy donors/biological replicates. *$p \leq 0.05$ ($p = 0.0457$), assessed by two-tailed paired Wilcoxon test. Source data are available online for this figure.

 © The Author(s)

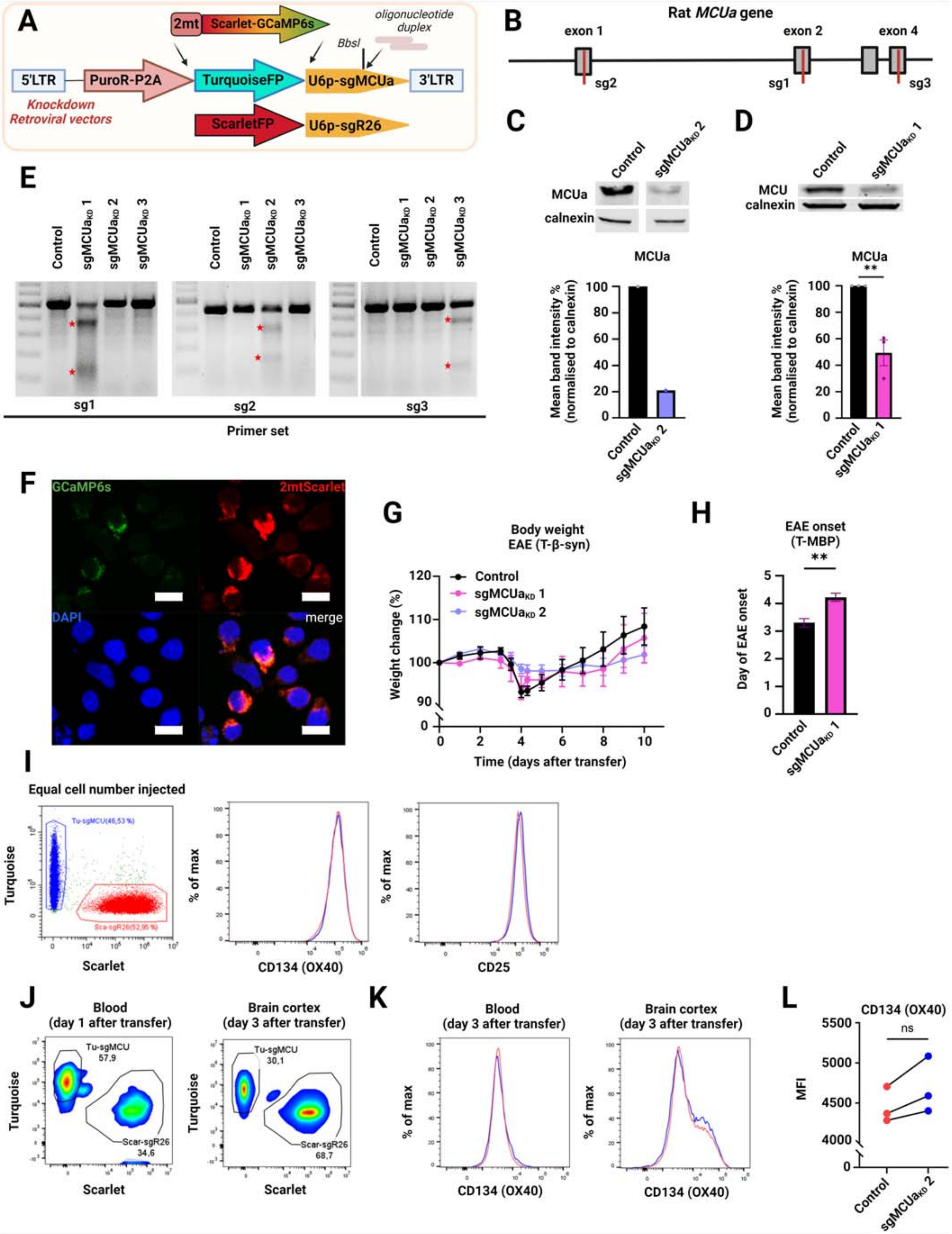
A
2mt Scarlet-GCaMP6s
oligonucleotide duplex
BbsI
5'LTR
PuroR-P2A
TurquoiseFP
U6p-sgMCUa
3'LTR
Knockdown Retroviral vectors
ScarletFP
U6p-sgR26
B
Rat MCUa gene
exon 1
exon 2
exon 4
sg2
sg1
sg3
C
Control
sgMCUaKD 2
MCUa
calnexin
MCUa
Mean band intensity % (normalised to calnexin)
D
Control
sgMCUaKD 1
MCU
calnexin
MCUa
**
E
Control
sgMCUaKD 1
sgMCUaKD 2
sgMCUaKD 3
sg1
sg2
sg3
Primer set
F
GCaMP6s
2mtScarlet
DAPI
merge
G
Body weight
EAE (T-β-syn)
Control
sgMCUaKD 1
sgMCUaKD 2
Weight change (%)
Time (days after transfer)
H
EAE onset
(T-MBP)
**
Day of EAE onset
Control
sgMCUaKD 1
I
Equal cell number injected
Tu-sgMCU(46,53 %)
Sca-sgR26(52,95 %)
Turquoise
Scarlet
% of max
CD134 (OX40)
CD25
J
Blood
(day 1 after transfer)
Tu-sgMCU
57,9
Scar-sgR26
34,6
Brain cortex
(day 3 after transfer)
Tu-sgMCU
30,1
Scar-sgR26
68,7
Turquoise
Scarlet
K
Blood
(day 3 after transfer)
Brain cortex
(day 3 after transfer)
% of max
CD134 (OX40)
L
CD134 (OX40)
ns
MFI
Control
sgMCUaKD 2

Figure EV4. Establishment of $MCUa_{KD}$ models in rat effector T-cells.

(**A**) Schematic representation of retroviral constructs used to establish control and $sgMCUa_{KD}$ rat effector T-cell lines. LTR – long terminal repeats; PuroR – puromycin resistance gene; U6p – U6 promoter; 2mt – tandem mitochondrial localisation signals. (**B**) Schematic representation of the rat *MCUa* gene and the position of protospacers targeted by single guide RNAs (sg; the numbering of exons starts from the first coding exon) (Images (**A**) and (**B**) created with BioRender). (**C**, **D**) Immunoblot-based evaluation of knockdown efficiency in $sgMCUa_{KD}$ 2 (**C**) and $sgMCUa_{KD}$ 1 (**D**) cell lines. Protein expression data are normalised to the loading control calnexin. Quantification shows mean ± SEM of 3 experiments/biological replicates. $^{**}p < 0.01$ ($p = 0.0065$), assessed by two-tailed unpaired Student's t-test, in (**D**). (**E**) PCR-based T7 endonuclease cleavage confirmed the intended editing of the respective sequence in the different $sgMCUa_{KD}$ cell lines. T7 cleavage fragments, marked by a star, revealed sequence alterations (indels) introduced by Cas9 at a genomic level. (**F**) Confocal images of DAPI-stained $sgMCUa_{KD}$ 1 T-cells transduced by Puro2A-2mtScarlet-GCaMP6s retrovirus show proper mitochondrial localisation of the biosensor 2mtScarlet-GCaMP6s. Scale bar: 10 µm. (**G**) Body weight change curves in animals during transfer EAE experiments (related to Fig. 6H). Data show mean ± SEM, as percentage of initial weight on day 0 ($n = 4$ animals for the control group and $n = 3$ animals for the $sgMCUa_{KD}$ 1 and 2 groups). (**H**) Inactivation of MCUa results in a delayed EAE onset in rats injected with MBP-specific effector T-cells. Data are represented as mean ± SEM of 3 animals/biological replicates per condition. $^{**}p < 0.01$ ($p = 0.0018$), assessed by Mann–Whitney U test. (**I**) (related to Fig. 7). An equal number of different fluorescently-labelled control (Scarlet fluorescent protein) and $sgMCUa_{KD}$ (Turquoise fluorescent protein) T-cells were co-transferred in Lewis rats. Both cell lines that were injected showed a similar activation state assessed by CD134 (OX-40) and CD25 expression using flow cytometry. (**J**) Ex vivo-isolated T-cells were analysed at different time points after co-transfer using flow cytometry. At day 1 after co-transfer, $sgMCUa_{KD}$ T-cells (57.9%) outnumbered the control cells (34.6%) in the blood. At day 3 after co-transfer, the control T-cells (68.7%) outnumbered the $sgMCUa_{KD}$ cells (30.1%) in the brain. (**K**) $sgMCUa_{KD}$ T-cells that have succeeded to enter the CNS show comparable activation as those cells in the blood, determined by CD134 (OX-40) expression. (**L**) OX-40 expression of control and $sgMCUa_{KD}$ T-cells in the brain at day 3 after co-transfer. Quantification shows mean fluorescence intensity (MFI) of CD134 from 3 rats/biological replicates. ns - not significant, assessed by unpaired Student's t-test. Source data are available online for this figure.

 © The Author(s)

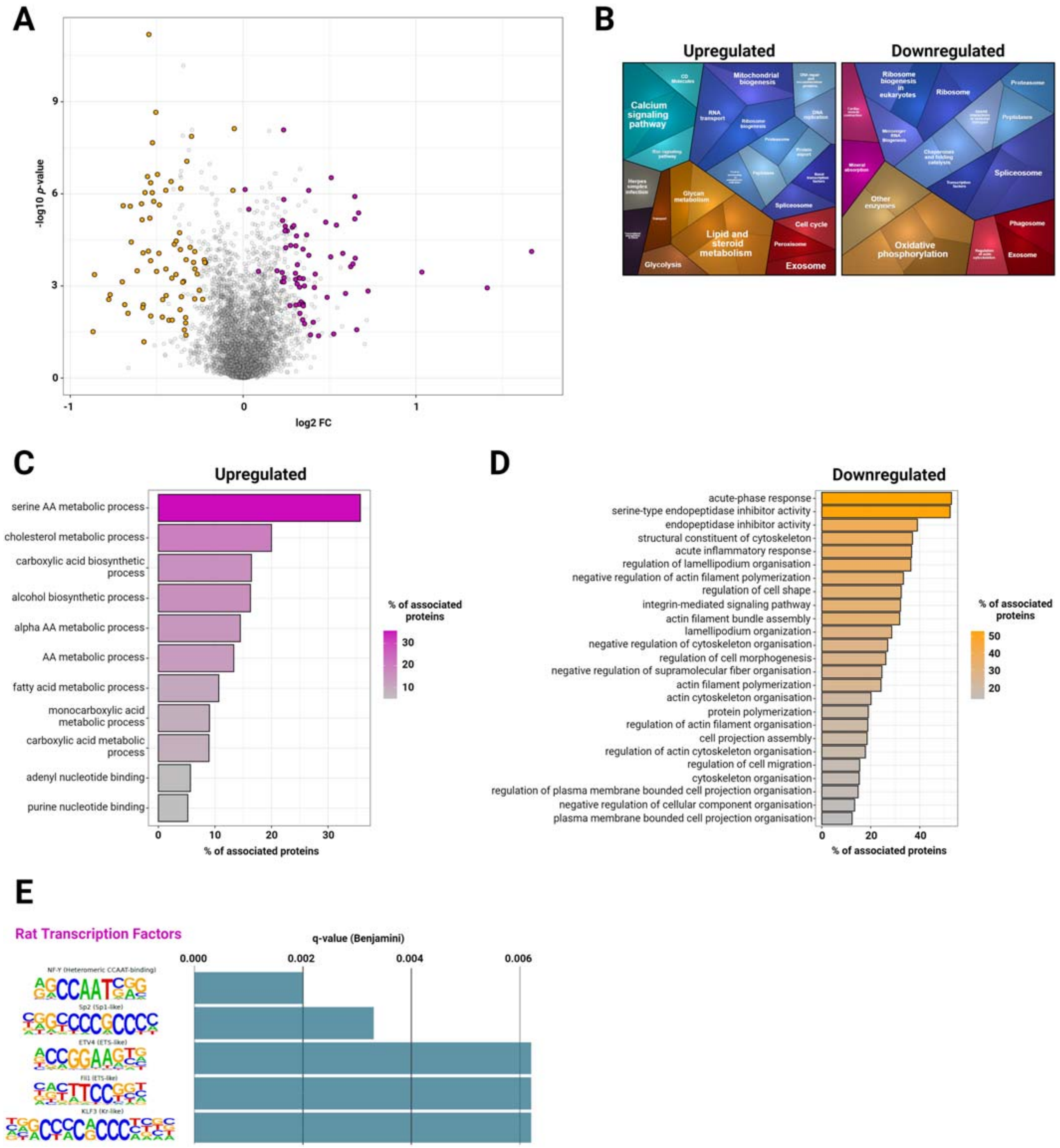

Figure EV5. $MCUa_{KD}$ in rat effector T-cells alters their proteome.

Proteomics data generated from the two $sgMCUa_{KD}$ cell lines and the control rat T-cell line. (**A**) The volcano plot shows multiple significant up- (magenta) and downregulated (orange) proteins in the two $sgMCUa_{KD}$ cell lines, compared to control, analysed by ANOVA (5% FDR). (**B**) Proteomaps showing the quantitative composition of proteomes with a high probability to be significantly up- (left panel) and downregulated (right panel) upon MCUa knockdown in rat effector T-cells. Proteomaps are based on DEPs shown in (**A**). (**C**, **D**) Significant up- (**C**) and downregulated (**D**) cellular functions and signalling pathways in $sgMCUa_{KD}$ cells ordered by percentage of associated proteins, based on DEPs shown in (**A**). (**E**) HOMER analysis of the proteomic data showing the top five most enriched motifs (TFs). Analysis is based on *q*-values (Benjamini-Hochberg-C).

© The Author(s)
