# Supplementary material for: Mitochondrial calcium uniporter complex controls T-cell-mediated immune responses
Source: EMBO Rep. 2024 Dec 2;26(2):407–42. doi: 10.1038/s44319-024-00313-4 (PMC11772621; doi:10.1038/s44319-024-00313-4)
Supplement: Supplementary file 1 — Peer Review File [file 44319_2024_313_MOESM1_ESM.pdf]

EMBO reports

Mitochondrial calcium uniporter complex controls T-cell-mediated immune responses

Magdalena Shumanska, Dimitry Lodygin, Christine Gibhardt, Christian Ickes, Ioana Stejerean-Todoran, Lena Krause, Kira Pahl, Lianne Jacobs, Andrea Paluschkiwitz, Shuya Liu, Angela Boshnakovska, Tobias Legler, Martin Haubrock, Miso Mitkovski, Niels Voigt, Gereon Poschmann, Peter Rehling, Sven Dennerlein, Jan Riemer, Alexander Flügel, and Ivan Bogeski

Corresponding author(s): Ivan Bogeski (ivan.bogeski@med.uni-goettingen.de)

Review Timeline:

Transfer Date:	29th May 24
Editorial Decision:	4th Jun 24
Revision Received:	11th Sep 24
Editorial Decision:	21st Oct 24
Revision Received:	24th Oct 24
Accepted:	25th Oct 24

Editor: Achim Breiling

Transaction Report: A revised version of this manuscript was transferred to EMBO reports following peer review at EMBO Molecular Medicine.

(Note: With the exception of the correction of typographical or spelling errors that could be a source of ambiguity, letters and reports are not edited. Depending on transfer agreements, referee reports obtained elsewhere may or may not be included in this compilation. Referee reports are anonymous unless the Referee chooses to sign their reports.)

Dear Prof. Bogeski,

Thank you for transferring your revised manuscript to EMBO reports. I now went through the manuscript, the referee reports from EMBO Molecular Medicine (attached again below) and your further revision plan (rebuttal/appeal letter). Referees #1 and #3 have remaining concerns and suggestions to improve the manuscript, or to strengthen the data and the conclusions drawn.

I would thus like to invite you to revise your manuscript with the understanding that all remaining concerns of the referees must be addressed in the revised manuscript or in a detailed point-by-point response, as indicated in your revision plan.

Moreover, I have these editorial requests:

- There is an author name discrepancy: It is Dmitri Lodygin in the manuscript file and Dimitry Lodygin in the submission system. Please check.

- Please provide a new completed author checklist (for EMBO reports), which you can download from our author guidelines (https://www.embopress.org/page/journal/14693178/authorguide). Please insert page numbers in the checklist to indicate where the requested information can be found in the manuscript. The completed author checklist will also be part of the RPF.

- Regarding data quantification and statistics, please make sure that in the final manuscript file the number "n" for how many independent experiments were performed, their nature (biological versus technical replicates), the bars and error bars (e.g. SEM, SD) and the test used to calculate p-values is indicated in the respective figure legends (for main, EV and Appendix figures). Please also check that all the p-values are explained in the legend, and that these fit to those shown in the figure. Please provide statistical testing where applicable. Please avoid the phrase 'independent experiment', but clearly state if these were biological or technical replicates. Please also indicate (e.g. with n.s.) if testing was performed, but the differences are not significant. In case n=2, please show the data as separate datapoints without error bars and statistics.

See also:
http://www.embopress.org/page/journal/14693178/authorguide#statisticalanalysis

If n<5, please show single datapoints for diagrams. Moreover:
- Please indicate the statistical test used for data analysis in the legends of figures 5g; 6d; EV 5a.
- Please note that information related to n is missing in the legends of figures 5b; 6i; EV 4g-h.
- Please note that the error bars are not defined in the legends of figures 5a; 6d.

- Please add to each legend (main, EV and Appendix figures, where applicable) a 'Data Information' section explaining the statistics used or providing information regarding replicates and scales. See:

https://www.embopress.org/page/journal/14693178/authorguide#figureformat

- Please add scale bars of similar style and thickness to all microscopic images, using clearly visible black or white bars (depending on the background). Please place these in the lower right corner of the images themselves. Please do not write on or near the bars in the image but define the size in the respective figure legend.

- We now use CRediT to specify the contributions of each author in the journal submission system. CRediT replaces the author contribution section. Please use the free text box to provide more detailed descriptions and do NOT provide your final manuscript text file with an author contributions section. See also our guide to authors:
https://www.embopress.org/page/journal/14693178/authorguide#authorshipguidelines

- Please reduce the number of keywords to 5 and order the manuscript sections like this using these names:
Title page - Abstract - Keywords - Introduction - Results - Discussion - Methods - Data availability section (DAS) - Acknowledgements - Disclosure and Competing Interests Statement - References - Figure legends - Expanded View Figure legends

- Please note our reference format (we need up to 10 authors listed before 'et al'). See:
http://www.embopress.org/page/journal/14693178/authorguide#referencesformat

- Please make sure that all the funding information is also entered into the online submission system and is complete and similar to the one in the manuscript text file (in the Acknowledgements). It seems that presently these grant numbers are provided in the Acknowledgments section, but are missing from the submission system: SFB1027, 435235019, 251546152, 411422114, 269925409, 335447717, 408885537. Moreover, these grants seem to be missing in the submission system: European Commission under The European Union's Horizon 2020 research and innovation programme, (grant agreement No. 101021345). Please check.

- Please move all the methods information to the main manuscript text file. We do not allow supplementary methods in the Appendix.

- Please provide the Appendix as pdf file, using the nomenclature "Appendix Figure Sx", "Appendix Table Sx" for the items and their callouts.

- There are EV tables mentioned in the manuscript text file (but no such files have been uploaded). I would suggest showing these tables in the Appendix, using the name "Appendix Table Sx". Please adjust the callouts to these tables accordingly. Finally, please remove the legends for the Tables EV1-EV8 from the manuscript text file.

- Thanks for providing the source data (SD). Please also upload the completed source data checklist as a related manuscript file and each the SD as one ZIPed folder per figure.

- Could you please also provide the source data for the heavily cropped blots in Appendix Fig S4F?

In addition, I would need from you:
- a short, two-sentence summary of the manuscript (not more than 35 words).
- two to four short (!) bullet points highlighting the key findings of your study (two lines each).
- a schematic summary figure as separate file that provides a sketch of the major findings (not a data image) in jpeg or tiff format (with the exact width of 550 pixels and a height of not more than 400 pixels) that can be used as a visual synopsis on our website.

I look forward to seeing a revised version of your manuscript when it is ready. Please let me know if you have questions or comments regarding the revision.

Kind regards,

Achim

Achim Breiling
Senior Editor
EMBO Reports

Referee #1:

The quality of the measurements of mitochondrial membrane potential remains inadequate. Please compare Fig. 2E in the original and revised versions. As mentioned in the comment for authors the pattern for effector cells is identical; and that of naive cells in the revision looks like the original on a different scale (this latter point is harder to assess so I didn't mention it in the comments). I therefore believe that they utilized the same dataset without repeating any experiments. If this is the case, removing the statement about the washing step from the methods is a serious matter, because it has to do with the reproducibility of the experiment.

I am still not convinced by the measurements of mitochondrial membrane potential. I never questioned the use of TMRE, what I wrote is a warning about its limitations (which also apply to JC1) and explained why it is a mistake to wash out the probes. In spite of the different representative trace and ordinate scale, the distribution of the data points of effectors cells in revised Fig. 2E is the same as that of the original version, suggesting that the dataset is the same. The only difference is that the authors removed mention to the washing step from the methods, and I think that this is not acceptable. I therefore must reiterate my criticism - the concentration of TMRM is too high, an inhibitor of the MDR pump was not used and the experiment was not repeated without probe washout. I am aware that these probes are often being used rather carelessly in high-profile publications, but I find this to be a poor excuse.

Referee #2:

As we all know mitochondrial Ca2+ essential for shaping cellular bioenergetics. Recent findings revealed that the major route for mito Ca2+ uptake occurs via MCU channel. Although lymphoid cells glycolytic nature, T-cell receptor (TCR)-induced Ca2+ signals are essential for T-cell activation and function. The functional relevance of the mitochondrial Ca2+ uniporter (MCU)

complex in T-cells, was not fully understood. Here, the authors unveiled the mito Ca2+ uptake in primary naive and effector human CD4+ T cells. Unbiased transcriptome and proteome analyses reveal molecular determinants involved in the TCR-induced functional reprogramming and identify signalling pathways and cellular functions regulated by MCU. Overall, the current study demonstrated that limiting MCU in T cells have a potential tool to alleviate the autoimmune diseases.

The authors have performed several experiments and clarified the reviewer's concerns. The original claim on the role of MCU in T cell function is addressed thoroughly in the revised manuscript.

The revised manuscript is suitable for publication.

Referee #3:

The revised manuscript is modestly improved, but my main concerns have not been addressed.
1. There is no evidence of "functional rearrangement" of MCU complex - this conclusion continues to be without basis. Even transcriptional changes cause only modest changes in protein levels based on the immunoblotting data added by the authors. Reviewer 1 also noted this concern, and this issue remains problematic despite the addition of immunoblotting data. The proteomic data seem completely inconclusive to me from the standpoint of functional rearrangement because such datasets have very poor resolution at the level of protein quantification, and they seem to have no meaningful trend to support this manuscript's conclusions.
2. In my previous review, I outlined specific concerns with mCa2+ measurements and recommended direct measurement of mCa2+ uptake. The authors have not done these experiments and instead added data with other problematic sensors. The FRET based sensors are not an improvement over Pericam and the same concerns remain because they need to be transfected into T cells. Primary T cells are highly recalcitrant to such manipulation and the tiny percentage that is successfully transfected cannot offer typical results due to overexpression and death artifacts - many transfected cells are apoptotic, with highly altered mitochondrial metabolism.
3. The data on EAE model continue to be weak and unconvincing. Authors are adding experimental results that do not address the primary concerns and an increase in data volume does not improve the quality of the manuscript if the main concerns articulated in my previous review remain. In the case of EAE - there is hardly any difference in cytokine profile. The proteomic analysis and HOMER analyses just adds more confusion rather than clarity. Also the quality of NFAT activation data is very unconvincing. The effect size is so small that I find it very hard to believe that this is meaningful.
4. In my previous review I outlined why the EAE model is especially problematic with adoptive transfer and outlined crucial controls. Most specifically, it is important to evaluate the survival of Mcu-depleted T cells. Authors have not done that. Instead, they now find that fewer T cells in the CNS tissue with a complicated day-to-day variance. This would actually be consistent with lowered survival of MCU-depleted T cells. Authors have now concluded that knocking down Mcu has altered the migratory potential of T cells. That is new claim, but without concrete evidence. Migration of T cells needs to be tested ex vivo.
5. Note that previous study of Mcu knockout in T cells does not support any of the claims made in this manuscript. The model being proposed is therefore completely dependent on knockdown studies which can present significant artifacts. These concerns needed very concrete experiments that I outlined but the revised manuscript does not address those concerns. I am left with a strong suspicion that the results of the EAE model are an artifact of adoptive transfer. But that was the only significant claim from the standpoint of in vivo pathophysiology.
6. A recent study (Jost et al., IJMS, 2023) shows that downregulation of MCU impairs suppressive capacity of CD4+ regulatory T cells. This would suggest that downregulation of MCU in T cells potentiates autoimmune disease - opposite of what was claimed in this manuscript. Authors did not discuss the discrepancy. Considering the concerns outlined above, this dampens the enthusiasm for this manuscript in its current form despite the revision.

Point-by-point response - Shumanska et al.

Referee #1 (Comments on Novelty/Model System for Author):
The quality of the measurements of mitochondrial membrane potential remains inadequate. Please compare Fig. 2E in the original and revised versions. As mentioned in the comment for authors the pattern for effector cells is identical; and that of naive cells in the revision looks like the original on a different scale (this latter point is harder to assess so I didn't mention it in the comments). I therefore believe that they utilized the same dataset without repeating any experiments. If this is the case, removing the statement about the washing step from the methods is a serious matter, because it has to do with the reproducibility of the experiment.
We have repeated our mitochondrial membrane potential measurements on naive and effector T-cells (please see our detailed answer to the remark below).

Referee #1 (Remarks for Author):
I am still not convinced by the measurements of mitochondrial membrane potential. I never questioned the use of TMRE, what I wrote is a warning about its limitations (which also apply to JC1) and explained why it is a mistake to wash out the probes. In spite of the different representative trace and ordinate scale, the distribution of the data points of effectors cells in revised Fig. 2E is the same as that of the original version, suggesting that the dataset is the same. The only difference is that the authors removed mention to the washing step from the methods, and I think that this is not acceptable. I therefore must reiterate my criticism - the concentration of TMRM is too high, an inhibitor of the MDR pump was not used and the experiment was not repeated without probe washout. I am aware that these probes are often being used rather carelessly in high-profile publications, but I find this to be a poor excuse.
After reading this remark, together with the initial review, we realized that we might have not fully understood the main concern of the reviewer. Our interpretation was that the mitochondrial volume and mass might affect the measurements and thus the TMRE signal in naive vs effector T-cells. Accordingly, we determined mitochondrial volume and normalized the TMRE signal to the mitochondrial volume in naive and effector T cells.

We would like to note that we did not claim that we performed new measurements. In our reply we clearly stated that the old measurements were used and were normalized to the cell/mitochondrial volume.

Nevertheless, as pointed out in our first reply, we agree that TMRE is not a perfect dye and that it needs to be used with caution. We, hence, performed the measurements exactly as suggested by this reviewer.
We stained the cells with 10 nM TMRE probe in a buffer containing MDR pump inhibitor (Probenicid 50 µM). Washout was not performed, and the TMRE and inhibitor concentrations were kept consistent throughout the measurements. Single cell imaging was used and only the mitochondrial area of the cells was quantified. The new data are now presented in main Figure 2E (please see below). The results again showed that effector T-cells have a higher mitochondrial membrane potential compared to naive T cells.

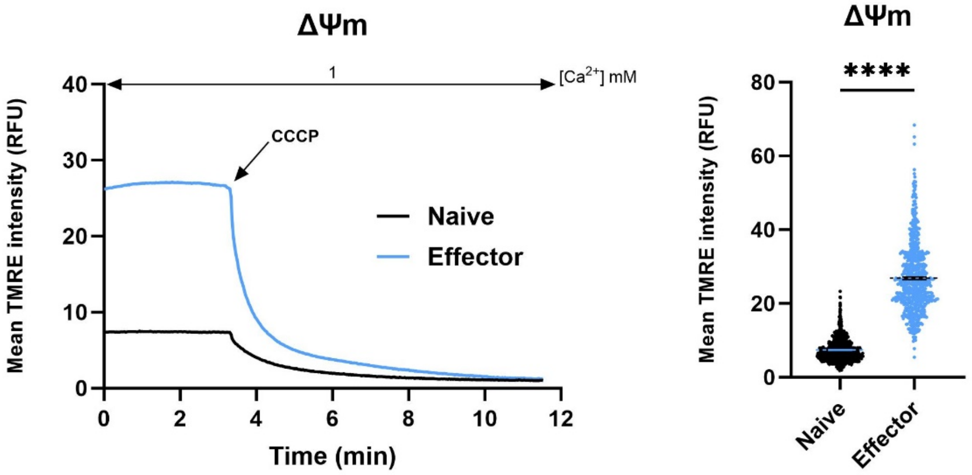

Figure 2. (E) Single cell measurements of ΔΨm using TMRE in naive and effector T-cells. Traces show mean RFU of 782 naive and 678 effector T-cells/biological replicates from 4 healthy donors. The quantification on the right shows mean ± SD of the resting ΔΨm. CCCP: 2 µM. **** - p <0.0001 (p=0), assessed by two-tailed unpaired Student's t-test. RFU=Relative fluorescence units.

We appreciate the criticism and hope that this improves our manuscript and interpretations of alterations in mitochondrial membrane potential upon TCR stimulation.

Referee #2 (Comments on Novelty/Model System for Author):

As we all know mitochondrial Ca^{2+} is essential for shaping cellular bioenergetics. Recent findings revealed that the major route for mito Ca^{2+} uptake occurs via MCU channel. Although lymphoid cells glycolytic nature, T-cell receptor (TCR)-induced Ca^{2+} signals are essential for T-cell activation and function. The functional relevance of the mitochondrial Ca^{2+} uniporter (MCU) complex in T-cells, was not fully understood. Here, the authors unveiled the mito Ca^{2+} uptake in primary naive and effector human CD4+ T cells. Unbiased transcriptome and proteome analyses reveal molecular determinants involved in the TCR-induced functional reprogramming and identify signaling pathways and cellular functions regulated by MCU. Overall, the current study demonstrated that limiting MCU in T cells have a potential tool to alleviate the autoimmune diseases.

No ethical issues.

Referee #2 (Remarks for Author):

The authors have performed several experiments and clarified the reviewer's concerns. The original claim on the role of MCU in T cell function is addressed thoroughly in the revised manuscript.

The revised manuscript is suitable for publication.

We are very thankful for the positive evaluation of our manuscript and for the helpful comments and suggestions.

Referee #3 (Remarks for Author):

The revised manuscript is modestly improved, but my main concerns have not been addressed.

1. There is no evidence of "functional rearrangement" of MCU complex - this conclusion continues to be without basis. Even transcriptional changes cause only modest changes in protein levels based on the immunoblotting data added by the authors. Reviewer 1 also noted this concern and this issue remains problematic despite the addition of immunoblotting data. The proteomic data seem completely inconclusive to me from the standpoint of functional rearrangement because such datasets have very poor resolution at the level of protein quantification, and they seem to have no meaningful trend to support this manuscript's conclusions.

Our data, derived from multiple experimental techniques including qRT-PCR, RNAseq, and proteomics, demonstrated alterations in MCU complex expression and protein abundance. These findings were further corroborated by our immunoblot data.

We acknowledge that additional biochemical and stoichiometric experiments could strengthen the evidence for compositional rearrangements of the MCU complex. Therefore, we have revised our manuscript, removing statements about MCU rearrangement upon T-cell activation and instead referring solely to increased mitochondrial Ca^{2+} levels and elevated MCU activity in effector T-cells compared to naive T-cells.

2. In my previous review, I outlined specific concerns with mCa^{2+} measurements and recommended direct measurement of mCa^{2+} uptake. The authors have not done these experiments and instead added data with other problematic sensors. The FRET based sensors are not an improvement over Pericam and the same concerns remain because they need to be transfected into T cells. Primary T cells are highly recalcitrant to such manipulation and the tiny percentage that is successfully transfected cannot offer typical results due to overexpression and death artifacts - many transfected cells are apoptotic, with highly altered mitochondrial metabolism.

We understand this concern. However, we (and others) have been optimizing the DNA and RNA transfection procedure in primary cells for many years (by using electroporation) and are now able to achieve a transfection efficiency of 60-80% with high T-cell viability and no loss of physiological cell function. Given that the Ca^{2+} measurements are performed 48 hours after transfection, we are certain that only the viable cells are measured (apoptotic cells detach from the coverslips and are unable to respond to external stimuli). We also believe that the resting period of 48 hours between transfection and measurements significantly diminishes possible negative effects on cell metabolism.

We agree that an additional control method of mitochondrial Ca^{2+} measurements in naive and effector T-cells, as well as siRNA-transfected T-cells, could be helpful and performed the measurements as suggested by the reviewer (using a Calcium Retention Capacity (CRC) Assay and digitonin-based plasma membrane perforation). To that end, we re-suspended T-cells into a buffer without Ca^{2+}, containing 15 µM digitonin and 1 µM Calcium-Green-5N probe. We assessed mitochondrial Ca^{2+} uptake by subsequent additions of a bolus of Ca^{2+}-containing buffer onto the cells and measured changes in fluorescence intensity in a plate reader. The results demonstrated that effector T-cells exhibit higher mitochondrial Ca^{2+} uptake compared to naive T-cells (now shown in Fig. EV1K and below). Additionally, siMCUaKD T-cells

displayed significantly reduced mitochondrial Ca^{2+} uptake (as shown in Revision Figure 1 below).

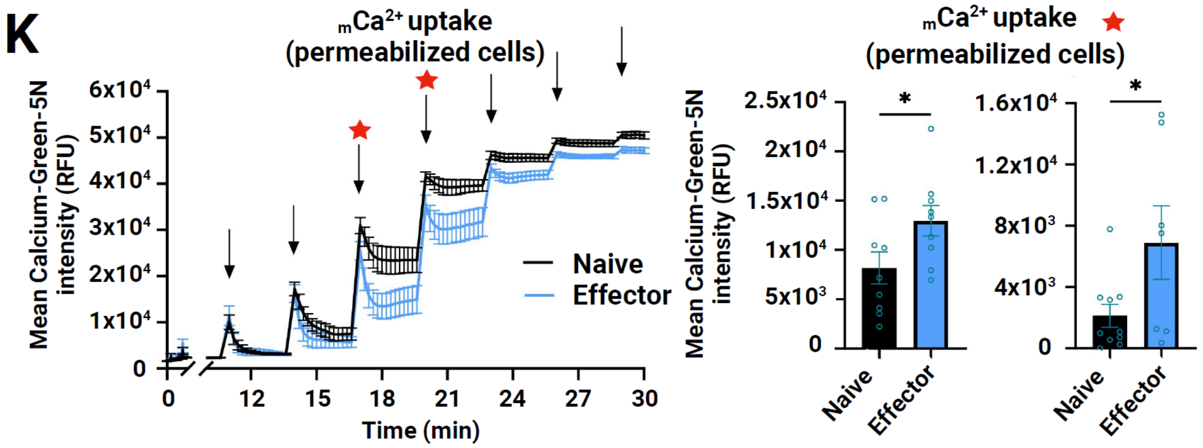

Figure EV1. **(K)** Calcium Retention Capacity (CRC) Assay measuring $_mCa^{2+}$ uptake in digitonin permeabilized naive and effector T-cells using Calcium-Green-5N. Traces show mean ± SEM relative fluorescence units (RFU) of 10 naive and 10 effector T-cell replicates from 3 healthy donors (assays performed at least in duplicate). $_mCa^{2+}$ uptake was induced by subsequent additions of a bolus of Ca^{2+} at the indicated time points (arrows). The quantification plots show mean ± SEM for both basal $_mCa^{2+}$ uptake at the time points indicated by a red star. $_mCa^{2+}$ uptake was quantified as the delta decrease in measured RFU upon Ca^{2+} addition. *- $p \leq 0.05$ (p=0.0481-left; p=0.0443-right panel), assessed by two-tailed unpaired Student's t-test.

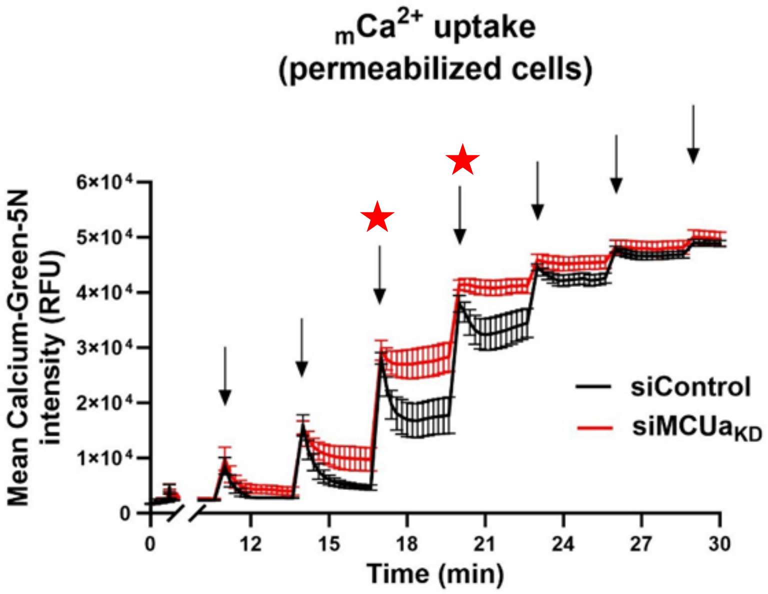

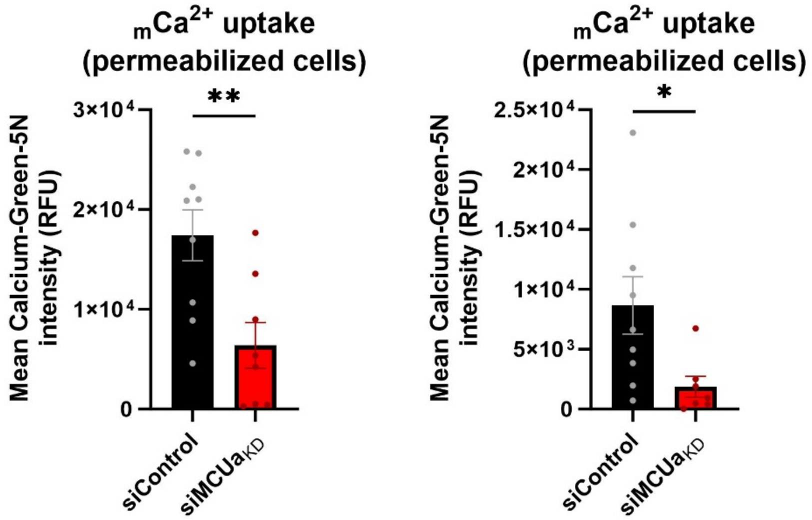

Revision Figure 1. Calcium Retention Capacity (CRC) Assay measuring $_{m}Ca^{2+}$ uptake in digitonin permeabilized siControl and siMCUa$_{KD}$ T-cells using Calcium-Green-5N. Traces show mean ± SEM relative fluorescence intensity of 12 siControl and 11 siMCUa$_{KD}$ T-cell replicates from 2 healthy donors (assays performed at least in duplicate). $_{m}Ca^{2+}$ uptake was induced by subsequent additions of a bolus of Ca^{2+} at the indicated time points (arrows). The quantification plots show mean ± SEM for both basal $_{m}Ca^{2+}$ uptake at the time points indicated by a red star. $_{m}Ca^{2+}$ uptake was quantified as the delta decrease in measured RFU upon Ca^{2+} addition. **- $p < 0.01$ ($p = 0.0063$), *- $p \leq 0.05$ ($p = 0.0316$), assessed by two-tailed unpaired Student's t-test.

3. The data on EAE model continue to be weak and unconvincing. Authors are adding experimental results that do not address the primary concerns and an increase in data volume does not improve the quality of the manuscript if the main concerns articulated in my previous review remain. In the case of EAE - there is hardly any difference in cytokine profile. The proteomic analysis and HOMER analyses just adds more confusion rather than clarity. Also the quality of NFAT activation data is very unconvincing. The effect size is so small that I find it very hard to believe that this is meaningful.

The proteome analyses of the rat control and sgMCUa$_{KD}$ T-cells identified a number of regulated proteins. The detailed bioinformatic analyses suggested that MCU plays an essential role in T-cell migration and corroborated our results obtained with the human siMCUa$_{KD}$ cells (Main Figure 4C). The HOMER-based identification of additional transcription factors controlled by MCU in the rat T-cells will certainly be helpful for future studies. Exploring this in more detail is out of the scope of this manuscript.

We did not evaluate changes in the cytokine profile of rat sgMCUa$_{KD}$ T-cells and have not commented on that, but solely on the effects in human T-cells (IL-2 and IFNγ).

Regarding the NFAT1 measurements, we performed additional quantifications of our existing data to more clearly represent the effect of MCUa downregulation on NFAT1 nuclear translocation in the human T-cells. If we consider that the Tg-induced NFAT1 translocation is the maximum ((siControl + Tg) - (siControl) = 100%), then the siMCUa$_{KD}$-induced inhibition of NFAT1 translocation following Tg treatment is around 38% (for cytosolic NFAT1) and around 42% (for nuclear NFAT1), with a high p-value (now Main Figure 4K). Given that

cytokine secretion is mediated via NFAT signaling, the new findings provide additional mechanistic insights regarding the role of MCU in T-cell function.

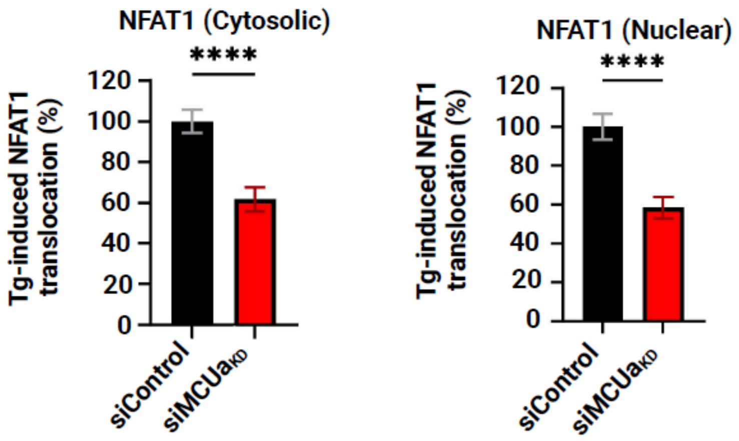

Figure 4. (**K**) Quantification of % of Tg-induced NFAT1 translocation based on data in **J**. Both graphs show mean ± SEM of 61 control and 65 siMCUa$_{KD}$ cells. **** - *p* ≤0.0001 (*p*=0.00001), assessed by two-tailed unpaired Student's t-test.

We hope that this will convince the reviewer that the NFAT results are indeed meaningful.

4. In my previous review, I outlined why the EAE model is especially problematic with adoptive transfer and outlined crucial controls. Most specifically, it is important to evaluate the survival of Mcu-depleted T cells. Authors have not done that. Instead, they now find that fewer T cells in the CNS tissue with a complicated day-to-day variance. This would actually be consistent with lowered survival of MCU-depleted T cells. Authors have now concluded that knocking down Mcu has altered the migratory potential of T cells. That is new claim, but without concrete evidence. Migration of T cells needs to be tested *ex vivo*.

Regarding T-cell survival:

As pointed out in our first point-to-point reply, we have measured numbers of control and sgMCUa$_{KD}$ autoreactive T-cells in different organs over a period of 3 days after co-transfer. During this time, i.v. transferred T-cells quickly leave blood circulation, accumulate in the lung tissue and start to migrate to lung-draining lymph nodes (LNs). Between days 2 and 3 post-transfer, they exit LNs and return to the blood circulation in order to reach and invade the CNS (Odoardi et al., 2012). Thus, the number of T-cells at different time points is a complex parameter that cumulates from T-cell proliferation, trafficking (influx into and efflux from a compartment) and survival. In our revised manuscript, we wrote the following: *Indeed, we observed that fewer MCUa$_{KD}$ T-cells, comparing to control cells, were found in the CNS tissue and in other organs (Fig. 6J-L). Therefore, a compromised survival of MCU-deficient T-cells may partially be responsible for the diminished disease severity.* However, we found that the decreased survival of MCUa$_{KD}$ T-cells (which was not observed *in vitro*) is not a sole and "trivial" reason for reduced EAE severity. Decreased survival cannot explain the divergent ratio of control to MCUa$_{KD}$ T-cell numbers in the blood comparing to other organs. We do agree that the representation of these results is not perfect and decided to generate a new figure which

precisely depicts the effect of MCU-knockdown on rat T-cell survival (see below, now in Main Figure 7).

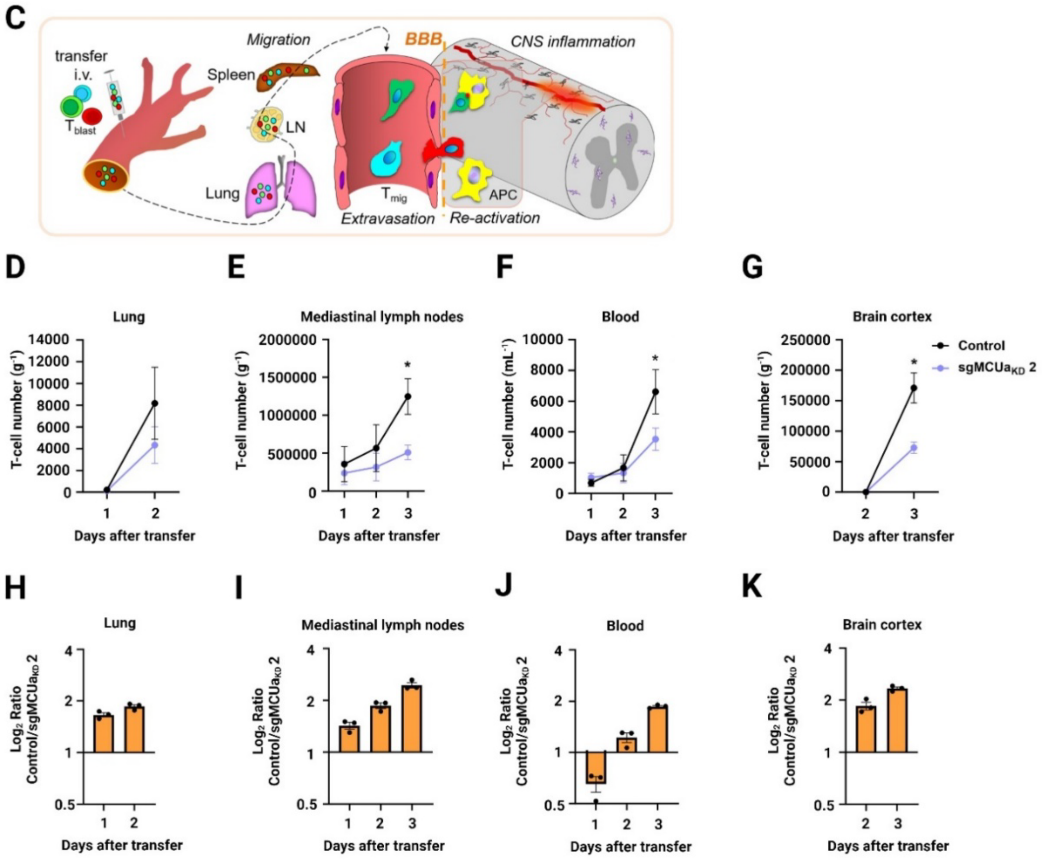

Figure 7. (**C**) T-cell migration pattern after adoptive transfer, prior to induction of clinical EAE. (**D-G**) T-cell numbers quantified *ex vivo* after adoptive T-cell co-transfer in different compartments: (**D**) lungs, (**E**) mediastinal lymph nodes, (**F**) blood, and (**G**) brain cortex. T-cell counts were analysed on a CytoFLEX flow cytometer after staining with anti-CD134 (OX-40)-AF647 and anti-CD25-BV750. Graphs show mean ± SEM (n=3 animals presented as single points) of cell numbers at a given day after transfer. * - $p \leq 0.05$ (p=0.0351 for **E**, p=0.0500 for **F**, p= p=0.0233 for **G**), assessed by two-tailed paired Student's test. (**H-K**) Log_2 Ratio of control and $sgMCUa_{KD}$ T-cells migrating in the different compartments, given as mean ± SEM (n=3 animals presented as single points; based on data in **D-G**) at a certain day after co-transfer.

Regarding T-cell migration:

We do not understand this statement since we measured migration also *ex-vivo* (both in rat and in human T-cells). Please see Figures 4C and 6G in the manuscript. To avoid confusion, we have replaced the existing title with '*Migration/Invasion*' in the panel titles (we modify the standard trans-well migration assay protocol by adding collagen on top of the inserts; therefore, we refer to these measurements as not only migration but also invasion).

5. Note that previous study of Mcu knockout in T cells does not support any of the claims made in this manuscript. The model being proposed is therefore completely dependent on knockdown studies which can present significant artifacts. These concerns needed very concrete experiments that I outlined but the revised manuscript does not address those concerns. I am

left with a strong suspicion that the results of the EAE model are an artifact of adoptive transfer. But that was the only significant claim from the standpoint of in vivo pathophysiology.
Please see our detailed explanations regarding the use of EAE below.

Validity of the model:
The Lewis rat transfer EAE is an extremely robust and reliable model. Fully differentiated encephalitogenic T-cells are transferred to recipient animals, which then develop the classic EAE symptoms. In fact, transfer EAE is even more reliable than active EAE: there is no activation and differentiation of the T cells in the lymph nodes. One can therefore be sure that the number of T-cells and their pathogenic potential are guaranteed. The T-cells are very carefully tested for encephalitogenicity before the EAE experiment. One can therefore, reliably predict and track the course and severity of the disease. It should be noted that rat transfer EAE is clearly superior to mouse transfer EAE (at least in the commonly used C57Bl6 model), since encephalitogenic rat T-cells can be generated and amplified from immunized animals in high numbers and purity. An additional differentiation stimulus (in the mouse by adding cytokines to the T-cell culture) is not necessary. Furthermore, in contrast to mice, PTX treatment of the recipient animals is not necessary in rats. This makes the Lewis rat transfer EAE one of the most reliable CNS autoimmune models, especially for the investigation of effector T-cell function (which is also the purpose of this manuscript).

Benefits of the model:
This EAE model is particularly suitable for testing the properties of effector T-cells because, as explained in detail above, artifacts can largely be excluded. The encephalitogenic T-cells can be genetically modified very efficiently (> 95%, Flügel et al., Nature Medicine, 1999) without their encephalitogenic potential being changed by the gene transfer. In this study, genes for fluorescent proteins (GFP) were introduced to reliably track T-cells *in vivo*. By establishing a Cas9 transgenic rat (not yet published), our group is also able to inactivate specific genes (here MCU) in the effector T-cells. Gene manipulation occurs after immunization in the amplification phase *in vitro*. Therefore, only the encephalitogenic effector T-cells are selectively manipulated. In contrast to the conventional strategies of global or cell-specific KO (as commonly used in mice), the approach used here is therefore significantly less susceptible to artifacts. Not all T-cells are genetically manipulated, but only the pathogenic (fully differentiated) effector T-cells. These "KO" T-cells can also be reliably tracked and functionally characterized *in vivo* through their fluorescence expression.

Interpretation of the results:
We have many years of experience with this model and can therefore very accurately predict the migration and functional changes of the encephalitogenic T-cells after transfer (Flügel et al., Nature Medicine 1999 and Immunity 2001; Kawakami et al., JEM 2004 and 2005; Odoardi et al., PNAS 2007 and Nature 2012; Lodygin et al., Nature 2019; Merlini et al., Nature Neuroscience 2022 and Hosang et al., Nature 2022). We know that the T-cells undergo a complex migration process before inducing clinical EAE. After *i.v.* transfer, the cells first migrate into the lungs, then into the draining mediastinal LNs, before entering the bloodstream and spleen again. Only then do they migrate into the CNS and trigger the clinical disease.

During migration, the T-cells undergo very strong functional changes. First, they fundamentally reprogram their gene expression in the lungs and mediastinal LNs. They adopt a migration mode that allows them to become mobile, adhere to the vessel walls and penetrate the CNS tissue. In the CNS, the T-cells are reactivated by local phagocytes that present them with the specific CNS antigen and thereby trigger the pathogenic autoimmune inflammation of the CNS tissue with the resulting clinical symptoms. The effects of a gene inactivation in effector T-cells can therefore be very reliably studied in this model.
In the case of $MCUa_{KD}$, there are very clear clinical effects, which are also confirmed in two different transfer EAE models (β-synuclein-specific T-cells and MBP-specific T-cells). The fact that the T-cell numbers are reduced in the case of $MCUa_{KD}$, but cells are still present in substantial numbers (approx. 50% reduction) suggests a limitation of T-cell fitness and not a massive death of the cells *in vivo*. It is also striking that the numbers of $MCUa_{KD}$ T-cells are increased in the blood compared to the control T-cells at the early time point after transfer (day 1 after transfer), but decreased in the lungs and mediastinal LNs. Furthermore, we find that in the phase in which the T-cells begin to migrate into the CNS (day 2 after transfer), $MCUa_{KD}$ T-cells, although present in approximately the same number as the control T-cells in the blood, are found in reduced numbers in the CNS. Both findings indicate that the cells obviously have problems with their migration behavior (blood-lung and blood-CNS). These *in vivo* observations are therefore in very good agreement with the *in vitro* findings. $MCUa_{KD}$ restricts the cells' metabolism and thereby reduces their overall fitness; they do not migrate as efficiently and produce less cytokine amounts. This, therefore, very plausibly explains why fewer cells can ultimately migrate into the CNS. As a result, the clinical disease of EAE is not completely blocked, but it is significantly delayed and less severe. In our opinion, it does not make sense to try to explain the reduced encephalitogenic potential of T-cells by a single deficit in function (e.g. reduced cytokine production). Rather, a number of cell-intrinsic factors obviously are in play together.

6. A recent study (Jost et al., IJMS, 2023) shows that downregulation of MCU impairs suppressive capacity of CD4+ regulatory T cells. This would suggest that downregulation of MCU in T cells potentiates autoimmune disease - opposite of what was claimed in this manuscript. Authors did not discuss the discrepancy. Considering the concerns outlined above, this dampens the enthusiasm for this manuscript in its current form despite the revision.
We thank the reviewer for pointing out the discrepancy between this published study and ours. It should be noted that the study by Jost et al. is and was cited in our manuscript. Anyway, we do agree that the discrepancy should be discussed in more details and have modified our discussion accordingly:
To this end, a recent study compared the effects of chronic versus acute deletion of MCU in regulatory T cells (Tregs) and concluded that development of compensatory mechanisms is a plausible explanation for the lack of functional alterations in the Mcufl/flCd4Cre mice (Jost et al, 2023). Furthermore, the authors found that downregulation of MCU impairs the suppressive capacity of Tregs, which can influence EAE. However, we can largely rule out the involvement of Tregs in our experimental system, as the Cas9-mediated MCU knockdown in our study occurs exclusively in fully differentiated effector T-cells, unlike the global T-cell knockdown seen in the mouse model. Based on previous studies and sequencing data from this work, it is

clear that the effector T-cells in our model do not differentiate into Tregs. Therefore, we are confident that the observed effects of MCU knockdown are solely mediated by encephalitogenic Th1/Th17 effector T-cells.

Our human T-cell population is a mixed population of all T-cell subtypes (mainly Th1 and Th2, but some Tregs are also included). To specifically determine discrepancies would require performing all these experiments in the separate $CD4^{+}$ T-cell subtypes, which would be out of the scope of this study.

Dear Prof. Bogeski,

Thank you for the submission of your further revised manuscript to our editorial offices. I have now received the reports from the two referees that were asked to re-evaluate the study, you will find below. As you will see, both referees now support the publication of the study in EMBO reports.

Referee #1 has further suggestions to improve the study, I ask you to address in a final revised manuscript. I consider the suggested experiment in the comment to point 2. ("I recommend to add oligomycin before CCCP to both effector and naïve cells ...) as optional. But in case you already have such data, please add this to the final revised manuscript. Please also provide a final p-b-p-response addressing the remaining referee concerns or suggestions.

Moreover, I have these final editorial requests:

- Please provide direct links for each deposited dataset in the data availability section (DAS) and remove the referee tokes. Please make sure that the datasets are public and accessible lates on the publication date of the manuscript.

- Please provide titles for the EV figures in their legends.

- Please place the scale bars in the lower right corner of the microscopic images. Please do not write on or near the bars in the image but define the size in the respective figure legend.

- Please remove the sentence: "Source data are available online for this figure". From the figure legends. The related source data will be directly linked to the figure and its legend by our typesetters.

- Please add to each legend (main, EV and Appendix figures, where applicable) a 'Data Information' section explaining the statistics used or providing information regarding replicates and scales. See:

https://www.embopress.org/page/journal/14693178/authorguide#figureformat

- Since July 2024 all Materials and Methods for EMBO press articles need to be described in the main text using our 'Structured Methods' format, which is required for all research articles. According to this format, the Methods section should include a Reagents and Tools Table (listing key reagents, experimental models, software, and relevant equipment and including their sources and relevant identifiers), uploaded as separate file, followed by a Methods section in which we encourage the authors to describe their methods using a step-by-step protocol format with bullet points, to facilitate the adoption of the methodologies across labs. More information on how to adhere to this format as well as downloadable templates (.doc) for the Reagents and Tools Table can be found in our author guidelines (section 'Structured Methods'):

https://www.embopress.org/page/journal/14693178/authorguide#structuredmethods

Thus, please move the Appendix tables, that all seem to contain methods-related information, to this table (and remove these tables from the Appendix). Please also update all related callouts to the reagents and tools table.

I look forward to seeing the final revised version of your manuscript when it is ready. Please let me know if you have questions regarding the revision.

Best

Achim Breiling
Senior Editor
EMBO Reports

Referee #1:

As agreed with the Editor I will limit my comments to Figure 2. I acknowledge the new measurements but I must disagree with the Authors' interpretation of the experiments, which are rather consistent with increased mitochondrial biogenesis. I am providing a summary of my suggestions in the form of a revised text for Figure 2 after the specific comments.

• Specific comments:

1. "To examine whether the alterations in mCa2+ are associated with metabolic changes following T-cell activation, we determined mitochondrial respiration (oxygen consumption rates; OCR) and extracellular acidification rate (ECAR) as a marker of glycolytic activity in naive and effector T-cells from three human donors. Our results showed that both OCR and ECAR were higher in effector, compared to naive T-cells (Fig. 2A and 2B). Furthermore, cellular ATP levels (Fig. 2C) as well as mitochondrial H2O2 levels (Fig. 2D) were also increased. Having in mind that increased mCa2+ stimulates mitochondrial respiration, as well as ATP and ROS production, these findings corroborated our observations regarding the role of mCa2+ and, consequentially, MCU in T-cell biology."

Comment: It is not clear what is meant with "metabolic change" and it remarkable that both OCR and ECAR are increased, together with ATP levels. Actually the increase of ECAR is much larger than that of respiration, a finding that is not commented upon but suggests vastly increased glycolytic rates (see also below).

2. "The mitochondrial membrane potential (ΔΨm) is a crucial parameter that controls mitochondrial function and ATP production. Measurements of ΔΨm in the mitochondria of single cells using the indicators TMRE (Fig. 2E) and JC-1 (Fig. 2F) indicated an IMM hyperpolarization in effector T-cells".

Comment: Hyperpolarization is not an appropriate term. If you mean that effectors T-cells have a higher membrane potential than naïve cells based on the fluorescence signal this conclusion is not permissible, and would actually be in conflict with the increased rates of respiration and ATP levels. Compared to the resting level, mitochondria that are synthesizing ATP have a lower, not a higher membrane potential; and a higher membrane potential does not mean a better function (as the Authors seem to be thinking). I recommend to add oligomycin before CCCP to both effector and naïve cells, a treatment (inhibition of ATP synthesis) that indeed hyperpolarizes mitochondria providing a readout of the maximal membrane potential (and allowing to appreciate the dynamic range of the probe response in the two types of cells).

3. "Based on the observed changes in ΔΨm, we next assessed mitochondrial protein translation, a process important for cellular energy supply, by using a 35S methionine labelling assay. Translation of all mitochondria-encoded proteins was increased shortly after T-cell activation (Fig. 2G)."

Comment: I recommend to delete "Based on the observed changes in ΔΨm" and "a process important for cellular energy supply", which is hard to decipher. Increased translation of all mitochondria-encoded proteins probably means increased mitochondrial biogenesis and increased mitochondrial mass, which in my opinion remains the most likely explanation for most of the present observations.

4. "We thus demonstrated that activation of naive T-cells and their consequent differentiation is followed by a robust metabolic and functional reprogramming. Furthermore, our data suggest that mitochondria play a central role in this process."

Comment: I do not understand what is meant with "robust metabolic and functional reprogramming", which is also the title of this paragraph. I am sorry to have to say that this is not an adequate description of the results.

• Proposed revision of the text related to Figure 2

Effector T-cells potentiate oxidative phosphorylation and glycolytic capacity

To examine whether the alterations in mCa2+ are associated with changes of mitochondrial function following T-cell activation, we determined mitochondrial respiration (oxygen consumption rates; OCR) and extracellular acidification rate (ECAR) as a marker of glycolytic activity in naive and effector T-cells from three human donors. Our results showed that both OCR and ECAR were higher in effector, compared to naive T-cells (Fig. 2A and 2B). Furthermore, cellular ATP levels (Fig. 2C) as well as mitochondrial H2O2 levels (Fig. 2D) were also increased. The protonmotive force (the sum of the proton chemical and electrical potential difference) controls mitochondrial function and ATP production. Given that the mitochondrial membrane potential (ΔΨm) is the major driving force for mitochondrial Ca2+ uptake, we performed measurements of ΔΨm in mitochondria of single cells using the indicators TMRE (Fig. 2E) and JC-1 (Fig. 2F). Mitochondria in both cell types readily accumulated the probes, and responded with increased probe uptake (hyperpolarization) to the addition of oligomycin [note: this is the predicted outcome of the experiment I recommend] and with probe release (depolarization) to the addition of uncoupler. A 35S methionine labelling assay revealed that translation of all mitochondria-encoded proteins was increased shortly after T-cell activation (Fig. 2G). The increase was clearly time-dependent, with the highest increase in translation taking place 48 hours after T-cell stimulation. The steady-state expression of proteins expressed in different mitochondrial sub-compartments and with diverse functions, including TACO1, NDUFA9, SDHA, ATP5B, TMBIM5, and AFG3L2 was also increased (Appendix Fig. S2B). These findings suggest that activation of naive T-cells and their consequent differentiation is followed by a strong increase of mitochondrial biogenesis resulting in increased Ca2+ storage capacity and ATP synthesizing ability, and in a large stimulation of the glycolytic flux. This set of changes would therefore match the increased demand for both ATP and metabolites that is required for DNA and protein synthesis in these rapidly proliferating cells.

Referee #2:

The authors have addressed my concerns and the manuscript has been significantly improved. I recommend publishing the manuscript and have no further reservations.

MITOCHONDRIAL CALCIUM UNIPORTER COMPLEX CONTROLS T-CELL-MEDIATED IMMUNE RESPONSES

Shumanska et al.

POINT-BY-POINT REPLY

Dear Prof. Bogeski,

Thank you for the submission of your further revised manuscript to our editorial offices. I have now received the reports from the two referees that were asked to re-evaluate the study, you will find below. As you will see, both referees now support the publication of the study in EMBO reports.

Dear Dr. Breiling,
Thank you very much for your support and valuable feedback. We also want to thank the reviewers for their positive evaluation of our manuscript and their constructive suggestions. We have carefully revised the manuscript in line with the recommendations, as detailed below. We hope that the revised version now meets the standards for publication in EMBO Reports.

Referee #1 has further suggestions to improve the study, I ask you to address in a final revised manuscript. I consider the suggested experiment in the comment to point 2. ("I recommend to add oligomycin before CCCP to both effector and naïve cells ...) as optional. But in case you already have such data, please add this to the final revised manuscript. Please also provide a final p-b-p-response addressing the remaining referee concerns or suggestions.

Please see our reply to reviewer 1.

Moreover, I have these final editorial requests:

- Please provide direct links for each deposited dataset in the data availability section (DAS) and remove the referee tokes. Please make sure that the datasets are public and accessible latest on the publication date of the manuscript.

Done.

- Please provide titles for the EV figures in their legends.

Done.

- Please place the scale bars in the lower right corner of the microscopic images. Please do not write on or near the bars in the image but define the size in the respective figure legend.

We have thoroughly double-checked all figures that include scale bars. In all relevant figures, the scale bars have been positioned in the lower right corner, as recommended, and without accompanying text.

- Please remove the sentence: "Source data are available online for this figure". From the figure legends. The related source data will be directly linked to the figure and its legend by our typesetters.

Done.

- Please add to each legend (main, EV and Appendix figures, where applicable) a 'Data Information' section explaining the statistics used or providing information regarding replicates and scales.

Done. A "Data Information" section is included for each panel, where the relevant statistical analyses are explained, in the respective figure legends where applicable.

- Since July 2024 all Materials and Methods for EMBO press articles need to be described in the main text using our 'Structured Methods' format, which is required for all research articles. According to this format, the Methods section should include a Reagents and Tools Table (listing key reagents, experimental models, software, and relevant equipment and including their sources and relevant identifiers), uploaded as separate file, followed by a Methods section in which we encourage the authors to describe their methods using a step-by-step protocol format with bullet points, to facilitate the adoption of the methodologies across labs. More information on how to adhere to this format as well as downloadable templates (.doc) for the Reagents and Tools Table can be found in our author guidelines (section 'Structured Methods'):
Thus, please move the Appendix tables, that all seem to contain methods-related information, to this table (and remove these tables from the Appendix). Please also update all related callouts to the reagents and tools table.

Done.

Referee #1:

As agreed with the Editor I will limit my comments to Figure 2. I acknowledge the new measurements but I must disagree with the Authors' interpretation of the experiments, which are rather consistent with increased mitochondrial biogenesis. I am providing a summary of my suggestions in the form of a revised text for Figure 2 after the specific comments.

• Specific comments:

1. "To examine whether the alterations in mCa^{2+} are associated with metabolic changes following T-cell activation, we determined mitochondrial respiration (oxygen consumption rates; OCR) and extracellular acidification rate (ECAR) as a marker of glycolytic activity in naive and effector T-cells from three human donors. Our results showed that both OCR and ECAR were higher in effector, compared to naive T-cells (Fig. 2A and 2B). Furthermore, cellular ATP levels (Fig. 2C) as well as mitochondrial H_2O_2 levels (Fig. 2D) were also increased. Having in mind that increased mCa^{2+} stimulates mitochondrial respiration, as well as ATP and ROS production, these findings corroborated our observations regarding the role of mCa^{2+} and, consequentially, MCU in T-cell biology."

Comment: It is not clear what is meant with "metabolic change" and it remarkable that both OCR and ECAR are increased, together with ATP levels. Actually the increase of ECAR is much larger than that of respiration, a finding that is not commented upon but suggests vastly increased glycolytic rates (see also below).

We agree with the reviewer that the increase in OCR, ECAR and ATP production in effector versus naive T-cells is significant. We initially used the term "metabolic change" for simplicity, but we understand that this may have caused confusion. Therefore, we revised the text as suggested to provide clearer and more precise terminology.

2. "The mitochondrial membrane potential ($\Delta\Psi m$) is a crucial parameter that controls mitochondrial function and ATP production. Measurements of $\Delta\Psi m$ in the mitochondria of single cells using the indicators TMRE (Fig. 2E) and JC-1 (Fig. 2F) indicated an IMM hyperpolarization in effector T-cells".

Comment: Hyperpolarization is not an appropriate term. If you mean that effectors T-cells have a higher membrane potential than naïve cells based on the fluorescence signal this conclusion is not permissible, and would actually be in conflict with the increased rates of respiration and ATP levels. Compared to the resting level, mitochondria that are synthesizing ATP have a lower, not a higher membrane potential; and a higher membrane potential does not mean a better function (as the Authors seem to be thinking). I recommend to add oligomycin before CCCP to both effector and naïve cells, a treatment (inhibition of ATP synthesis) that indeed hyperpolarizes mitochondria providing a readout of the maximal

membrane potential (and allowing to appreciate the dynamic range of the probe response in the two types of cells).

We replaced the term “hyperpolarization” with the term “higher membrane potential” and also changed the text as proposed by this reviewer. Given the focus of this study, we would kindly like to refrain from performing additional measurements of mitochondrial membrane potential with oligomycin. We would also like to remind the reviewer that the membrane potential measurements were carried out in cells where mitochondrial Ca^{2+} uptake was not acutely induced. Therefore, we believe the current data sufficiently supports our conclusions.

3. "Based on the observed changes in ΔΨm, we next assessed mitochondrial protein translation, a process important for cellular energy supply, by using a ^{35}S methionine labelling assay. Translation of all mitochondria-encoded proteins was increased shortly after T-cell activation (Fig. 2G)."

Comment: I recommend to delete "Based on the observed changes in ΔΨm" and "a process important for cellular energy supply", which is hard to decipher. Increased translation of all mitochondria-encoded proteins probably means increased mitochondrial biogenesis and increased mitochondrial mass, which in my opinion remains the most likely explanation for most of the present observations.

We agree and adjusted the text accordingly. We also agree with the notion that increased mitochondrial biogenesis plays an important role in some of the observed alterations of the mitochondrial homeostasis. This is why we determined the mitochondrial volume as shown in Figure EV1 F-H and included this statement in our discussion.

4. "We thus demonstrated that activation of naive T-cells and their consequent differentiation is followed by a robust metabolic and functional reprogramming. Furthermore, our data suggest that mitochondria play a central role in this process."

Comment: I do not understand what is meant with "robust metabolic and functional reprogramming", which is also the title of this paragraph. I am sorry to have to say that this is not an adequate description of the results.

As mentioned previously, we used this terminology for simplicity, as the primary focus of this study was to investigate the role of MCU in T-helper cells, their immune responses, and their involvement in autoimmunity. However, we understand the potential for confusion and have revised the text accordingly.

• Proposed revision of the text related to Figure 2

Effector T-cells potentiate oxidative phosphorylation and glycolytic capacity

To examine whether the alterations in mCa^{2+} are associated with changes of mitochondrial function following T-cell activation, we determined mitochondrial respiration (oxygen consumption rates; OCR) and extracellular acidification rate (ECAR) as a marker of glycolytic activity in naive and effector T-cells from three human donors. Our results showed that both OCR and ECAR were higher in effector, compared to naive T-cells (Fig. 2A and 2B). Furthermore, cellular ATP levels (Fig. 2C) as well as mitochondrial H_2O_2 levels (Fig. 2D) were also increased. The protonmotive force (the sum of the proton chemical and electrical potential difference) controls mitochondrial function and ATP production. Given that the mitochondrial membrane potential ($\Delta\Psi m$) is the major driving force for mitochondrial Ca^{2+} uptake, we performed measurements of $\Delta\Psi m$ in mitochondria of single cells using the indicators TMRE (Fig. 2E) and JC-1 (Fig. 2F). Mitochondria in both cell types readily accumulated the probes, and responded with increased probe uptake (hyperpolarization) to the addition of oligomycin [note: this is the predicted outcome of the experiment I recommend] and with probe release (depolarization) to the addition of uncoupler. A ^{35}S methionine labelling assay revealed that translation of all mitochondria-encoded proteins was increased shortly after T-cell activation (Fig. 2G). The increase was clearly time-dependent, with the highest increase in translation taking place 48 hours after T-cell stimulation. The steady-state expression of proteins expressed in different mitochondrial sub-compartments and with diverse functions, including TACO1, NDUFA9, SDHA, ATP5B, TMBIM5, and AFG3L2 was also increased (Appendix Fig. S2B). These findings suggest that activation of naive T-cells and their consequent differentiation is followed by a strong increase of mitochondrial biogenesis resulting in increased Ca^{2+} storage capacity and ATP synthesizing ability, and in a large stimulation of the glycolytic flux. This set of changes would therefore match the increased demand for both ATP and metabolites that is required for DNA and protein synthesis in these rapidly proliferating cells.

Thank you for the suggestion. We have modified the text accordingly. Please refer to Page 6 in the results section.

Referee #2:

The authors have addressed my concerns and the manuscript has been significantly improved. I recommend publishing the manuscript and have no further reservations.

We thank the reviewer for the positive evaluation of our manuscript.

Prof. Ivan Bogeski
University Medical Center Göttingen
Physiology
Humboldtallee 23
Göttingen 37073
Germany

Dear Prof. Bogeski,

I am very pleased to accept your manuscript for publication in the next available issue of EMBO reports. Thank you for your contribution to our journal.

Your manuscript will be processed for publication by EMBO Press. It will be copy edited and you will receive page proofs prior to publication. Please note that you will be contacted by Springer Nature Author Services to complete licensing and payment information.

You may qualify for financial assistance for your publication charges - either via a Springer Nature fully open access agreement or an EMBO initiative. Check your eligibility: https://www.embopress.org/page/journal/14693178/authorguide#chargesguide

Should you be planning a Press Release on your article, please get in contact with embo_production@springernature.com as early as possible in order to coordinate publication and release dates.

If you have any questions, please do not hesitate to contact the Editorial Office. Thank you for your contribution to EMBO Reports.

Yours sincerely,

Achim Breiling
Senior Editor
EMBO Reports

>>> Please note that it is EMBO Reports policy for the transcript of the editorial process (containing referee reports and your response letter) to be published as an online supplement to each paper. If you do NOT want this, you will need to inform the Editorial Office via email immediately. More information is available here: https://www.embopress.org/transparent-process#Review_Process

EMBO Press Author Checklist

Corresponding Author Name: Ivan Bogeski	
Journal Submitted to: EMBO Reports	
Manuscript Number: EMBOR-2024-59680	

USEFUL LINKS FOR COMPLETING THIS FORM
[The EMBO Journal - Author Guidelines]
[EMBO Reports - Author Guidelines]
[Molecular Systems Biology - Author Guidelines]
[EMBO Molecular Medicine - Author Guidelines]

Reporting Checklist for Life Science Articles (updated January
This checklist is adapted from Materials Design Analysis Reporting (MDAR) Checklist for Authors. MDAR establishes a minimum set of requirements in transparent reporting in the life sciences (see Statement of Task: 10.31222/osf.io/9sm4x). Please follow the journal's guidelines in preparing your
Please note that a copy of this checklist will be published alongside your article.

Abridged guidelines for figures
1. Data
The data shown in figures should satisfy the following conditions:
- the data were obtained and processed according to the field's best practice and are presented to reflect the results of the experiments in an accurate and unbiased manner.
- ideally, figure panels should include only measurements that are directly comparable to each other and obtained with the same assay.
- plots include clearly labeled error bars for independent experiments and sample sizes. Unless justified, error bars should not be shown for technical
- if n<5, the individual data points from each experiment should be plotted. Any statistical test employed should be justified.
- Source Data should be included to report the data underlying figures according to the guidelines set out in the authorship guidelines on Data

2. Captions
Each figure caption should contain the following information, for each panel where they are relevant:
- a specification of the experimental system investigated (eg cell line, species name).
- the assay(s) and method(s) used to carry out the reported observations and measurements.
- an explicit mention of the biological and chemical entity(ies) that are being measured.
- an explicit mention of the biological and chemical entity(ies) that are altered/varied/perturbed in a controlled manner.
- the exact sample size (n) for each experimental group/condition, given as a number, not a range;
- a description of the sample collection allowing the reader to understand whether the samples represent technical or biological replicates (including how many animals, litters, cultures, etc.).
- a statement of how many times the experiment shown was independently replicated in the laboratory.
- definitions of statistical methods and measures:
  - common tests, such as t-test (please specify whether paired vs. unpaired), simple χ2 tests, Wilcoxon and Mann-Whitney tests, can be unambiguously identified by name only, but more complex techniques should be described in the methods section;
  - are tests one-sided or two-sided?
  - are there adjustments for multiple comparisons?
  - exact statistical test results, e.g., P values = x but not P values < x;
  - definition of 'center values' as median or average;
  - definition of error bars as s.d. or s.e.m.

Please complete ALL of the questions below.
Select "Not Applicable" only when the requested information is not relevant for your study.

Materials

Newly Created Materials	Information included in the manuscript?	In which section is the information available? (Reagents and Tools Table, Materials and Methods, Figures, Data Availability Section)
New materials and reagents need to be available; do any restrictions apply?	Not Applicable	

Antibodies	Information included in the manuscript?	In which section is the information available? (Reagents and Tools Table, Materials and Methods, Figures, Data Availability Section)
For **antibodies** provide the following information:<br>- Commercial antibodies: RRID (if possible) or supplier name, catalogue number and or/clone number<br>- Non-commercial: RRID or citation	Yes	This information is provided in the "Materials and Protocols" section, under "Immunoblotting" and in the Reagents and Tools Table

DNA and RNA sequences	Information included in the manuscript?	In which section is the information available? (Reagents and Tools Table, Materials and Methods, Figures, Data Availability Section)
**Short novel DNA or RNA including primers, probes**: provide the sequences.	Yes	This information is available in the Reagents and Tools Table. Details are given in the respective Methods sections.

Cell materials	Information included in the manuscript?	In which section is the information available? (Reagents and Tools Table, Materials and Methods, Figures, Data Availability Section)
**Cell lines:** Provide species information, strain. Provide accession number in repository **OR** supplier name, catalog number, clone number, and/**OR** RRID.	Yes	Packaging cell lines GP+E86 cells (Markowitz et al., 1988). Information under Methods: "Cloning of expression constructs for targeting rat MCU gene and generation of retroviral packaging cell lines".
**Primary cultures:** Provide species, strain, sex of origin, genetic modification status.	Yes	Human T-cells were obtained from human peripheral blood mononuclear cells (PBMCs) of anonymous healthy thrombocyte donors of the local blood bank. Information under Materials and Protocols. Genetic
Report if the cell lines were recently **authenticated** (e.g., by STR profiling) and tested for mycoplasma contamination.	Not Applicable	

Experimental animals	Information included in the manuscript?	In which section is the information available? (Reagents and Tools Table, Materials and Methods, Figures, Data Availability Section)
**Laboratory animals or Model organisms:** Provide species, strain, sex, age, genetic modification status. Provide accession number in repository **OR** supplier name, catalog number, clone number, **OR** RRID.	Yes	Lewis rats were used to perform in vivo adoptive T-cell transfer. All experiments were performed according to the local animal welfare regulations. Both male and female rats were used in the EAE experiments. Information available under "Experimental autoimmune encephalomyelitis (EAE) induction in rats"
**Animal observed in or captured from the field:** Provide species, sex, and age where possible.	Not Applicable	
Please detail **housing and husbandry conditions**.	Yes	Information briefly provided in the "Materials and Protocols" section, subsection "Experimental autoimmune encephalomyelitis (EAE) induction

Plants and microbes	Information included in the manuscript?	In which section is the information available? (Reagents and Tools Table, Materials and Methods, Figures, Data Availability Section)
**Plants:** provide species and strain, ecotype and cultivar where relevant, unique accession number if available, and source (including location for collected wild specimens).	Not Applicable	
**Microbes:** provide species and strain, unique accession number if available, and source.	Not Applicable	

Human research participants	Information included in the manuscript?	In which section is the information available? (Reagents and Tools Table, Materials and Methods, Figures, Data Availability Section)
If collected and within the bounds of privacy constraints report on age, sex and gender or ethnicity for all study participants.	Not Applicable	

Core facilities	Information included in the manuscript?	In which section is the information available? (Reagents and Tools Table, Materials and Methods, Figures, Data Availability Section)
If your work benefited from core facilities, was their service mentioned in the acknowledgments section?	Yes	Information available in "Acknowledgements".

Design

Study protocol	Information included in the manuscript?	In which section is the information available? (Reagents and Tools Table, Materials and Methods, Figures, Data Availability Section)
If study protocol has been **pre-registered, provide DOI in the manuscript**. For clinical trials, provide the trial registration number **OR** cite DOI.	Not Applicable	
Report the **clinical trial registration number** (at ClinicalTrials.gov or equivalent), where applicable.	Not Applicable	

Laboratory protocol	Information included in the manuscript?	In which section is the information available? (Reagents and Tools Table, Materials and Methods, Figures, Data Availability Section)
Provide DOI OR other citation details if **external detailed step-by-step protocols** are available.	Yes	https://doi.org/10.1038/s41586-019-0964-2 https://doi.org/10.1016/j.celrep.2020.108292 https://doi.org/10.1126/scisignal.2000672 Also refer to "Materials and

Experimental study design and statistics	Information included in the manuscript?	In which section is the information available? (Reagents and Tools Table, Materials and Methods, Figures, Data Availability Section)
Include a statement about **sample size** estimate even if no statistical methods were used.	Not Applicable	
Were any steps taken to minimize the effects of subjective bias when allocating animals/samples to treatment (e.g. **randomization procedure**)? If yes, have they been described?	Not Applicable	
Include a statement about **blinding** even if no blinding was done.	Yes	No blinding was done.
Describe **inclusion/exclusion criteria** if samples or animals were excluded from the analysis. Were the criteria pre-established?<br><br>If sample or data points were omitted from analysis, report if this was due to attrition or intentional exclusion and provide justification.	Not Applicable	
For every figure, are **statistical tests** justified as appropriate? Do the data meet the assumptions of the tests (e.g., normal distribution)? Describe any methods used to assess it. Is there an estimate of variation within each group of data? Is the variance similar between the groups that are being statistically compared?	Yes	Refer to the "Materials and Protocols" section, subsection "Statistical analyses". Specific statistical tests used are indicated for each panel in every figure legend, along with the respective exact p-values.

Sample definition and in-laboratory replication	Information included in the manuscript?	In which section is the information available? (Reagents and Tools Table, Materials and Methods, Figures, Data Availability Section)
In the figure legends: state number of times the experiment was **replicated** in laboratory.	Yes	This information is available for each figure in the respective figure legend.
In the figure legends: define whether data describe **technical or biological replicates**.	Yes	All data describe biological repeats. This information is available for each figure in the respective figure legend.

Ethics

Ethics	Information included in the manuscript?	In which section is the information available? (Reagents and Tools Table, Materials and Methods, Figures, Data Availability Section)
Studies involving **human participants**: State details of **authority granting ethics approval** (IRB or equivalent committee(s), provide reference number for approval.	Yes	Refer to the "Materials and Protocols" section, subsection "Primary human CD4+ T-cells".
Studies involving **human participants**: Include a statement confirming that **informed consent** was obtained from all subjects and that the experiments conformed to the principles set out in the WMA Declaration of Helsinki and the Department of Health and Human Services Belmont Report.	Yes	Refer to the "Materials and Protocols" section, subsection "Primary human CD4+ T-cells".
Studies involving **human participants:** For publication of **patient photos**, include a statement confirming that consent to publish was obtained.	Not Applicable	
Studies involving experimental **animals**: State details of **authority granting ethics approval** (IRB or equivalent committee(s), provide reference number for approval. Include a statement of compliance with ethical regulations.	Yes	All experiments were performed according to the local animal welfare regulations of the University of Göttingen, Germany.
Studies involving **specimen and field samples:** State if relevant **permits** obtained, provide details of authority approving study; if none were required, explain why.	Not Applicable	

Dual Use Research of Concern (DURC)	Information included in the manuscript?	In which section is the information available? (Reagents and Tools Table, Materials and Methods, Figures, Data Availability Section)
Could your study fall under dual use research restrictions? Please check biosecurity documents and list of **select agents and toxins** (CDC): https://www.selectagents.gov/sat/list.htm	Not Applicable	
If you used a select agent, is the security level of the lab appropriate and reported in the manuscript?	Not Applicable	
If a study is subject to dual use research of concern regulations, is the name of the **authority granting approval and reference number** for the regulatory approval provided in the manuscript?	Not Applicable	

Reporting

The MDAR framework recommends adoption of discipline-specific guidelines, established and endorsed through community initiatives. Journals have their own policy about requiring specific guidelines and recommendations to complement MDAR.

Adherence to community standards	Information included in the manuscript?	In which section is the information available? (Reagents and Tools Table, Materials and Methods, Figures, Data Availability Section)
State if relevant guidelines or checklists (e.g., **ICMJE, MIBBI, ARRIVE, PRISMA**) have been followed or provided.	Not Applicable	
For **tumor marker prognostic studies,** we recommend that you follow the **REMARK** reporting guidelines (see link list at top right). See author guidelines, under 'Reporting Guidelines'. Please confirm you have followed these guidelines.	Not Applicable	
For **phase II and III randomized controlled trials**, please refer to the **CONSORT** flow diagram (see link list at top right) and submit the CONSORT checklist (see link list at top right) with your submission. See author guidelines, under 'Reporting Guidelines'. Please confirm you have submitted this list.	Not Applicable	

Data Availability

Data availability	Information included in the manuscript?	In which section is the information available? (Reagents and Tools Table, Materials and Methods, Figures, Data Availability Section)
Have **primary datasets** been deposited according to the journal's guidelines (see 'Data Deposition' section) and the respective accession numbers provided in the Data Availability Section?	Yes	RNA sequencing and Proteomics datasets are provided in their specific repository. Refer to "Data Availability" section of the manuscript.
Were **human clinical and genomic datasets** deposited in a public access-controlled repository in accordance to ethical obligations to the patients and to the applicable consent agreement?	Not Applicable	
Are **computational models** that are central and integral to a study available without restrictions in a machine-readable form? Were the relevant accession numbers or links provided?	Not Applicable	
If publicly available data were reused, provide the respective **data citations in the reference list**.	Yes	Refer to the "Materials and Protocols" section, subsections "RNA sequencing" and "Proteomics" in the manuscript, as well as References.
